# Safety and feasibility of 4-1BB co-stimulated CD19-specific CAR-NK cell therapy in refractory/relapsed large B cell lymphoma: a phase 1 trial

**DOI:** 10.1038/s43018-025-00940-3

**Published:** 2025-04-18

**Authors:** Wen Lei, Hui Liu, Wenhai Deng, Wei Chen, Yun Liang, Wenxia Gao, Xianggui Yuan, Shanshan Guo, Ping Li, Jinyong Wang, Xiangmin Tong, Yi Eve Sun, Aibin Liang, Wenbin Qian

**Affiliations:** 1 Department of Hematology, the Second Affiliated Hospital, College of Medicine, Zhejiang University, Hangzhou, China; 2 Key Laboratory of Cancer Prevention and Intervention, China National Ministry of Education; Biotherapy Research Center, the Second Affiliated Hospital, College of Medicine, Zhejiang University, Hangzhou, China; 3 Key Laboratory of Laboratory Medicine, Ministry of Education, School of Laboratory Medicine and Life Sciences, Wenzhou Medical University, Wenzhou, China; 4 Stem Cell Translational Research Center, Tongji Hospital, Tongji University, School of Medicine, Shanghai, China; 5 Department of Hematology, Tongji Hospital of Tongji University, Shanghai, China; 6 State Key Laboratory of Stem Cell and Reproductive Biology, Institute for Stem Cell and Regeneration, Institute of Zoology, Chinese Academy of Sciences, Beijing, China; 7 Department of Hematology, Affiliated Hangzhou First People’s Hospital, School of Medicine, Westlake University, Hangzhou, China

**Keywords:** B-cell lymphoma, Cancer immunotherapy, Cancer

## Abstract

Chimeric antigen receptor (CAR)-modified NK (CAR-NK) cells are candidates for next-generation cancer immunotherapies. Here we generated CD19-specific CAR-NK cells with 4-1BB and CD3ζ signaling endo-domains (CD19-BBz CAR-NK) by transduction of cord blood-derived NK cells using baboon envelope pseudotyped lentiviral vectors and demonstrated their antitumor activity in preclinical B cell lymphoma models in female mice. We next conducted a phase 1 dose-escalation trial involving repetitive administration of CAR-NK cells in 8 patients with relapsed/refractory large B cell lymphoma (NCT05472558). Primary end points were safety, maximum tolerated dose, and overall response rate. Secondary end points included duration of response, overall survival, and progression-free survival. No dose-limiting toxicities occurred, and the maximum tolerated dose was not reached. No cases of cytokine release syndrome, neurotoxicity, or graft-versus-host disease were observed. Results showed an overall response rate of 62.5% at day 30, with 4 patients (50%) achieving complete response. The median progression-free survival was 9.5 months, and the median overall survival was not reached. A post hoc exploratory single-cell RNA sequencing analysis revealed molecular features of CAR-NK cells associated with therapeutic efficacy and efficacy-related immune cell interaction networks. This study met the pre-specified end points. In conclusion, CD19-BBz CAR-NK cells were feasible and therapeutically safe, capable of inducing durable response in patients with B cell lymphoma.

## Abstract (web summary)

Lei et al. develop a 4-1BB co-stimulated anti-CD19 CAR-NK cell product, show efficacy in pre-clinical models, and test safety and feasibility in a phase 1 trial in patients with refractory/relapsed large B cell lymphoma.

## Main

Chimeric antigen receptor (CAR) T-cell therapy has proven effective against hematologic malignancies and is showing promising efficacy and potential for treating solid tumors^1–5^. However, autologous CAR T cells have several drawbacks, including high cost of cellular production, undesirable qualities reported for T cells collected via lymphaphereisis, and serious toxicity such as cytokine release syndrome (CRS) and immune effector cell-associated neurotoxicity syndrome (ICANS)^6,7^, thereby limiting clinical applications of autologous CAR T cells. Although allogeneic CAR T-cell therapy has been developed for its therapeutic potential and economical standardized production, it might introduce a high risk of graft-versus-host disease (GvHD) and be rapidly eliminated by recipients’ immune systems^8–10^.

Natural killer (NK) cells do not bear the risks of GvHD and offer the potential for standardized production as an “off-the-shelf” cellular product, which makes them very attractive as candidates for next-generation cancer immunotherapies^11,12^. CAR-modified NK (CAR-NK) cells possess some advantages over CAR T therapy, such as better safety features and enhanced tumor killing activities through both CAR-dependent and -independent mechanisms^8,13–15^. In preclinical studies, CAR-NK cells have shown impressive activity against various malignancies and are currently being investigated in multiple trials^16–19^. As of June 2024, there are 94 studies registered on clinicaltrials.gov attempting to evaluate the safety and efficacy of various kinds of CAR-NK therapeutic approaches. However, the results from most of these trials are still pending; only a few of these studies have been published, with relatively small numbers of patients enrolled^20–22^. In 2020, Rezvani and colleagues reported results from the “first-in-human” trial of CD19-specific CAR-NK cells derived from umbilical cord blood in 11 heavily pretreated B-cell malignancies^23^. Their CAR-NK cells were established with a retroviral vector encoding, in tandem, an anti-CD19 single-chain fragment variable (scFv), a CD28.CD3ζ endo-domain, secretory interleukin (sIL)-15, and inducible caspase 9, serving as a safety switch (CD19-28z CAR-NK cells). Recently, the authors have presented the data of their phase 1/2 study in 37 patients with relapsed or refractory (R/R) B-cell malignancies^24^. The results showed that CD19-28z CAR-NK cells have a similar efficacy profile to autologous CD19-CAR T cells. Unfortunately, the complete response (CR) rate in diffuse large B cell lymphoma (DLBCL) subgroup was only 29%, which is lower than that reported for CD19-CAR T-cell therapy (39–65%)^5^.

In this Article, we report the generation of CD19-specific CAR-NK cells from cord blood using baboon envelope pseudo typed lentiviral vectors (BaEV-LV). This anti-CD19 CAR construct harbors FMC63 scFv together with the 4-1BB and CD3ζ signaling endo-domains (CD19-BBz CAR), and transgenic expression of sIL-15. Our preclinical study provided the evidence of strong antitumor efficacy of CD19-BBz CAR-NK cells against B cell lymphoma in vitro and in vivo. We next conducted a phase 1 trial, in which patients with R/R large B cell lymphoma (LBCL) received CD19-BBz CAR-NK cells that were administered in a three-dose regimen, and evaluated the clinical safety and efficacy of this CAR-NK cell therapy. Moreover, we performed detailed characterization of CAR-NK cell products administered to the patients with different outcomes after CAR-NK therapy and peripheral blood immune cells of the patients using single-cell RNA sequencing (scRNA-seq) analysis, hence expanding our understanding of resistance mechanisms to CAR-NK cell therapy.

## Results

### CD19-BBz CAR-NK cells showed strong antitumor activity in preclinical lymphoma models

**Fig. 1:**
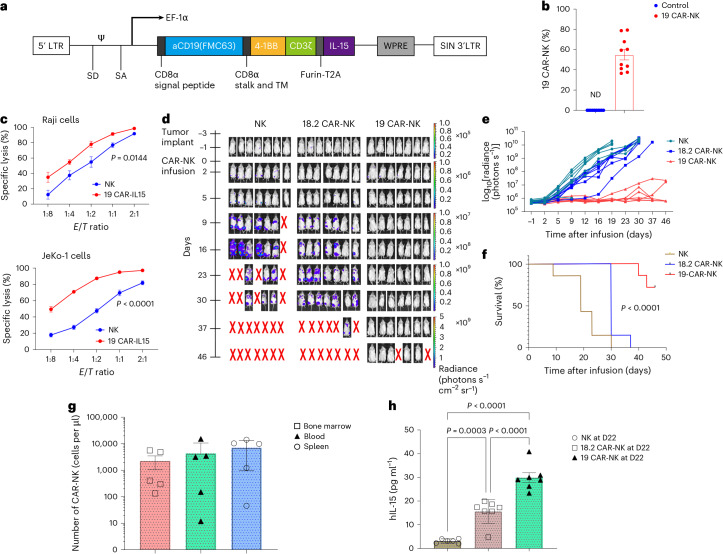
CD19-BBz CAR-NK cells showed strong antitumor activity. **a**, Schematic representation of CD19-BBz CAR lentiviral vector, including a CD8 signal peptide, anti-CD19 scFv, CD8a stalk 4-1BB co-stimulatory, and CD3ζ domain. A sIL-15 followed the CAR via T2A. LTR, long terminal repeat; TM, transmembrane. Ψ, RNA packaging signal; EF-1α, elongation factor 1-alpha 1; SD, splice donor; SA, splice acceptor; WPRE, woodchuck hepatitis virus post-transcriptional regulatory element; SIN, self-inactivating. **b**, CAR^+^ NK cells frequency from 10 independent cord blood units as measured by flow cytometry. Data are expressed as mean ± s.e.m. (*n* = 10). ND, not detected. CD56^+^CAR^+^ NK cells were gated in the hCD45^+^ cells listed in Supplementary Fig. 1a (upper panel). **c**, Cytotoxicity of CD19-BBz CAR-NK cells compared with un-transfected NK cells on Raji or JeKo-1 cells at the indicated *E*/*T* ratios as measured by luciferase-based cytotoxicity assay. Data are expressed as mean ± s.e.m. (*n* = 4 biologically independent experiments). Two-way repeated measures analysis of variance (ANOVA) (Bonferroni posttest) was used. **d**,**e**, Antitumor abilities of CD19-BBz CAR-NK in JeKo-1-Luc cell-inoculated NSG mice model. Tumor burden was measured by bioluminescence imaging (**d**, *n* = 7) and total flux (**e**, *n* = 7) within 46 days. Tumor cells were implanted on day −3, and CAR-NK cells were administered on day 0. **f**, Survival of JeKo-1-bearing mice in each group (*n* = 7) was analyzed by Kaplan–Meier by the log-rank test. **g**, Absolute numbers of CAR^+^ NK cells in blood, spleen, and bone marrow measured by flow cytometry at day 46. Data are presented as mean ± s.e.m. (*n* = 5 mice). Gating strategies are presented in Supplementary Fig. 1b. GFP^+^ cells represent the tumor cells. CD56^+^ CAR^+^ NK cells were gated in the mCD45^−^hCD45^+^GFP^−^ cells. **h**, IL-15 secretion from mice plasma at day 22 after CAR-NK infusion (*n* = 6 in NK group, *n* = 7 in 18.2-BBz CAR-NK and CD19-BBz CAR-NK group). One-way ANOVA test was used. Data are presented as mean ± s.e.m. Source data

**Extended Data Fig. 1:**
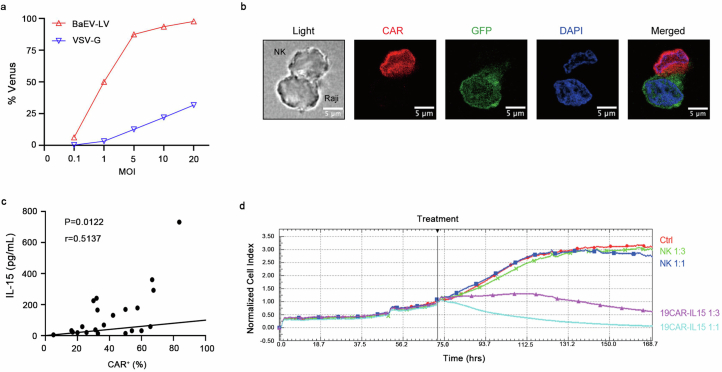
The characteristics of CD19-specific CAR-NK cells generated by BaEV-LV. **a**, Transduction efficiency of BaEV-LV was analyzed in comparison to the VSV-G-pseudotyped lentivirus vector (VSV-G), measured as percentage of Venus in NK92MI cells by flow cytometry. The gate strategies were showed in Supplementary Fig. 1d. Multiplicity of infection (MOI) was defined based on the titration measurement performed on Jurkat cells using serial vector dilutions. Representative data from 2 independent experiments are shown. **b**, Representative fluorescent confocal microscopy images of CD19-BBz CAR-NK cells interacting with Raji cells. Raji cells were transduced to express GFP fused firefly luciferase (in green), and NK cells were labeled with anti-mouse FMC63 scFv antibody (Alexa Fluor 647, in red). The data are representative of four biologically independent experiments. **c**, Correlation between the amount of secretory interleukin (IL)-15 in the culture medium and CD19-BBz CAR-NK cell percentage in total NK cells (n = 23, Spearman’s coefficient r = 0.5137, *P* = 0.0122). Spearman correlation analysis was used. **d**, Cytotoxicity of CD19-BBz CAR-NK cells was measured in real-time, against the target cells NIH/3T3-CD19 at 1:1 or 1:3 effector-to-target (E: T) ratios at day 4 by Real Time Cellular Analysis (RTCA) platform. Assay performed in quadruplicate. Representative data from 2 biologically independent experiments are presented. Source data

Both 4-1BB and CD3ζ domains are used naturally by NK cells for signaling and activation^25^. Therefore, we generated a tandem construct containing the CAR with 4-1BB/CD3ζ activation domain (Fig. 1a). The CD19-BBz CAR, which also expressed the interleukin-15 (IL-15) transgene, was transduced by a BaEV-LV vector. High transduction efficiency of cord blood-derived NK cells was shown by the expression of Venus (Extended Data Fig. 1a) and CD19-CAR proteins (Fig. 1b), respectively, on the cell surface. On average, 54.95% of NK cells express CD19-CAR. The formation of immune synapses was observed when Raji cells were co-cultured with our CAR-NK cells (Extended Data Fig. 1b). The levels of secreted IL-15 in the supernatant of CD19-BBz CAR-NK cell cultures were associated with the number of CAR-NK cells (Extended Data Fig. 1c).

We found that CD19-BBz CAR-NK cells were highly more effective compared to un-transfected NK cells in killing Raji and Jeko-1 lymphoma cells (Fig. 1c and Extended Data Fig. 1d). To investigate whether CD19-BBz CAR-NK cells have maintained the enhanced antitumor abilities in vivo, JeKo-1 were intravenously injected into NOD-Prkdcscid IL2rgtm1/Bcgen (NSG) mice. Three days later, each mouse received a single dose of either CD19-BBz CAR-NK or claudin18.2-specific CAR-NK (18.2-BBz CAR-NK) cells that also expressed IL-15 administered via the tail vein. Consistent with their higher potency in vitro, CD19-BBz CAR-NK cells were most effective in killing tumor cells. Five out of seven mice treated with CD19-BBz CAR-NK cells became tumor-free for at least 46 days after treatment, which resulted in significantly improved survival compared to non-modified NK cell or 18.2-BBz CAR-NK treatment (Fig. 1d–f). We next assessed the persistence and infiltration of CD19-BBz CAR-NK cells in vivo by analyzing the bone marrow, blood, and spleen of each mouse for the quantities of these cells at day 46 after infusion and observed that CAR^+^ NK cells were detectable in all samples (Fig. 1g). The average percentage of CAR^+^ cells within tumor-negative human grafts (gated as hCD45^+^GFP^−^) in the bone marrow, blood, and spleen was 94.38%, 92.07%, and 95.05%, respectively. Both CD19-BBz CAR-NK and 18.2-BBz CAR-NK cells were capable of secreting higher levels of IL-15 at day 22, compared to un-transfected NK control (Fig. 1h). These data show that the prolonged persistence and tumor infiltration abilities of CD19-BBz CAR-NK cells might contribute to greater anti-lymphoma efficacy in vivo.

### Clinical trial design and participants

**Fig. 2:**
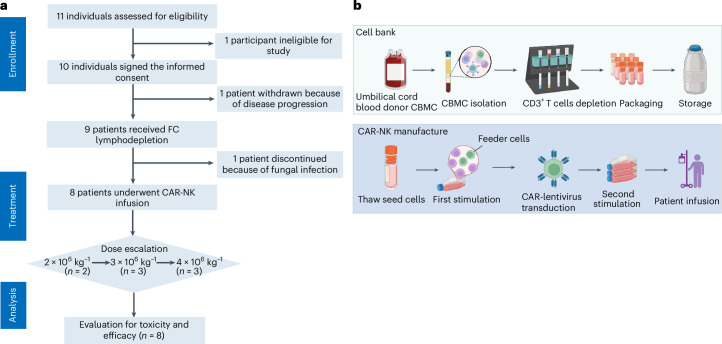
Flow diagram of the clinical trial and the process of CAR-NK manufacture. **a**, Consolidated Standards of Reporting Trials flow chart summarizing the number of participants screened, enrolled, treated, and followed in the study. **b**, CBMCs were isolated from the umbilical cord blood by Ficoll density gradient centrifugation, and CD3^+^ T cells were depleted using CD3 microbeads. CD3^−^ CBMCs were stored as seed cells. When CAR-NK cells were manufactured, CD3^−^ CBMC seed cells were thawed and underwent two-cycle feed-cell stimulation and CAR transduction before infusion. FC represents fludarabine combined with cyclophosphamide.

We conducted a phase 1 trial of CD19-BBz CAR-NK cell therapy at the Second Affiliated Hospital, College of Medicine, Zhejiang University, Hangzhou, China, and assessed 11 individuals for eligibility, two of whom were excluded from the trial (Fig. 2a). Between July 26, 2022, and December 9, 2022, we enrolled the 9 above mentioned pre-screened patients with refractory LBCL in accordance with the protocol (Supplementary Information). These patients received the chemotherapy conditioning regimen; however, one of the nine individuals did not proceed with CAR-NK cell infusion due to active infectious disease and was excluded from all subsequent analyses.

The characteristics of the patients who received three weekly doses of CD19-BBz CAR-NK cells are shown in Table 1. The median age at CAR-NK infusion was 67 years, ranging from 48 to 73 years. All patients, including those with DLBCL (6 cases), transformed follicular lymphoma (tFL; 1 case), and mantle cell lymphoma (MCL; 1 case), had undergone heavy pretreatment, with a median of 5 previous lines (ranging from 3 to 8). Most patients (62.5%) presented with stage III or IV disease, 87.5% had extra-nodal involvement, 62.5% had elevated lactate dehydrogenase levels, and 87.5% had an intermediate or high international prognostic index score. It is worth noting that three patients had received prior CD19-CAR T-cell therapy (Supplementary Table 1). Upon enrollment, anti-lymphoma bridging therapy was not allowed, and post-remission treatment after the day 30 assessment was also prohibited unless relapse or disease progression was confirmed. The process and characteristics of the CD19-BBz CAR-NK cell products generated in our laboratory (Fig. 2b) are shown in Supplementary Table 2.

**Table 1:** Baseline characteristics

Characteristics	Number of patients, % (*n* = 8)
**Age (years)**	
˂60	1 (12.5%)
≥60	7 (87.5%)
**Sex**	
Male	5 (62.5%)
Female	3 (37.5%)
**History**	
DLBCL-GCB	1 (12.5%)
DLBCL-non-GCB	5 (62.5%)
MCL	1 (12.5%)
tFL	1 (12.5%)
**Disease stage at study entry**	
I or II	3 (37.5%)
III or IV	5 (62.5%)
**ECOG score**	
0	1(12.5%)
1	7 (87.5%)
**IPI score**	
0–1	1 (12.5%)
2–3	5 (62.5%)
4–5	2 (25%)
**LDH**	
<240	3 (37.5%)
≥240	5 (62.5%)
**Extra nodal organ involvement**	
0	1 (12.5%)
<2	2 (25%)
≥2	5 (62.5%)
**Number of previous lines of antineoplastic therapy**	
3	2 (25%)
4–8	6 (75%)
**Refractory or relapse**	
Primary refractory	6 (75%)
Relapse	2 (25%)
**Prior CD19 CAR T-cell therapy**	
CAR-T	3 (37.5%)
None	5 (62.5%)

GCB, germinal center B-cell-like subtype; ECOG, Eastern Cooperative Oncology Group; IPI, international prognostic index; LDH, lactate dehydrogenase.

### Favorable safety profile of CD19-BBz CAR-NK cell therapy

We evaluated adverse events of any grade attributable to any cause in all patients. It is worth noting that there were no dose-limiting toxicities (DLTs), and the maximum tolerated dose (MTD) was not reached in this study. Consistent with a previous report^24^, we observed no cases of CRS or neurotoxicity in any of the nine patients. Moreover, we did not observe the occurrence of GvHD, even though there were mismatches in human leukocyte antigen between patients and CAR-NK donors (Supplementary Table 1).

**Extended Data Fig. 2:**
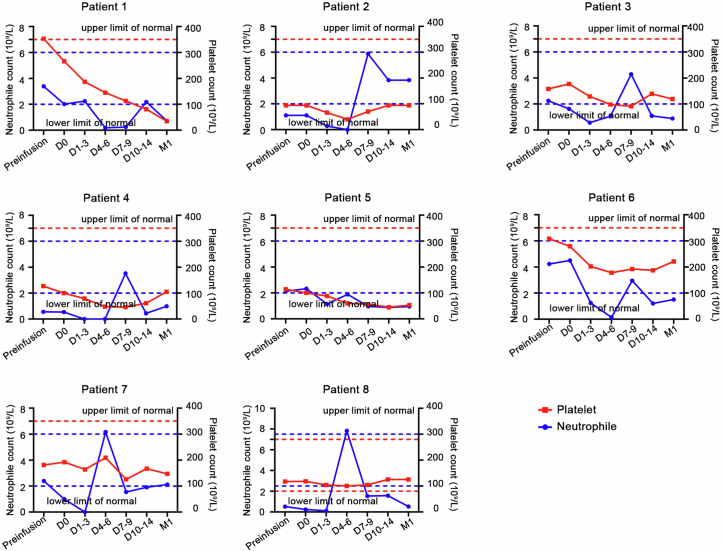
Hematopoietic toxicity of CAR-NK cell therapy. Absolute neutrophil counts and platelet counts are shown for patients receiving lymphodepletion chemotherapy at the indicated times post CAR-NK cell infusion (n = 8). Dashed red lines represent normal neutrophil counts, and dashed blue lines represent normal platelet counts. Source data

The most common adverse events related to CAR-NK therapy were hematologic toxicities, and all patients experienced grade 3–4 leukopenia. Grade 3 febrile neutropenia was observed in 3 (37.5%) patients, while 4 (50%) had grade 3 thrombocytopenia. It is worth noting that no patients experienced prolonged or delayed cytopenia, which is commonly observed in CAR T-cell therapies^26^ (Extended Data Fig. 2). In addition to hematological adverse events, a few patients experienced other grade 1 adverse events, such as constipation, nausea, and rash (Table 2).

**Table 2:** Adverse events among all 8 treated patients

Adverse events	Grade 0	Grade 1	Grade 2	Grade 3	Grade 4
CRS	8 (100%)	0	0	0	0
ICANS	8 (100%)	0	0	0	0
Leukopenia	0	0	0	1 (12.5%)	7 (87.5%)
Anemia	0	6 (75%)	2 (25%)	0	0
Granulocytopenia with fever	5 (62.5%)	0	0	3 (37.5%)	0
Thrombocytopenia	2 (25%)	2 (25%)	0	4 (50%)	0
Hypotension	7 (87.5%)	1 (12.5%)	0	0	0
Frequent micturition	8 (100%)	0	0	0	0
Rash	8 (100%)	0	0	0	0
Constipation	7 (87.5%)	1 (12.5%)	0	0	0
Hypofibrinogenemia	6 (75%)	2 (25%)	0	0	0
Nausea	7 (87.5%)	1 (12.5%)	0	0	0
Creatinine elevation	7 (87.5%)	1 (12.5%)	0	0	0

### Clinical outcomes of treatment with CD19-BBz CAR-NK cells

**Fig. 3:**
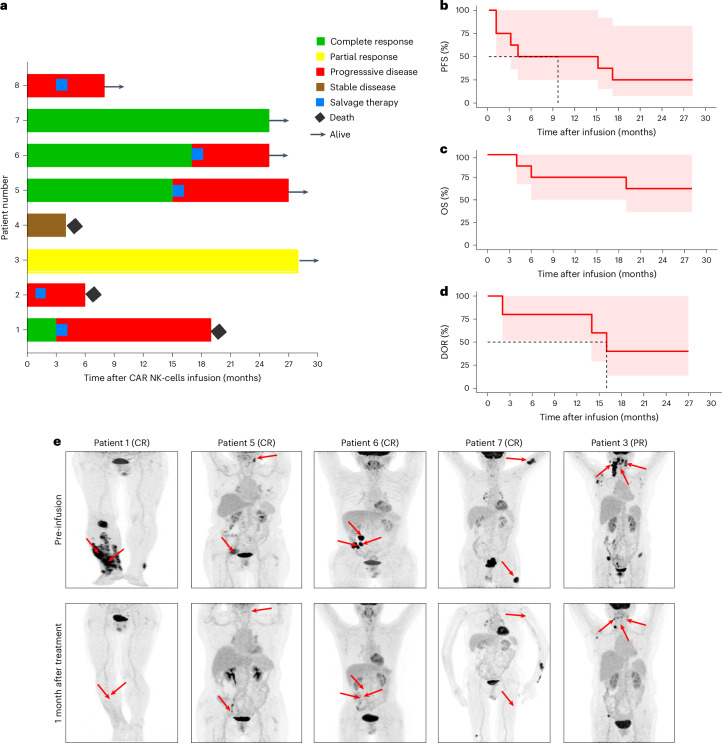
Efficacy profiles of CD19-BBz CAR-NK cell therapy. **a**, Swimmer plot (*n* = 8), in which each bar represents an individual patient. Responses evaluated at month 1 are designated by color. **b**–**d**, Kaplan–Meier analyses for PFS (*n* = 8) (**b**), overall survival (OS, *n* = 8) (**c**), and DOR (**d**) in the patients who achieved CR and partial response (*n* = 5). The red line represents Kaplan–Meier curves for PFS (**b**), OS (**c**), and DOR (**d**), and the dashed line indicates median PFS (**b**) and DOR (**d**), respectively. The red shading represents the 95% confidence interval. **e**, PET–CT scans of 5 patients who achieved a response before and after CAR-NK cell treatment. The red arrows indicate tumor locations. Source data

**Extended Data Fig. 3:**
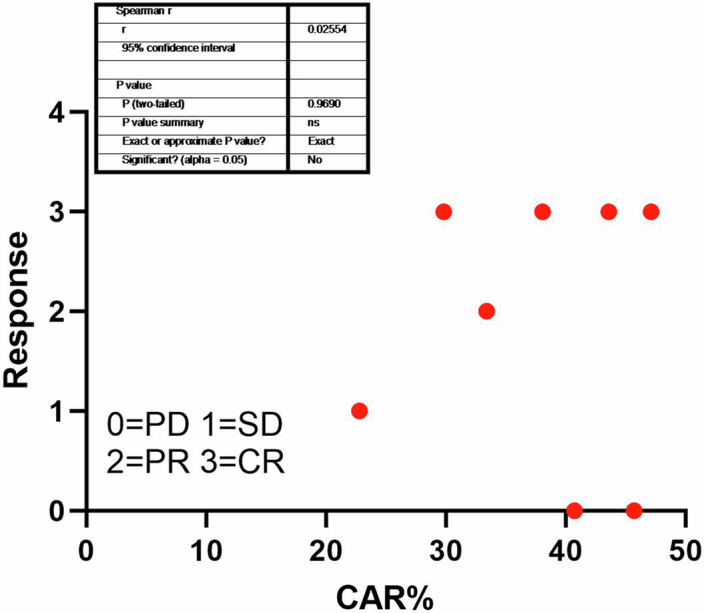
The association between the percentages of CAR+ cells of therapeutic product and clinical efficacy. The percentages of CAR^+^ cells in therapeutic products for 8 patients were detected by flow cytometry. The correlation between the percentages of CAR^+^ cells and responses were analyzed (n = 8, Spearman’s coefficient r = 0.02554, *P* = 0.9690). Spearman correlation analysis was used. Source data

The objective response was evaluated on day 30 after infusion in all patients. The overall response rate (ORR) was 62.5% (5 of 8), with 4 patients (50%) achieving a CR. Two patients exhibited evidence of progressive disease (PD) by day 30 (Fig. 3a). It is worth noting that neither CAR-NK cell dose nor percentages of CAR^+^ cells in the therapeutic products were associated with the clinical responses (Supplementary Table 2 and Extended Data Fig. 3). These are consistent with previous published results^24^.

The median follow-up time of this study was 25 months (ranging from 4 to 28 months). The median progression-free survival (PFS) was 9.5 months (Fig. 3b), and the median overall survival was not reached at the end of the study (Fig. 3c). Among the five patients who achieved a response, three unfortunately succumbed to PD, while the remaining two maintained durable responses (Fig. 3d). Pre- and post-treatment positron emission tomography–computed tomography (PET–CT) images of the five patients who responded, including those with CR and partial response, are shown in Fig. 3e. Together, these data indicate that multiple doses of CD19-BBz CAR-NK cell therapy produced potent and durable antitumor responses.

### Pharmacodynamics of CD19-BBz CAR-NK

**Fig. 4:**
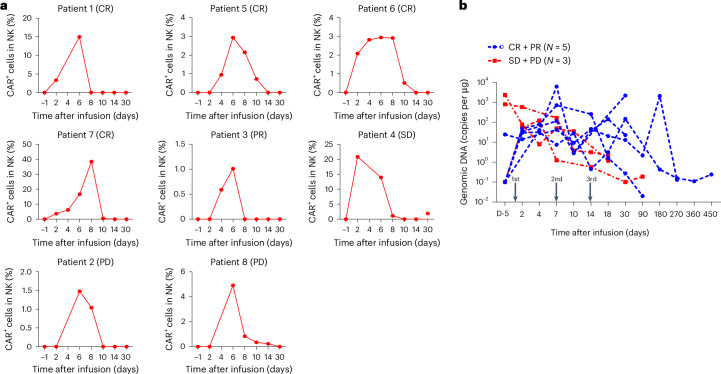
Proliferation and persistence of CD19-BBz CAR-NK cells. **a**, CAR^+^ NK cell counts in peripheral blood of 8 patients after infusions were assessed by flow cytometry. CAR^+^ NK cells were identified as CD56^+^CAR^+^ cells in CD3^−^CD56^+^ NK cells gated on the CD45^+^ lymphocyte population. The gate strategies are listed in Supplementary Fig. 1c. Myeloid cells were excluded by gating on the CD33 and CD14 positive cells. CD56^+^CAR^+^ cells were gated in CD3^−^CD56^+^ NK cells from the CD45^+^CD33/CD14^−^ lymphocyte population. **b**, Kinetics of genetically modified NK cells in peripheral blood were determined by ddPCR. The arrow indicates times of CAR-NK cell infusion. Source data

**Extended Data Fig. 4:**
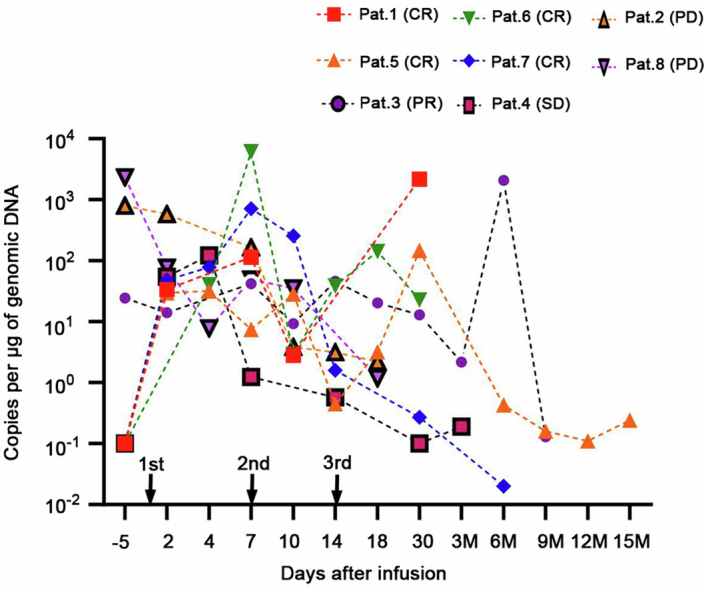
Kinetics of genetically modified NK cells in peripheral blood of patients were determined by ddPCR. The times of CAR-NK cell infusion are marked by black arrows (n = 8). Source data

Early expansion and persistence of CAR T cells may be crucial for achieving durable responses in patients with B cell lymphoma^2,27,28^ Therefore, we assessed in vivo expansion of CD19-BBz CAR-NK cells by flow cytometry in the blood of all patients after infusion. The patients exhibited the highest numbers at 4 to 7 days after CAR-NK treatment, although the counts of CAR-NK cells varied (Fig. 4a). CAR copies were detected by digital droplet polymerase chain reaction (ddPCR) analysis. CAR-NK cell proliferation and longer persistence were observed in patients having a positive response. Among them, one patient who achieved CR showed a low-level, long-term persistence of CAR-NK cells, with detectable CAR copies in the peripheral blood at 15 months, and another patient with partial response had high level of CAR DNA copies on day 180. These results are consistent with previous clinical findings^23^. However, CAR copies were not detected 2 weeks after infusion in those patients with no response (Fig. 4b and Extended Data Fig. 4).

### Serum cytokine profile of CD19-BBz CAR-NK

**Extended Data Fig. 5:**
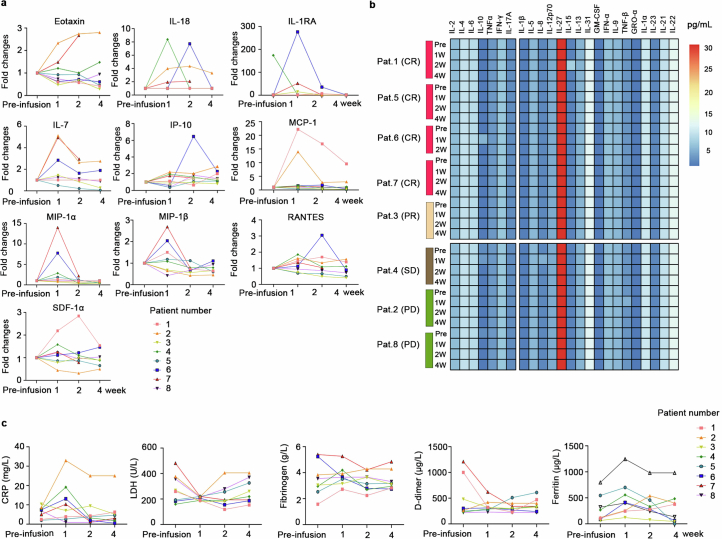
The changes of the cytokine and marker of inflammation and coagulopathy in serums obtained from the patients. **a**, Serum kinetics of cytokines of eotaxin, IL-18, IL-1RA, IL-7, IP-10, MCP-1, MIP1α, MIP1β, RANTES, and SDF-1ɑ in 8 patients who received infusions with CAR-NK cells. **b**, Heatmap of a panel of other cytokines detected in the serum from 8 patients at pre- or post-infusion of CAR-NK cells. **c**, Serum levels of CRP, LDH, Fibrinogen, D-dimer, and Ferritin in 8 patients before and after CAR-NK cell infusion. Source data

CRS is characterized by inflammatory responses caused by multiple cytokines and immune modulators, such as IL-6 and interferon-γ (IFNγ), produced by activated immune cells^7,13,29^. The serum cytokine profile of CD19-BBz CAR-NK cells differed from that of anti-CD19 CAR T-cell therapy, with slightly elevated levels of eotaxin, IL-18, IL-1RA, IL-7, IFNγ-inducible protein-10, monocyte chemoattractant protein-1, macrophage inflammatory protein 1 alpha, MIP1β, C-C Motif Chemokine Ligand 5, and stromal-derived-factor-1 alpha in a few patients (Extended Data Fig. 5a). However, 24 of 34 cytokines remained at basal levels within 4 weeks after CAR-NK infusion (Extended Data Fig. 5b). No notable changes were observed in markers of inflammation and coagulopathy including C-reactive protein, ferritin, and D-dimer (Extended Data Fig. 5c).

### Transcriptome analysis of infusion products and peripheral blood mononuclear cells

**Fig. 5:**
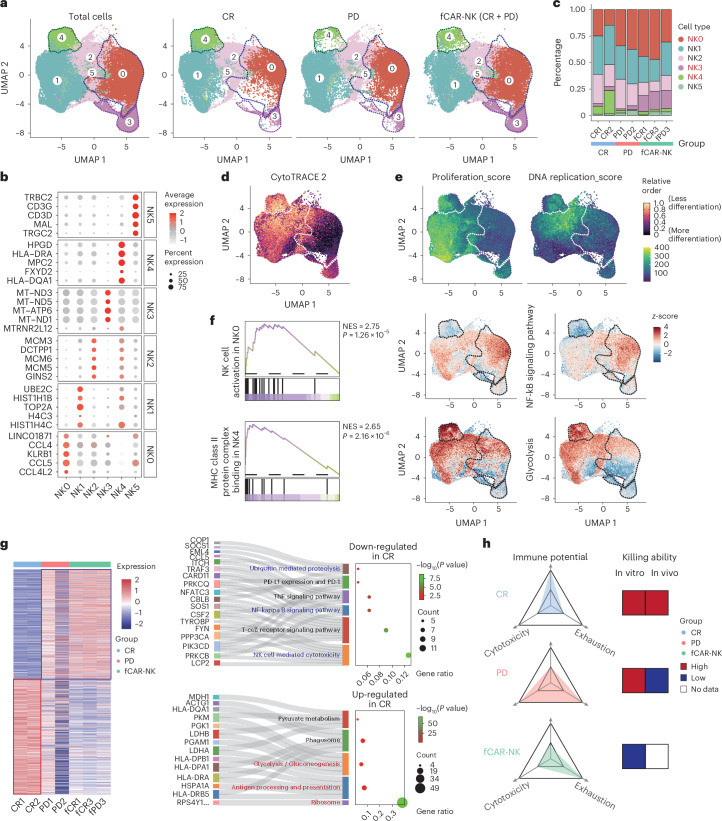
Single-cell transcriptome of CD19-BBz CAR-NK products. **a**, UMAP of 89,658 NK cells from 7 CAR-NK products, showing the formation of 6 subclusters shown in different colors, of which 2 products were linked to CR, 2 products were linked to PD, and 3 products (fCAR-NK) were analyzed after cryopreservation and revival. **b**, Top five DEGs in each identified subcluster. **c**, The fractions of each subcluster in each sample. **d**, UMAP showing the cell differentiation potential of each subcluster calculated using the CytoTRACE v2 algorithm. **e**, Expression distribution of gene sets related to cell proliferation and DNA replication using the UCell algorithm. The dashed circles in **a**, **d** and **e** highlight immune cell subtypes exhibiting significant differences in proportions between groups. **f**, Gene set enrichment analysis of DEGs in cluster NK0 and NK4 and mapping to UMAP for illustration (UCell scores were standardized by *z*-score). *P* value was calculated from empirical phenotype-based permutation test. NES, normalized enrichment score. **g**, Heat map of DEGs between CR, PD, and fCAR-NK groups, and mulberry plots showing their KEGG enrichment terms. *P* value was calculated from Fisher exact test. **h**, Evaluation models related to in vitro and in vivo efficacy. Source data

**Extended Data Fig. 6:**
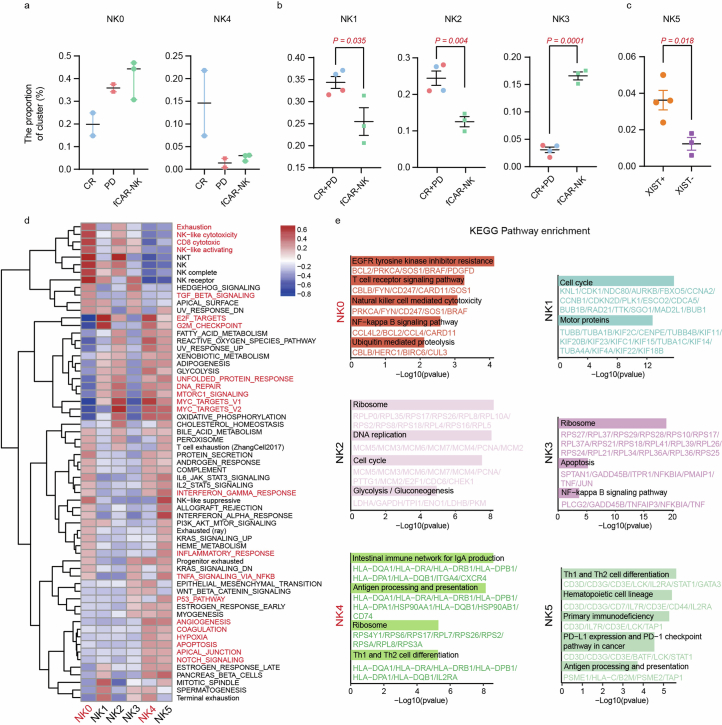
Functional analysis of various clusters of CD19-BBz CAR-NK products. **a**, Scatter plot (showing median and extremum) illustrating intergroup differences in subclusters (n = 2 for CR, 2 for PD, 3 for fCAR-NK). **b**, Differences were compared between CR + PD (n = 4) and fCAR-NK (n = 3). Data were illustrated as mean ± SEM. A two-sided student’s t-test was used. *P* = 0.035 (NK1), *P* = 0.004 (NK2), *P* = 0.0001 (NK3). **c**, Scatter plot (showing mean ± SEM) illustrating differences between CAR-NK products derived from male (XIST-, n = 3) and female (XIST + , n = 4). P values were calculated using a two-sided student t-test. *P* = 0.018. **d**, GSVA analysis based on gene expression levels of each subcluster, data source from Hallmarker (n = 2 for CR, 2 for PD, and 3 for fCAR-NK). **e**, Bar plot of KEGG enrichment analysis of DEGs of each subcluster (n = 2 for CR, 2 for PD, and 3 for fCAR-NK). Source data

We next performed scRNA-seq on freshly collected CAR-NK products administered to two patients who achieved CR after CAR-NK treatment and two patients with PD, respectively. Furthermore, we also included three cryopreserved and revived CAR-NK products (two linked to CR and one linked to PD) for scRNA-seq analyses. A total of six NK cell subclusters were identified, each showing distinct signature genes (Fig. 5a,b). The CAR-NK products for patients with CR had a higher proportion of cells featured in the NK4 subcluster, whereas those linked to PD contained more cells belonging to the NK0 subcluster (Fig. 5c and Extended Data Fig. 6a). Interestingly, after cryopreservation, the proportion of NK3 subcluster with high expression of mitochondrial related genes significantly increased (Fig. 5b,c and Extended Data Fig. 6b). By scoring differentiation potentials, we found that the NK4 subcluster was the least differentiated, followed by NK1 and NK2 subclusters, and the NK0 subcluster is most differentiated/mature (Fig. 5d). Heightened expression of cell proliferation related genes in NK1 and DNA replication genes in NK2 suggests that they are in different proliferation states, while NK4 possesses both of these characteristics simultaneously, indicative of a proliferative state (Fig. 5e). After cryopreservation, despite being revived for several days, the proportions of NK1, 2, and 4 all somewhat decreased, while NK3 subcluster dramatically increased (Fig. 5c and Extended Data Fig. 6a,b). We further conducted gene and pathway enrichment analysis using multiple algorithms and demonstrated that NK4 had low expressions of NF-κB signaling genes and elevated expressions of MHC class II genes, while NK0 had the opposite trend (Fig. 5f and Extended Data Fig. 6d,e). Moreover, the NK5 subcluster expresses T-cell-related genes, and its proportion is significantly correlated with gender, but not with therapeutic efficacy (Fig. 5b,c and Extended Data Fig. 6c–e).

**Extended Data Fig. 7:**
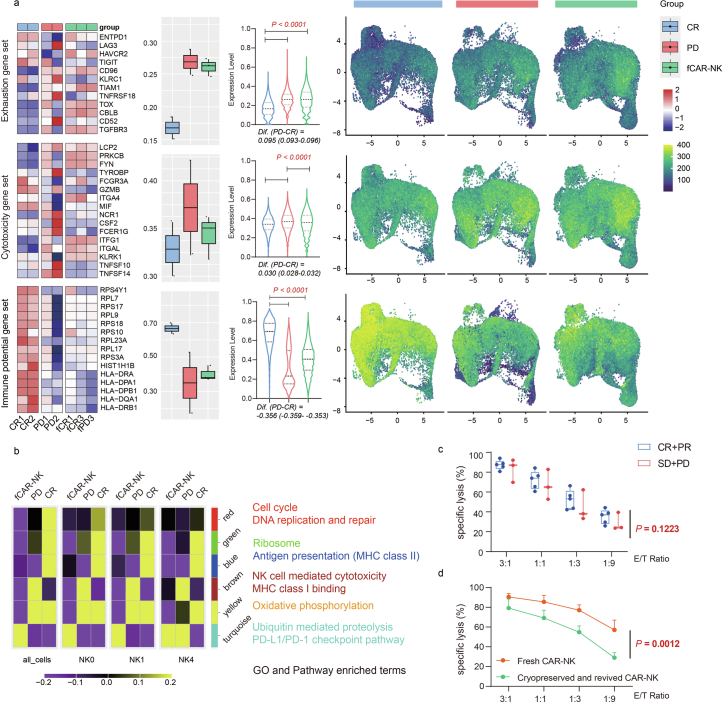
Functional analysis between CD19-BBz CAR-NK product groups. **a**, Heatmap of representative “exhaustion,” “cytotoxicity,” and “immune potential”-related gene sets across CR, PD, and fCAR-NK group, boxplots of these three gene sets mean scores of each product (centre of box mean median, bounds of box means upper and lower quartiles, whisker means maximum and minimum, n = 2 for CR, 2 for PD, 3 for fCAR-NK), and violin plot of these gene set scores of each single cell (showing median, quartiles and extremum, n = 23644 cells from 2 CR samples, 27826 cells from 2 PD samples, 38188 cells from 3 fCAR-NK samples) and visualization on UMAP. A two-sided student’s t-test was used for every two groups. *P* < 0.0001. **b**, Module-trait relationship based on WGCNA analysis, and gene Ontology (GO) and KEGG pathway analysis of each module from WGCNA. **c**, The cytotoxic activities of CD19-BBz CAR-NK cells for CR + PR patients (n = 5) compared to those from SD + PD patients (n = 3) on Raji cells at the indicated effector-to-target (E/T) ratios by measured using Luciferase-based cytotoxicity assays. Data are expressed as median and quartiles (each dot plot represents 3 independent experiments and 4 technical repeats for each CAR-NK product). Two-way ANOVA (Bonferroni posttest) was used between groups. *P* = 0.1223. **d**, Cytotoxic activities of fresh CAR-NK cells compared to cryopreserved CAR-NK cells on Raji cells at the indicated effector-to-target (E/T) ratios as measured by Luciferase-based cytotoxicity assays. Data represent 3 independent experiments and 4 technical repeats, and are expressed as mean ± SEM. Two-way ANOVA (Bonferroni posttest) was used. *P* = 0.0012. Representative data from 1 independent experiment with 4 technical replicates. Source data

From differentially expressed genes (DEGs) between CAR-NK products that are linked to CR versus PD (Fig. 5g), Kyoto Encyclopedia of Genes and Genomes (KEGG) enrichment analysis indicated that ribosomes, antigen presentation, and glycolysis signals were enriched in CR-linked products, which we considered as the “immune potential” gene set. Cytotoxic pathways and ubiquitination processes were enriched in PD-linked products (Fig. 5g), suggesting that both premature activation and exhaustion states coexist in CAR-NK products that are linked to PD. We therefore collected gene sets related to exhaustion, cytotoxicity, and immune potential in DEGs (Extended Data Fig. 7a) and found that both high exhaustion and cytotoxicity were featured in the PD group, with exhaustion being particularly significant (Extended Data Fig. 7a). Through in vitro cytotoxicity assays, PD-linked cell products happened to have slightly higher tumor killing activities compared to CR-linked NK cells (Extended Data Fig. 7c), suggesting in vitro tumor killing activities could not directly predict treatment efficacy, further supporting the notion that high exhaustion level is perhaps one of the main reasons why such CAR-NK products are ineffective (Extended Data Fig. 7a,c). The cryopreserved/frozen CAR-NK (fCAR-NK) group does have lower cytotoxicity and immune potential based on transcriptomic analyses, and in vitro tumor-killing assays also showed their reduced cytotoxic abilities (Extended Data Fig. 7d). Weighted gene co-expression network analyses also supported the notion that cryopreservation may substantially and permanently dampen the efficacy of CAR-NK products (Extended Data Fig. 7b). Together, we established a transcriptome-based model to evaluate CAR-NK products, which may predict their clinical efficacy by assessing levels of exhaustion, cytotoxicity, and immune potential (Fig. 5h).

**Fig. 6:**
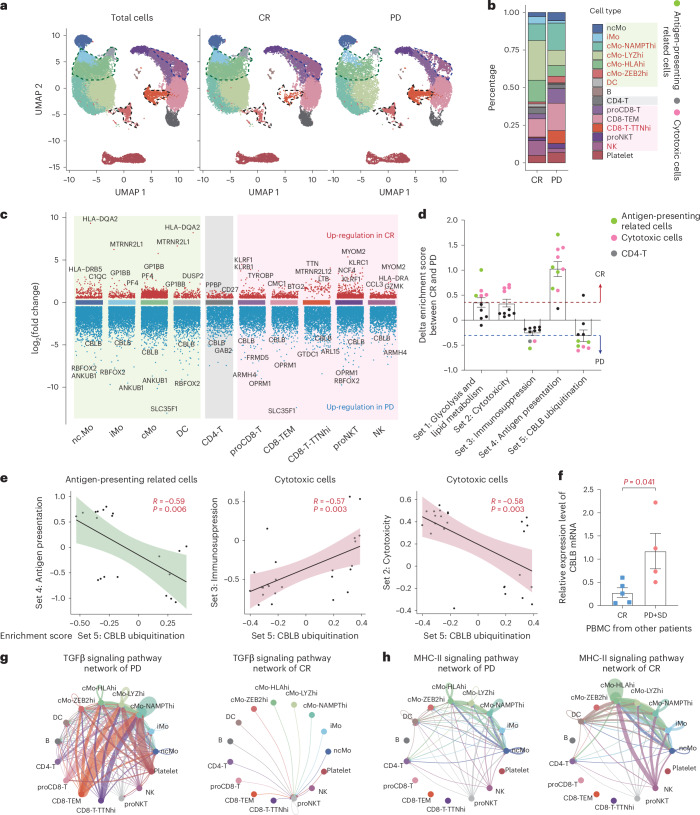
Single-cell transcriptomic landscape of peripheral immune cells. **a**, UMAP visualization of transcriptomic profiles of 51,068 cells in PBMC samples obtained from 5 patients (3 from CR and 2 from PD). Fifteen distinct clusters were identified. The dashed circles highlight immune cell subtypes exhibiting significant differences in proportions between groups. **b**, Distribution of each cluster between the CR and PD groups. **c**, Volcano diagram of DEGs between CR and PD groups in each subcluster of antigen-presenting related cells, CD4-T cells, and cytotoxic cells. **d**, GSVA score differences (CR versus PD, median of delta GSVA score, mean ± s.e.m.) across five gene sets (above the red dashed line indicates enrichment in CR, while below the blue dashed line indicates enrichment in PD). Dot plots represent ten subclusters which were classified into three cell types. **e**, Pearson correlation analyses of “*CBLB*-ubiquitination” gene set with antigen presentation, immunosuppression, and cytotoxicity gene sets in two different cell types. Each scatter point represents GSVA score of a subcluster of the same cell types from five samples. Correlation coefficient *P* value was calculated. **f**, Quantitative reverse transcription PCR validation of CBLB mRNA expression in patients’ PBMCs. Data are expressed as mean ± s.e.m. (*n* = 5 versus 4). Two-sided Student’s *t*-test was used. **g**,**h**, Analysis of communication among immune cells in patients with CR and patients with PD regarding *TGFβ* signaling (**g**) and MHC-II signaling (**h**). Source data

**Extended Data Fig. 8:**
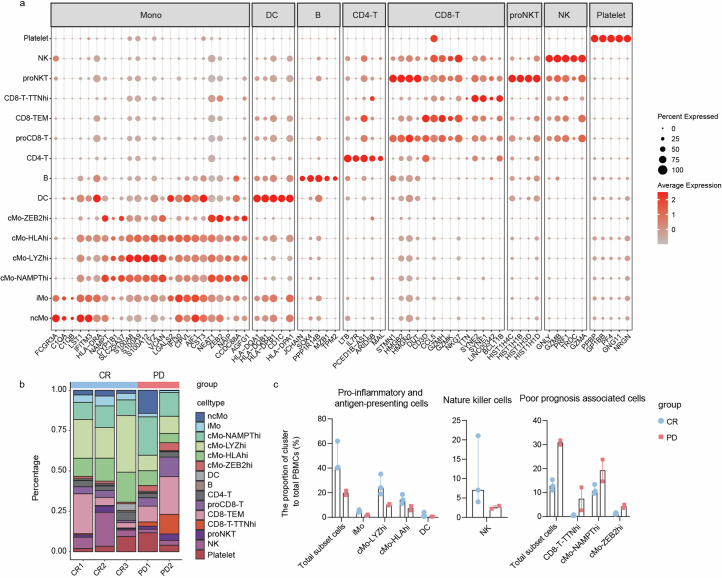
Differences of PBMCs clusters between groups. **a**, Bubble plot indicating the expression of marker genes across all subclusters. **b**, Bars chart showing the fractions of each subcluster in each sample. **c**, Scatter plot displaying cell subpopulations with stable differences between groups (data are presented as median and extremum). n = 3 for CR, 2 for PD in a, b and c. Source data

**Extended Data Fig. 9:**
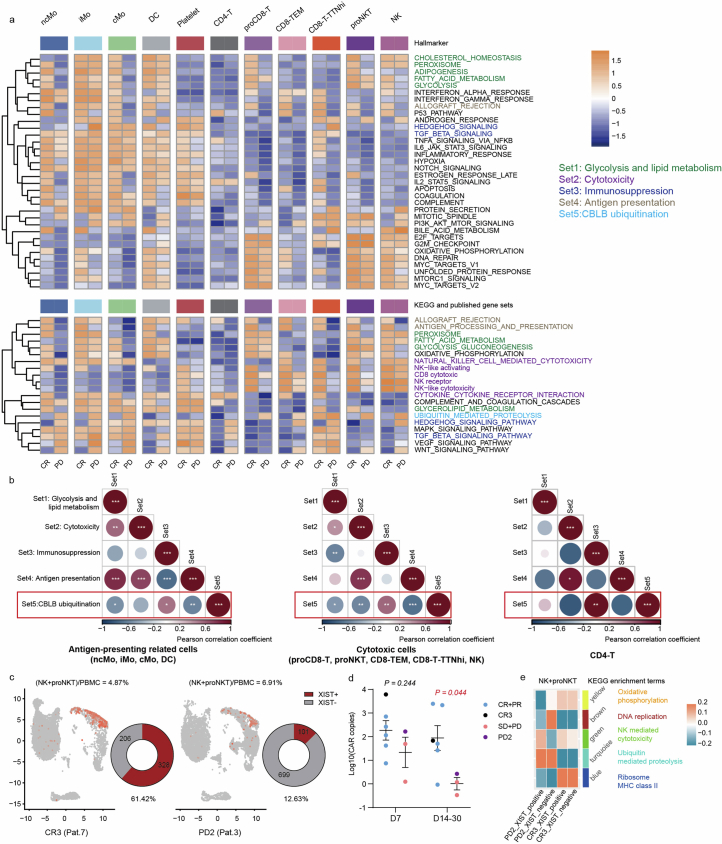
Functional analysis between groups. **a**, GSVA analysis based on gene expression levels of CR and PD, data source from Hallmarker (above) and KEGG (below). The pathways with differences between groups are divided into five gene sets based on biological functions (n = 3 for CR,2 for PD). **b**, Pearson correlation heatmap of five gene sets in antigen-presenting related cells, CD4 + T cells, and cytotoxic cells (n = 3 for CR,2 for PD). **c**, Distribution of XIST in two male patients’ PBMCs (CR3 and PD2), and the proportion of injected XIST+ cells to total NK plus Pro. NKT cells. **d**, Log copy numbers of CAR gene at 7 days (n = 6) and 14-30 days (n = 3) after administration. Data are expressed as mean ± SEM. A two-sided student’s t-test was used for each condition. *P* = 0.244 (Day 7), *P* = 0.044 (Day 14-30). **e**, Module-trait relationship based on WGCNA analysis of sc-RNA-seq of XIST+ (donor cells) and XIST- (recipient cells) in NK plus Pro. NKT populations of CR3 and PD2 samples, gene Ontology (GO), and KEGG pathway analysis of each module are presented. Source data

To assess the impact of CAR-NK treatment on peripheral immune cells in patients, we analyzed peripheral blood mononuclear cell (PBMC) profiles from 3 patients with CR and 2 patients with PD 1 week after administration. Among regenerated T lymphocytes, CD8-T-cell populations were the predominate populations, and CD4-T cells represented a minor population (Fig. 6a,b). The top defining markers for each cluster were visualized (Extended Data Fig. 8a). There were marked differences in the proportions of antigen-presenting related cells and cytotoxic cells between patients with CR and patients with PD (Fig. 6a,b and Extended Data Fig. 8b,c). To comprehensively evaluate potential obstacles for incomplete tumor remission, we divided sequenced PBMCs into three categories—(1) antigen-presenting (including all kinds of monocytes and dendritic cells), (2) cytotoxic (including all kinds of CD8-T and NK cells), and (3) CD4-T—and analyzed their inter-CR-PD group DEGs, as well as gene set variation analysis (GSVA) (Fig. 6c and Extended Data Fig. 9a). In the enriched differential pathways, we summarized five sets, including “glycolysis and lipid metabolism,” “cytotoxicity,” “immunosuppression,” “antigen presentation,” and “ubiquitination” (Extended Data Fig. 9a). Based on the median (to avoid individual bias as much as possible) of mean delta scores between CR and PD, it was found that “antigen-presenting” cells and “cytotoxic” cells were mostly involved in generating the aforementioned 5 categories of gene expression differences between CR and PD, whereas CD4-T cells only played a role in the immunosuppressive perspective (Fig. 6d). Through cell type-specific correlation analyses, ubiquitination seems to have a broader impact, that is, *CBLB*-ubiquitination is significantly correlated with not only antigen presentation but also cytotoxicity and immunosuppression, whereas glycolysis and lipid metabolism were not significantly altered in CD4- and the majority of CD8-T cells (Fig. 6e and Extended Data Fig. 9a,b). To confirm the role of ubiquitination on efficacy, we collected PBMCs from additional patients with CR and patients with PD and found that the ubiquitin E3 ligase, *CBLB*, was expressed at significantly lower levels in PBMCs of patients with CR versus patients with PD (Fig. 6f).

We also managed to detect residual CAR-NK products in vivo via PBMC scRNA-seq. From patients CR3 and PD2, both male, who happened to receive female CAR-NK products, the gender differences allowed us to distinguish donor cells from recipients’ cells through *XIST* gene analysis. As expected, the only *XIST*^+^ cells are distributed in the subpopulations of proliferative NKT and NK cells (Extended Data Fig. 9c). It appeared that the number and proportion of CAR-NK products in the patient with CR were higher than those in PD (Extended Data Fig. 9c,d). Although only one pair of samples were available for study, this observation suggests that features of each recipient’s immune system, particularly interactions among CAR-NK products and the various recipient immune cell types, might influence CAR-NK treatment efficacy (Extended Data Fig. 9e). In cell–cell ligand–receptor communication (Cell Chat) analysis, we found that the *TGFβ* signaling, typically associated with immune suppression and exhaustion^30,31^, was markedly enhanced in immune systems of patients with PD (Fig. 6g), suggesting that immune systems of patients with PD are in a suppressed state, likely leading to poor treatment outcome. Nonetheless, the antigen presentation signals between NK cells and monocytes are substantially enhanced in patients with CR (Fig. 6h). This highlights the importance of host immune states influencing treatment outcome. As *CBLB*-mediated ubiquitination is positively correlated with immune suppression (*TGFβ* signaling) and negatively correlated with MHC-II related antigen presentation and cytotoxicity, we speculate that *CBLB*-mediated ubiquitination could be an important target, inhibition of which could be key for the sustained efficacy of CAR-NK.

## Discussion

Our results suggest that CD19-BBz CAR-NK cells were feasible and therapeutically safe, with low toxicity and efficacy with sustained remission. Numerous preclinical studies have demonstrated the efficacy of CD19-specific CAR-NK cells using various CAR molecules with different stimulatory domains such as CD28-CD3ζ or 4-1BB-CD3ζ domains^32–36^. The choice of costimulatory domains in CAR molecules impacts T-cell differentiation pathways and metabolic cycles^37^. The CD28 domain tends to promote the short-lived T-effector-memory phenotype and enforce T-cell activation and rapid tumor killing, yet leads to T-cell exhaustion, which limits enduring effect. By contrast, CAR T cells expressing 4-1BB showed enrichment of longer-lived, self-renewing central-memory phenotype, coupled with enhanced persistence^38,39^. It is worth noting that 4-1BB is also naturally expressed in NK cells, promoting their effector functions and increasing antibody-dependent cell-mediated cytotoxicity^25,40,41^.

Viral gene delivery to NK cells has been challenging and less effective than in T cells^42^. Despite the success of retroviral transduction in a clinical trial of CAR-NK therapy, retroviral vectors are inherently limited by insertional mutagenesis and negative impacts on the viability of NK cells^19,43^. By contrast, lentiviral vectors show lower genotoxicity^44^. Our study demonstrates the therapeutic efficacy of CD19-BBz CAR-NK cells established by BaEV-LV transduction. CD19-BBz CAR-NK cells mediated rapid and nearly complete tumor depletion, consistent with their higher potency in vitro. Mechanistically, the enhanced persistence and infiltration abilities of CD19-BBz CAR-NK cells might contribute to their stronger antitumor efficacy in vivo.

Based on our preclinical results, we conducted a phase 1 trial with the objective of evaluating the safety and feasibility of administrating multiple doses of CD19-BBz CAR-NK cells to patients with R/R LBCL. As expected, treatment was safe with no severe toxicities, including CRS, ICANS, and GvDH, which aligns with two recently published studies^23,24,45^. Moreover, no DLT was observed at any dose levels, and the MTD was therefore not reached. The most common grade 3 or worse adverse events were bone marrow suppression related to lymphodepletion chemotherapy.

This study enrolled 8 heavily pretreated patients. Among them, 6 (75%) patients met the criteria for refractory aggressive LBCL. In the SCHOLAR-1 retrospective analysis, the ORR was 26% to the next line of treatment, and the median overall survival was 6.3 months among patients with refractory DLBCL^46^. Although our study cannot be directly compared with pivotal clinical studies of CD19-CAR T cells, the efficacy of CD19-BBz CAR-NK cells was comparable to that of CAR T-cell therapy^5^. However, the small sample size of this trial urges its validation in larger cohorts. It is worth noting that durable efficacy of CD19-BBz CAR-NK cells was confirmed, even though patients with a response did not receive any additional antitumor treatment during follow-up.

The mechanisms underlying the high efficacy of CD19-BBz CAR-NK cells are not yet clear. For majority of cytotoxic cells including NK cells, multiple cell contacts are needed for effective killing^47^. During our trial, several studies have demonstrated that high-dose and multiple CAR-NK cell infusions can improve antitumor capacity and durability of clinical response^45,48,49^. Our work provided a framework for a rational CAR-NK cell dosing strategy and suggests that high doses and repeated administration of CAR-NK products are necessary for achieving better antitumor efficacy.

**Extended Data Fig. 10:**
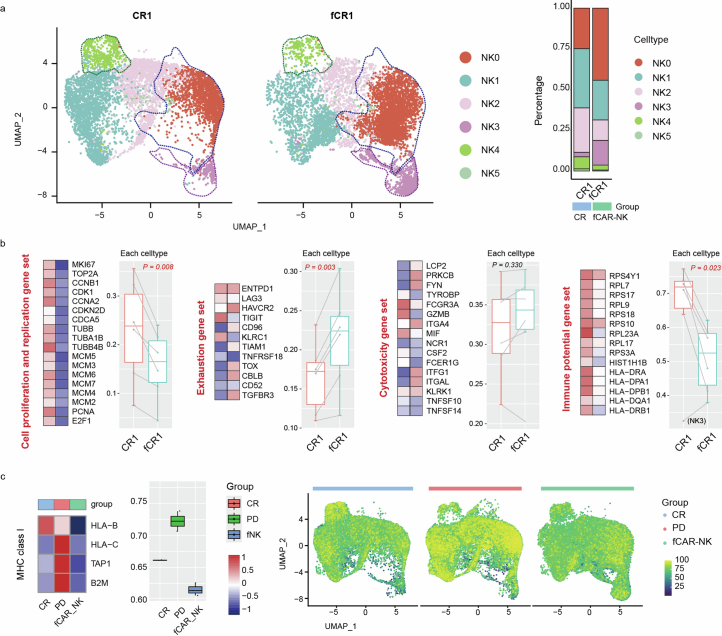
The differences before and after cryopreservation of the same CAR-NK product revealed by sc-RNA-seq. **a**, UMAP, and subcluster ratio of CR1 and fCR1. **b**, Changes of 6 subclusters in various functional gene sets before and after cryopreservation. A two-sided paired t-test was used (n = 6). *P* = 0.008, 0.003, 0.33, and 0.023, respectively. **c**, UCell scores of MHC class I related genes among groups (n = 2 for CR, 2 for PD,3 for fCAR-NK). All data (b-c) were shown as median, quartiles, and extremum (centre of box means median, bounds of box means upper and lower quartiles, whisker means maximum and minimum). Source data

It is well known that the success of adoptive NK-cell therapy has been limited by their short lifespan and rejection by host T cells^50^. Consistent with a previous report^23^, we also demonstrated long-term persistence and in vivo expansion of CAR-NK cells. Apart from the persistence of CAR-NK cells, some other critical factors could affect therapeutic efficacy. For instance, a recent study using samples from CD19-28z CAR-NK cell-treated patients demonstrated that loss of metabolic fitness could influence the ability of CAR-NK cells to eliminate tumors^49^. In this study, CD19-BBz CAR-NK cell therapy shows promise for R/R LBCL; however, some patients either did not respond or relapsed after an initial response to this treatment. Therefore, we performed scRNA-seq to explore cellular features of CAR-NK cell infusion products associated with response and compared the transcriptional profiles of PBMCs from responders and non-responders. We observed that the MHC class II pathway was active in the CR group recipients and CAR-NK products. The NK subtype that highly expressed this pathway also highly expressed cell proliferation and ribosome-related genes, which could be considered as a subtype with greater immune potential^51,52^. Although we did not find any difference in the in vitro cytotoxicity of CAR-NK products between the CR and PD groups, due to differences in immune potential, patients who achieved CR after CAR-NK treatment not only maintained active MHC class II pathways but also obtained high levels of cytotoxicity and low levels of immune suppression. Therefore, in our data, we emphasize the importance of the immune potential of CAR-NK products for clinical therapeutic efficacy. We considered the limitations of small sample sizes and therefore added a set of cytotoxicity experiments and transcriptome analysis on fCAR-NK. We found that cryopreservation of cell products significantly decreased cell proliferation and immune potential while increasing cell exhaustion. To be more precise, we extracted an additional pair of the same sample before and after cryopreservation for comparison and found that, consistent with the population-based result, the proliferation and immune potential of CAR-NK products after cryopreservation decreased, while the level of immune suppression significantly increased (Extended Data Fig. 10a,b). In addition, we found that some MHC class I genes are highly expressed in CAR-NK products of PD group (Extended Data Fig. 10c), which may lead to easier exclusion as allogeneic cells. Through transcriptomic functional module analysis and cell communication analysis of PBMCs from patients with different clinical outcomes, we found that in patients with PD, immune suppression levels (featured by *TGFβ* signaling) were markedly elevated, while cytotoxicity and antigen presentation activity were reduced. We also discovered that *CBLB*-mediated ubiquitination may be involved in multiple important antitumor biological processes including antigen presentation, cytotoxicity, and exhaustion. *CBLB* has been found to be an important factor in NK’s inability to activate CD8 T and NK cells^53,54^ and may also generate resistance to CAR-NK’s therapeutic effect.

Because of the dose-escalation design as this study is non-comparative, the small cohort size, and the inclusion of several B-cell malignancy subtypes, the results are exploratory and need to be confirmed in larger cohorts. It is worth noting that a phase 2 dose-expansion study evaluating the recommended dose of [4 × 10^6^ kg^−1^] CAR-NK cells (as determined in this phase 1 trial) is currently ongoing to further assess efficacy and safety.

In summary, this study demonstrates the effectiveness of CD19-BBz CAR-NK cell therapy with repetitive administration for the treatment of R/R LBCL. It is worth noting that this approach does not result in severely toxic conditions such as CRS, ICANS, or GvHD, making it a safe and potent option for allogeneic CD19-CAR-NK cell therapy.

## Methods

### Cell lines

Raji (TCHu 44) and JeKo-1 (TCHu194) cells were obtained from the Cell Bank of the Chinese Academy of Sciences. HEK293T (CRL-3216), NIH/3T3 (CRL-1658), and K562 (CCL-243-ATC) were purchased from the American Type Culture Collection. Cell lines were authenticated by short-tandem-repeat analysis. The K562 feeder cells expressing 4-1BB ligand and membrane-bound IL-21 were generated by lentiviral transduction and followed by irradiation. A Raji-luc or JeKo-1-luc cell line stably expressing EGFP fused firefly luciferase was established by lentiviral infection and sorted with a BD FACSAria II Cell Sorter.

### CAR vector construct and lentiviral production

The CD19-BBz CAR was constructed by synthesized DNA fragments (Genscript) encoding the following components: a CD19 binding scFv (FMC63; GenBank, HM852952.1), CD8a stalk and transmembrane segments (amino acids (aa) 138–206), 4-1BB intracellular domain (aa 214–255), CD3ζ intracellular domain (aa 52–164), a T2A self-cleaving peptide, and IL-15. A claudin 18.2-BBz CAR was also constructed, which was used as a non-relevant control. The construct was cloned via In-Fusion cloning (Takara Bio) into a third-generation lentiviral backbone plasmid kept in house, and further verified by Sanger sequencing (GENEWIZ). Lentiviral particles were generated as previously described^55^ by transient transfection of HEK293T cells with the CD19-BBz CAR encoding lentiviral vectors, pLP1 (Thermo Fisher) plasmid encoding Gag-Pol, pLP2 (Thermo Fisher) plasmid with Rev gene, and a plasmid encoding the BaEV envelope protein. Lentivirus-containing supernatants were collected at 48 h after transfection, filtered through 0.45 µm filters, concentrated by ultracentrifuge, and snap frozen for later transduction of NK cells.

### Cytotoxicity assays

Luciferase-based cytotoxicity assays were performed to determine the cytotoxicity of CD19-BBz CAR-NK against lymphoma cells. In brief, 1 × 10^4^ Raji-luc or JeKo-1-luc were co-cultured with effectors (NK or CAR-NK cells) at the indicated effector-to-target (*E*/*T*) ratio for 4 h in a 96-well round-bottom plate. About 0.5 mM d-luciferin potassium salt (PerkinElmer) was added to each well, and the resulting luminescence was analyzed on a microplate reader (Thermo) in the model of luminometric measurement as 1,000 ms for each well detection. Target-only cells defined as minimum killing ability (*K*_min_). and target-only cells plus 2.5% Triton-X 100 defined as maximum killing ability (*K*_max_). *K*_max_ and *K*_min_ were used to determine the assay range. Percent specific lysis was calculated as: ((*K*_sample_ − *K*_min_)/(*K*_max_ − *K*_min_)) × 100%.

### Confocal microscope

Coverslips were prepared using poly-l-lysine (Sigma-Aldrich) overnight at 4 °C. Then, a mixture of Raji-luc with CAR-NK cells (2:1) were plated onto the coverslips for 1 h. Next, the cells were fixed with 4% paraformaldehyde for 10 min at room temperature. The Alexa Fluor 647 conjugated anti-mouse FMC63 scFv primary antibody (BioSwan Lab) were incubated (1:500 dilution) at 4 °C overnight. Coverslips were washed with PBS buffer and mounted using ProLong Gold Antifade Mountant (Thermo Fisher). Imaging was performed on a Zeiss LSM 880 confocal laser scanning microscope (Carl Zeiss AG).

### Continuous cytotoxicity assay

The prolonged in vitro cytotoxic capabilities of the CAR-NK cells were assessed using NIH/3T3 cells engineered to express CD19, at 1:1 or 1:3 *E*/*T* ratio. Cytotoxicity was evaluated using a label-free real-time cell analyzer system (Agilent Biosciences). NIH/3T3-CD19 cells were initially plated at a density of 1 × 10^4^ cells per well within the real-time cell analyzer unit for culture. After 72 h incubation, either control NK cells or CAR-NK cells were subsequently introduced. Impedance signals were recorded at 5 min intervals.

### IL-15 secretion analysis

IL-15 production was measured using the human IL-15 Quantikine ELISA kit (R&D) by the manufacturer’s instructions. For IL-15 detection in mice plasma, samples were diluted with a PBS solution to a 1:3 ratio before the analysis.

### Animal study

All animal studies were approved by the Ethics Committee from the Second Affiliated Hospital of Zhejiang University School of Medicine (Number: AIRB-2021-853) in compliance with Chinese National Laboratory Animal Guideline for Ethical Review of Animal Welfare. Six- to 8-week-old, female NSG (NOD-*Prkdc*^*scid*^ *IL2rg*^*tm1*^/Bcgen) mice (Biocytogen) were inoculated intravenously with JeKo-1-luc2GFP (1 × 10^4^) on day −3. In total, 2 × 10^5^ NK, 18.2-BBz CAR-NK, or CD19-BBz CAR-NK cells were injected through the tail vein on day 0. Mice were subjected to bioluminescent imaging (IVIS Lumina LT In Vivo Imaging System, PerkinElmer) on day 3 and weekly afterwards. Signal quantitation in photons per second was performed by determining the photon flux rate within standardized regions of interest using Living Image software (PerkinElmer, version 4.7.4). According to the guidelines for the welfare and use of animals of the Ethics Committee, the maximal tumor burden allowed was 1 × 10^10^ total flux (photons s^−1^). In some cases, this limit was exceeded on the last day of measurement, and the mice were immediately euthanized. Survival of JeKo-1-bearing mice in each group of mice was estimated using the Kaplan–Meier method and analyzed with a log-rank test. At the end of the experiments, the remaining survival mice in CAR-NK treatment group were killed, and distribution as well as persistence of CAR-NK cells in bone marrow, blood, and spleen were analyzed using flow cytometry. Gating strategy to identify CAR^+^ NK cells are shown in Supplementary Fig. 1b. Plasma of peripheral blood from CAR-NK cell-treated mice were collected for evaluation of IL-15 secretion.

### Manufacture of CD19-BBz CAR-NK cells

Human cord blood units for this study were provided by Zhejiang Blood Center Cord Blood Bank, which was approved by the Ethic Committee of Zhejiang Blood Center. All donors provided written informed consent. CAR-NK cell manufacture was performed in the GMP Laboratory of the Second Affiliated Hospital, College of Medicine, Zhejiang University. Cord blood mononuclear cells (CBMCs) were isolated by Ficoll density gradient centrifugation. T cells were depleted using CD3 microbeads (Militenyi Biotec) according to the manufacturer’s instructions. The CD3^−^ CBMCs (<0.1%) were stimulated with K562 feeder cells at a 2:1 ratio. Cells were maintained in OpTmizer CTS T-Cell Expansion SFM basic Medium (Gibco) with 2 mmol l^−1^ GlutaMAX (Thermo Fisher), 10% human AB serum (Sigma), and 1,000 IU ml^−1^ human IL-2 (Quangang). On day 5 of culture, the phenotypes of culture cells and feeder cells were detected by flow cytometry using a panel of antibodies that are listed in Supplementary Table 3, and the detecting strategies are described in Supplementary Fig. 1b. NK cells were subsequently transduced with a BaEV-LV encoding CD19-BBz CAR at a multiplicity of infection of 5. Two days later, a second round of feeder cells stimulation was performed for CAR-NK cell expansion. On day 14, CAR-NK cells were collected for clinical infusion. Release criteria for the CAR-NK cell products are listed in Supplementary Table 4.

### Detection of CAR-NK cells by flow cytometry

To determine the proliferation and persistence of CAR-NK cells, CAR^+^ NK cells in the peripheral blood were assessed by a multiparameter flow cytometry. Blood samples were lysed to remove red blood cells by a lysing solution (BD). Cells were then suspended in PBS buffer containing 2% FBS and 0.1% Na_3_N. After staining with Pacific Blue-anti-CD45, APC/Cy7-anti-human CD3, Alexa Fluor 647-conjugated anti-mouse FMC63 scFv monoclonal antibody, PE-anti-CD56, and Percy-cy5.5 anti-CD16 for 15 min at room temperature, cells were washed by centrifugation at 400 × *g* and loaded on a NovoCyte flow cytometer (ACEA Biosciences). NovoExpress software (version 1.5.0) was used for data analysis. All fluorophore antibodies are listed in Supplementary Table 3, and the gating strategy is shown in Supplementary Fig. 1c.

### Monitoring of CAR-NK cells in blood by digital droplet PCR

ddPCR was used to monitor the CAR-NK cell persistence in blood as previously described^56^. Due to the high concentration of internal reference genes, the two genes were separated into two independent reaction systems for better target gene (FMC63) detection. The reaction mix was composed of 12.5 μl of 2× ddPCR Universal Mastermix for DNA (with UDG, Maccura), 1.25 μl FAM-labeled FMC63 primer-probe-assay, 0.1 μl XbaI, 0.1 μl Mlu I, 0.1 μl HindIII-HF restriction enzyme (NEB), 10–660 ng genomic DNA (volume 2–5 μl), and nuclease-free water to a final volume of 25 μl. The reaction was incubated for 15 min at 37 °C for restriction digestion, and then droplets were generated. The ddPCR was run using a D 600 Digital PCR System (Maccura) with the cycling conditions as follows: initial denaturation at 95 °C for 5 min, amplification with 45 cycles at 95 °C for 20 s, and 60 °C for 30 s. The ramp rate was set to 2.0 °C s^−1^. Finally, droplets were analyzed, and data were processed with Digital PCR System, including Poisson’s distribution analysis that enables absolute quantification of the target. The copies of target gene per droplet was calculated based on the number of negative droplets in the sample. Data of absolute copies per µl of the target gene in the sample volume was calculated with the following formula: *Z* (copies per μl) = *X* × 25 (total volume)/*Y* (sample volume). *X* is the quantitative value provided by the D 600 Digital PCR system; *Y* is the input sample volume; *Z* is the sample concentration. The concentration of each internal reference gene was calculated by multiplying the output result with the dilution ratio 125. Then, the copies per µl of the internal reference gene was converted to µg µl^−1^ through the following formula: *A* (µg µl^−1^) = *B* (copies per µl) × 3.3/1,000,000. *A* represents the concentration of internal reference (µg µl^−1^) and *B* represents the number of copies per μl of internal reference (*Z*) × 125. Finally, the quantitative values of the target gene (copies per μg) were calibrated with the corresponding quantitative values of the internal reference genes as *Z* (FMC63, copies per μl)/*A* (internal reference gene, μg µl^−1^).

### Real-time quantitative reverse transcription PCR

PBMCs were isolated from heparinized blood by Ficoll-Hypaque at 400 × *g* for 30 min. The PBMCs (5 × 10^6^ ml^−1^) of patients were obtained. Following extraction of total RNA, quantitative reverse transcription PCR was performed to detect the expression of *CBLB* messenger RNA. Fold changes relative to *GAPDH* were calculated with 2^−ΔΔCt^.

All primers and probes used in this study are detailed in Supplementary Table 5.

### Soluble factor Luminex multiplex assay

Patient serum samples were collected before and after CAR-NK infusion. The soluble cytokines were quantified on a Luminex 200 instrument (Millipore). A human ProcartaPlex multiplex immunoassay kit (ThermoFisher) was used to measure 34 cytokines that are listed in Supplementary Table 6. Luminex assays were performed by following the manufacturer’s protocol. Raw data were processed with the Milliplex Analyte program (version 5.1.0.0) by using five-parameter logistic regression analysis.

All commercial reagents used in this study are listed in Supplementary Table 7.

### Single-cell transcriptomic analysis

The CAR-NK cell products for patients and mononuclear cells from patient’s blood on day 6 after infusion were prepared into single-cell suspensions and entered in the 10X scRNA-seq workflow. To prepare the complementary DNA libraries for the 10X Genomics Chromium controller, we used the single-cell 3′ v2 kit. We processed gene expression FASTQ files generated on an Illumina sequencer using the Cell Ranger pipeline (version 7.0.1, 10x Genomics) for read alignment and generation of feature barcode matrices. The output files were then uploaded into R (version 4.3.3) for further processing using Seurat (version 4.4.0). We developed a pipeline to process data. In brief, cells with >300 detected genes and a mitochondrial read percentage <40 were retained. Doublets or multiplet cells were identified with the scDblFinder (version 1.16.0) R package and excluded. Finally, we obtained 89,658 cells from the prepared seven CAR-NK cell products and 51,068 cells from PBMCs of five donors. After normalization of the Seurat object, we separately selected the top 3,000 and 7,000 highly variably expressed genes using the FindVariableFeatures function. Then principal component analysis was performed using the highly variably expressed genes, and reciprocal principal component analysis algorithm was used to remove batch effects. Then we applied the RunUMAP function to reduce dimensionality and visualize the two-dimensional single-cell clusters using default values (number of neighbors is 30 and minimum distance is 0.3). Next, we used the FindAllMarkers function to detect DEGs between clusters. Dot plots were made based on the most highly expressed genes (according to fold change) which had an adjusted *P* value less than 0.05.

For each sample, the cell-type proportion was calculated by the number of cells in a certain cell type divided by the total number of cells.

To evaluate whether a hallmark or signature gene set was enriched within the specific subtypes, we calculated a gene set activity score for each gene set via AUCell (v 2.6.2, R package). Each cell can be colored based on their area under the curve scores with uniform manifold approximation and projection (UMAP) embedding for visualization.

Gene set enrichment analysis coupled with the gene set data of KEGG and Gene Ontology was applied to explore changes of signaling pathways among different clusters. We used adjusted *P* < 0.01 to select the significantly altered gene sets. The normalized enrichment scores were set to 0 when differences were not significant. GSVA is a non-parametric, unsupervised method for estimating variations in gene set enrichment among the samples or subsets of an expression dataset. Pathways and gene sets were downloaded from the MsigDB (v7.5.1) in combination with curated specific published gene sets^57^.

We divided the significative gene sets based on GSVA analysis into five categories, referred to as set1, set2, set3, set4, and set5. To clarify the correlation between these gene sets, we applied the R package “ggscatter” (version 0.6.0) and “Corrplot” (version 0.92) to calculate correlation coefficient and significance according to the five gene set enrichment scores.

High-dimensional weighted gene co-expression network analysis (v 0.3.01) package was used to clarify the key molecular characteristics of the CAR-NK cell products with different states. Using a soft threshold of 9, a scale-free network was constructed for optimal connectivity, resulting in the identification of 6 gene modules, followed by KEGG enrichment by enrichR (v3.2) package for each module.

To infer cell–cell interaction changes in the immune cells, we used the R package CellChat (v 2.1.2). Briefly, we used the CellChatDB.human database to compare outgoing and incoming signal patterns between different immune cells and inferred the cell–cell communication at the signal pathway level, to further identify the cell–cell communication differences.

### Clinical study design and patients

We conducted a single-center, single-arm, open-label, phase 1 trial of CD19-BBz CAR-NK cells in adult patients with R/R LBCL. This study was approved by the Ethic Committee of the Second Affiliated Hospital of Zhejiang University School of Medicine and registered on ClinicalTrial.gov (NCT05472558, July 25, 2022). This study was also authored by the Health Commission of Zhejiang province. All participants provided written informed consent in accordance with the Declaration of Helsinki and consented to publish their clinical information. We offered commercial insurance by Asia-Pacific Property & Casualty Insurance to every participant in our study. The insurance covered potential casualties incurred by the clinical study. The first participant was enrolled on July 26, 2022, and the last on December 9, 2022. No sex-specific analyses were performed. The full clinical study protocol is included in Supplementary Information. Patients received lymphodepletion chemotherapy consisting of cyclophosphamide (750 mg m^−^^2^ day^−1^, day −5 to day −3) and fludarabine (30 mg m^−2^ day^−1^, day −6 to day −3). Any bridging therapy and additional antitumor treatment after infusion was not permitted. On day 0, day 7, and day 14, patients received escalating doses of CD19-BBz CAR-NK cells (2 × 10^6^, 3 × 10^6^, and 4 × 10^6^ CAR^+^ NK cells per kg of patient body weight). Two patients were assigned to cohort 1 because another patient who received 2 × 10^6^ kg^−1^ CD19-BBz CAR-NK cells in our previous exploratory study did not show any ≥grade 1 CRS/neurotoxicity or CAR-NK cell-related adverse events.

Eligible patients were adults aged 18–75 years with CD19^+^ R/R LBCL, including at least one measurable tumor, with an Eastern Cooperative Oncology Group performance status of ≤2, an absolute neutrophil count of ≥1,000 µl^−1^, and a platelet count of ≥45,000 µl^−1^. Additional eligibility included adequate renal, hepatic, and cardiac function. Patients who received previous autologous CD19 CAR T-cell therapy within 12 weeks were excluded. Detailed inclusion and exclusion criteria are listed in Supplementary Table 8.

The primary end points were safety, MTD, and ORR of treatment with CD19-BBz CAR-NK cells within 30 days of CAR-NK cell infusion. CRS and ICANS were graded with the American Society of Transplantation and Cellular Therapy Consensus grading system^58^, and other adverse events were evaluated according to the Common Terminology Criteria for Adverse Events (CTCAE v. 4.03). Secondary end points included duration of responses (DORs), overall survival, and PFS. Assessment of levels of CAR-NK in blood, cytokines in serum, and single RNA sequencing were exploratory objectives. Response assessment was performed using PET–CT at day 30 according to the Lugano 2014 classification^59^, and then ongoing response was monitored by ultrasound and CT scan every 3 months until disease progression. Metabolic tumor volume at baseline was determined as previously described^59^.

### Statistical analysis and reproducibility

This phase 1 study was primarily designed to evaluate the safety and the response of CD19-BBz CAR-NK cells, and thus, the study variables were analyzed using descriptive statistics. A standard 3 + 3 dose-escalation design was implemented to determine the MTD. Although the initial cohort at 2.0 × 10^6^ cells per kg was planned to enroll three patients, the first patient in this cohort was enrolled before the official registration. Despite no adverse events being observed in this patient, per protocol requirements, data from this case were excluded from DLT evaluation to ensure compliance with institutional review board guidelines. Consequently, two subsequently enrolled patients completed full treatment and evaluation at this dose level, with no DLTs observed. This allowed dose escalation to proceed as per the predefined protocol criteria. No statistical methods were used to pre-determine sample sizes. The probabilities of overall survival and PFS were estimated using Kaplan–Meier method. PFS and overall survival were defined as the time from CAR-NK cells infusion to first relapse or death, with censoring at the last follow-up. DOR was defined only for participants with a response at day 30 and is the time from the first responses to disease progression or death, with censoring at the last follow-up. No data were excluded from analysis, and the experiments were not randomized. The investigators were not blinded to allocation during experiments and outcome assessment. The statistical details of preclinical study are reported in the figure legends. Animal in vivo experiments were randomized to each group. The statistical description in transcriptome analysis varies depending on the data type, including mean ± s.e.m., median, quartile, or extremum. The inspection method is described in the legend. Briefly, intergroup analysis of independent samples used independent *t*-test, while comparison of the same sample before and after used paired *t*-test. Data distribution was assumed to be normal, but this was not formally tested. Statistical analyses were done using GraphPad Prism version 9 software and R version 4.0.3 software. *P* < 0.05 was considered significant. Each experiment was repeated at least two times independently with similar results.

### Reporting summary

Further information on research design is available in the Nature Portfolio Reporting Summary linked to this article.

## Supplementary information

Supplementary Information Supplementary Fig. 1, Tables 1–8 and clinical trial protocol.

Reporting Summary

## Source data

Source Data Fig. 1 Statistical source data for Fig. 1.

Source Data Fig. 3 Statistical source data for Fig. 3.

Source Data Fig. 4 Statistical source data for Fig. 4.

Source Data Fig. 5 Statistical source data for Fig. 5.

Source Data Fig. 6 Statistical source data for Fig. 6.

Source Data Extended Data Fig. 1 Statistical source data for Extended Data Fig. 1.

Source Data Extended Data Fig. 2 Statistical source data for Extended Data Fig. 2.

Source Data Extended Data Fig. 3 Statistical source data for Extended Data Fig. 3.

Source Data Extended Data Fig. 4 Statistical source data for Extended Data Fig. 4.

Source Data Extended Data Fig. 5 Statistical source data for Extended Data Fig. 5.

Source Data Extended Data Fig. 6 Statistical source data for Extended Data Fig. 6.

Source Data Extended Data Fig. 7 Statistical source data for Extended Data Fig. 7.

Source Data Extended Data Fig. 8 Statistical source data for Extended Data Fig. 8.

Source Data Extended Data Fig. 9 Statistical source data for Extended Data Fig. 9.

Source Data Extended Data Fig. 10 Statistical source data for Extended Data Fig. 10.

## Data Availability

All raw sequencing data generated in this study have been deposited in the National Genomics Data Center under the accession code HRA006106 (https://ngdc.cncb.ac.cn/gsa-human/browse/HRA006106) and HRA008206 (https://ngdc.cncb.ac.cn/gsa-human/browse/HRA008206). The raw sequencing data are available under controlled access in accordance with Genome Sequence Archive (GSA) controlled management regulations. Access to the data can be requested by completing the application form via GSA-Human System and is granted by the corresponding Data Access Committee. Additional guidance can be found at the GSA-Human System website (https://ngdc.cncb.ac.cn/gsa-human/document/GSA-Human_Request_Guide_for_Users_us.pdf). The clinical trial study protocol is available in the Supplementary Information file. Source data are provided with this paper. Individual clinical data cannot be made publicly available for patient privacy, but additional de-identified individual participant clinical data are available from the corresponding authors upon request. The remaining data are available within the Article and Supplementary Information.
