# Author Correction: Safety and feasibility of 4-1BB co-stimulated CD19-specific CAR-NK cell therapy in refractory/relapsed large B cell lymphoma: a phase 1 trial

**DOI:** 10.1038/s43018-025-01026-w

**Published:** 2025-07-03

**Authors:** Wen Lei, Hui Liu, Wenhai Deng, Wei Chen, Yun Liang, Wenxia Gao, Xianggui Yuan, Shanshan Guo, Ping Li, Jinyong Wang, Xiangmin Tong, Yi Eve Sun, Aibin Liang, Wenbin Qian

**Affiliations:** 1 Department of Hematology, the Second Affiliated Hospital, College of Medicine, Zhejiang University, Hangzhou, China; 2 Key Laboratory of Cancer Prevention and Intervention, China National Ministry of Education; Biotherapy Research Center, the Second Affiliated Hospital, College of Medicine, Zhejiang University, Hangzhou, China; 3 Key Laboratory of Laboratory Medicine, Ministry of Education, School of Laboratory Medicine and Life Sciences, Wenzhou Medical University, Wenzhou, China; 4 Stem Cell Translational Research Center, Tongji Hospital, Tongji University, School of Medicine, Shanghai, China; 5 Department of Hematology, Tongji Hospital of Tongji University, Shanghai, China; 6 State Key Laboratory of Stem Cell and Reproductive Biology, Institute for Stem Cell and Regeneration, Institute of Zoology, Chinese Academy of Sciences, Beijing, China; 7 Department of Hematology, Affiliated Hangzhou First People’s Hospital, School of Medicine, Westlake University, Hangzhou, China

**Keywords:** B-cell lymphoma, Cancer immunotherapy, Cancer

Correction to: *Nature Cancer* 10.1038/s43018-025-00940-3, published online 18 April 2025.

In the version of the article initially published, due to an error in figure assembly, the sixth mouse in the “19 CAR-NK” (Day 2) images in Fig. 1d was a duplicate of the second mouse in the same group. Additionally, the “18.2 CAR-NK” (Day 37) mouse image was a duplicate of the sixth “18.2 CAR-NK” (Day 30) mouse directly above it. The amended figure with the correct images is now available in the HTML and PDF versions of the article.

