# Supplementary material for: Safety and feasibility of 4-1BB co-stimulated CD19-specific CAR-NK cell therapy in refractory/relapsed large B cell lymphoma: a phase 1 trial
Source: Nat Cancer. 2025 Apr 18;6(5):786–800. doi: 10.1038/s43018-025-00940-3 (PMC12122374; doi:10.1038/s43018-025-00940-3)
Supplement: Supplementary file 1 — Supplementary Fig. 1, Tables 1–8 and clinical trial protocol. [file 43018_2025_940_MOESM1_ESM.pdf]

nature cancer

Supplementary information

https://doi.org/10.1038/s43018-025-00940-3

Safety and feasibility of 4-1BB co-stimulated CD19-specific CAR-NK cell therapy in refractory/relapsed large B cell lymphoma: a phase 1 trial

In the format provided by the authors and unedited

Supplementary Figure 1 Gating strategies for Flow cytometry

a

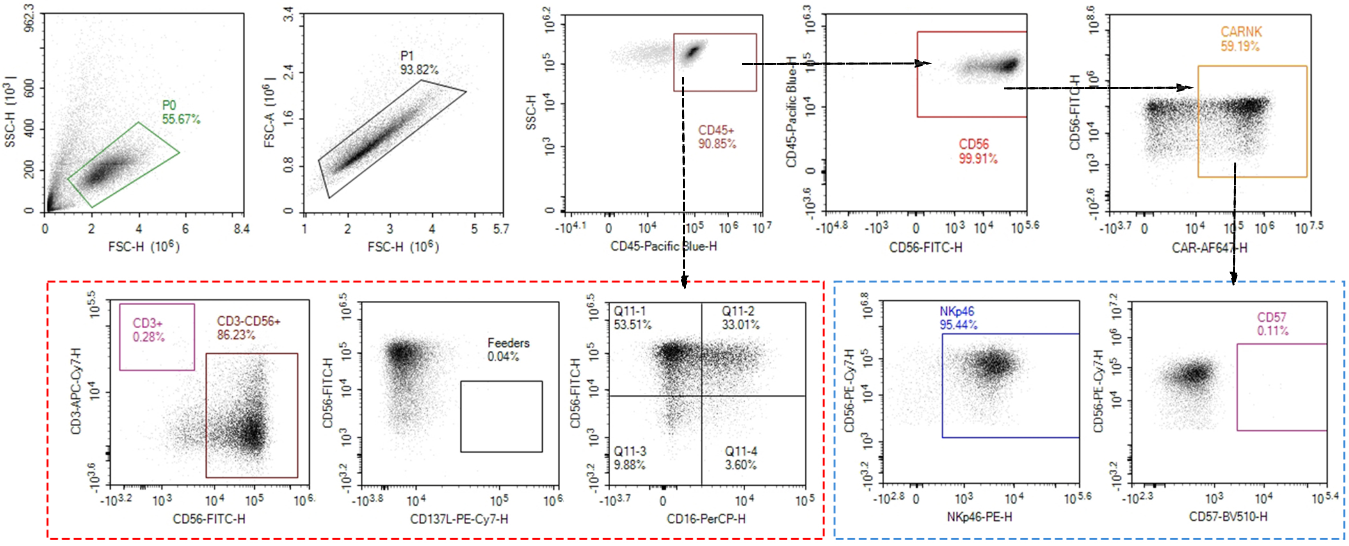

Phenotypes of CAR-NK cell products in Fig. 1b and Supplementary Table 2.

b

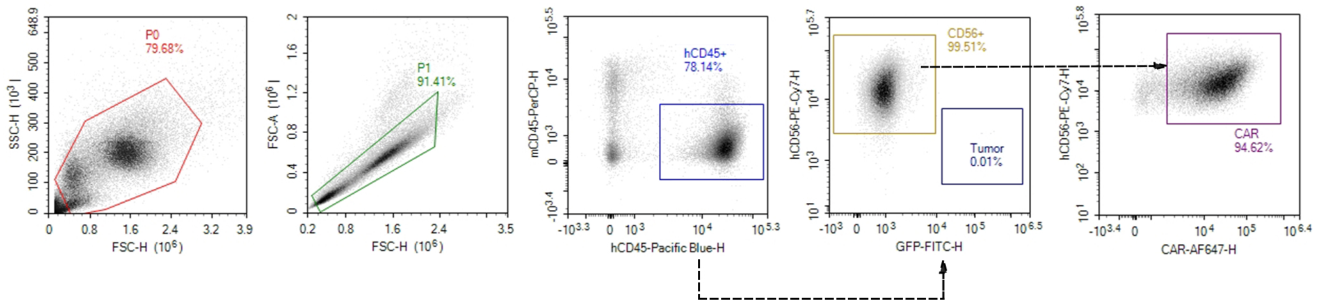

Absolute numbers of CAR⁺NK cells in blood, spleen and bone marrow in Fig. 1g.

c

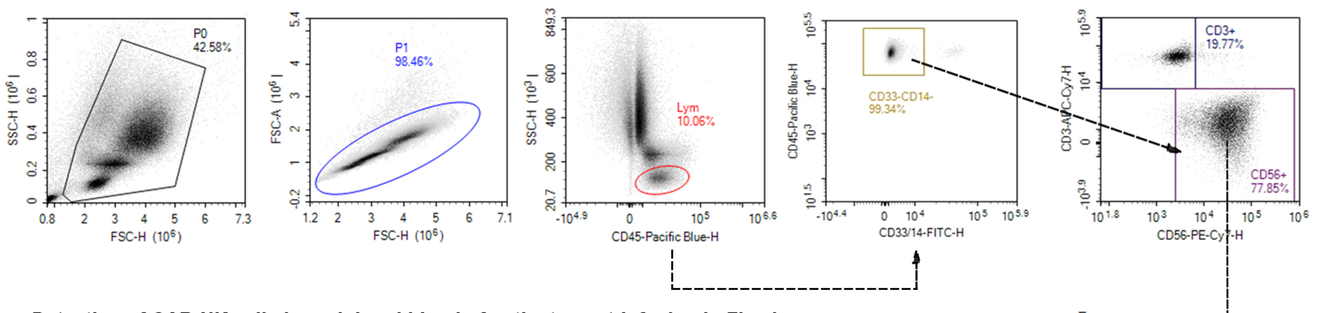

Detection of CAR-NK cells in peripheral blood of patients post-infusion in Fig. 4a.

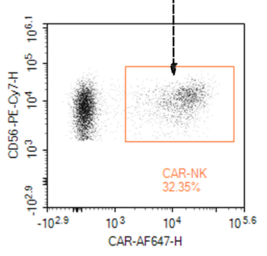

d

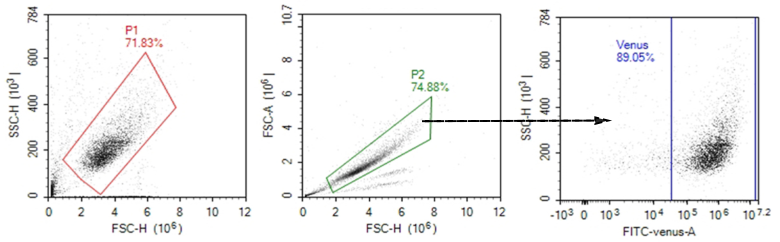

Transduction efficiency of BaEV-LV in NK92MI cells in Extended Data Fig. 1

Supplementary Table 1. Patient's Characteristics

Patient. NO.	Age	Sex	Diagnosis	Prior Therapy (lines)	Prior CAR-T Treatment	HLA Allelic Match#	KIR ligand Mismatch&	NO. of cord blood unit (CBU)	MTV (cm^3)
1	66	Male	Diffuse large B-cell lymphoma (Non-GCB)	3	None	1/12	YES	202205674	243.45
2	72	Male	Mantle cell lymphoma	4	CD19 CAR-T	N/A	N/A	202206394	22.31
3	67	Female	Diffuse large B-cell lymphoma (GCB)	7	CD19 CAR-T	2/12	YES	202206394	65.81
4	69	Female	Diffuse large B-cell lymphoma (Non-GCB)	3	None	5/12	YES	202201487	30.37
5	73	Female	Diffuse large B-cell lymphoma (GCB)	7	None	1/12	YES	202201487	156.88
6	48	Male	Diffuse large B-cell lymphoma (Non-GCB)	5	None	5/12	YES	202201250	6.52
7	67	Male	Diffuse large B-cell lymphoma (Non-GCB)	3	None	6/12	YES	202223019	126.78
8	60	Male	Diffuse latge B-cell lymphoma (Non-GCB)	5	CD19 CAR-T	4/12	YES	202231436	405.27

#Listed is the number of HLA matches between the donated cord-blood unit and the patient at HLA loci A, B, C, DRβ1, DQβ1 and DPβ1.& The KIR ligand Mismatch has three mismatch model including Ligand mismatch, Receptor mismatch and Receptor-ligand mismatch. NA, not available.

Supplementary Table 2. Characteristics of the CAR-NK cell products

Patient No.	Infused CAR^+ cells/kg	$CD3^+$T cell in products （%）	Feeder cell residual （%）	$CD16^-CD56^+$ （%）	$CD16^+CD56^+$ （%）	$CD16^+CD56^-$ （%）	$CD16^-CD56^-$ （%）	CAR^+ （%）	$NKp46^+$ （%）	$CD57^+$ （%）	Response for CAR-NK treatment
1	2.0×10^6	0.84%	0.11%	33.42%	62.63%	0.72%	3.24%	43.42%	77.54%	0.15%	CR
	2.0×10^6	2.14%	0.17%	27.52%	57.19%	4.13%	11.15%	30.99%	78.95%	0.00%	
	2.0×10^6	3.26%	0.26%	31.44%	57.15%	2.50%	8.91%	39.82%	80.79%	0.63%	
2	2.0×10^6	0.69%	0.00%	10.48%	86.22%	1.24%	2.06%	39.70%	88.59%	0.49%	PD
	2.0×10^6	0.50%	0.00%	55.24%	34.19%	0.71%	9.85%	55.11%	94.72%	0.28%	
	2.0×10^6	0.15%	0.00%	40.35%	58.81%	0.04%	0.79%	42.30%	95.91%	0.03%	
3	3.0×10^6	0.13%	0.49%	46.64%	47.86%	0.25%	5.25%	33.13%	97.42%	2.10%	PR
	3.0×10^6	0.44%	0.00%	19.73%	75.73%	1.78%	2.76%	20.39%	97.34%	0.14%	
	3.0×10^6	0.77%	0.00%	25.67%	58.27%	4.80%	11.27%	46.66%	95.40%	0.25%	
4	3.0×10^6	0.01%	0.45%	6.32%	78.71%	11.68%	3.28%	24.31%	84.25%	7.20%	SD
	3.0×10^6	0.45%	0.01%	23.87%	62.50%	4.48%	9.15%	27.62%	90.94%	0.30%	
	3.0×10^6	0.39%	0.02%	37.40%	54.83%	3.20%	4.57%	16.46%	97.73%	0.06%	
5	3.0×10^6	0.49%	0.03%	36.29%	55.40%	2.13%	6.18%	31.56%	97.67%	0.03%	CR
	3.0×10^6	0.64%	0.06%	42.49%	48.87%	1.94%	6.70%	28.09%	96.50%	0.03%	
6	4.0×10^6	0.30%	0.10%	33.55%	58.86%	1.17%	6.41%	50.32%	96.97%	0.08%	CR
	4.0×10^6	0.56%	0.40%	29.42%	50.27%	1.03%	19.28%	42.16%	81.01%	0.42%	
	4.0×10^6	0.32%	0.00%	46.79%	48.55%	0.04%	4.62%	48.92%	97.71%	0.26%	
7	4.0×10^6	0.29%	0.01%	22.12%	75.81%	0.54%	1.53%	57.93%	99.03%	0.56%	CR
	4.0×10^6	0.48%	0.00%	24.33%	72.68%	0.71%	2.29%	42.02%	97.28%	0.12%	
	4.0×10^6	0.54%	0.00%	6.87%	85.28%	2.92%	4.93%	30.84%	96.96%	0.18%	
8	4.0×10^6	0.04%	0.09%	32.96%	65.34%	0.00%	1.70%	14.03%	95.93%	0.04%	PD
	4.0×10^6	0.37%	0.00%	28.42%	68.83%	0.67%	2.09%	48.73%	96.96%	0.14%	
	4.0×10^6	0.23%	0.02%	33.95%	58.40%	1.76%	5.90%	59.47%	97.27%	0.07%	

Phenotypes of CAR-NK cell products detected by Flow cytometry CD56$^+$CAR$^+$NK cells were gated in the CD45$^+$cells, and CD56/CD16 were identified as the phenotypes of CAR-NK cells; CD3$^+$T cell and CD137L$^+$ feeder cells were also detected in the CD45$^+$cell to exclude the contamination of T-cell and feeder cells in the CAR-NK products. The expressions of NKp46 and CD57 were detected in CAR$^+$NK cells. Gating strategies were showed in Supplementary Figure 1a.

Supplementary Table 3. Flow cytometry panels for CAR-NK detection

Antibody	Fluorescence	catalog numbers	Clone	Origin	dilutions	validation
**Panel A CARNK detection in blood, spleen, and bone marrow**						
anti-mouse CD45	PerCP	103130	30-F11	Biolegend	1:250	N/A
Anti-human CD45	Pacific Blue	304029	HI30	Biolegend	1:200	N/A
Anti-human CD56	PE	362524	5.1H11	Biolegend	1:500	N/A
Anti-mouse FMC63 scFv	Alexa Fluor 647	200102	R19M	BioSwan Lab.	1:500	N/A
**Panel B CARNK product immunophenotype**						
Anti-human CD45	Pacific Blue	304029	HI30	Biolegend	1:200	N/A
Anti-human CD56	FITC	362546	5.1H11	Biolegend	1:250	N/A
Anti-human CD16	PerCP	302030	3G8	Biolegend	1:500	N/A
Anti-human CD3	APC-Cy7	300426	UCHT1	Biolegend	1:250	N/A
Anti-human CD137L	PE-Cy7	311512	5F4	Biolegend	1:500	N/A
Anti-mouse FMC63 scFv	Alexa Fluor 647	200102	R19M	BioSwan Lab.	1:500	N/A
Anti-human NKp46	PE	331908	9E2	Biolegend	1:500	N/A
Anti-human CD57	Brilliant Violet	393314	QA17A04	Biolegend	1:200	N/A
Anti-human CD45	Pacific Blue	304029	HI30	Biolegend	1:200	N/A
**Panel C CARNK detection in human peripheral blood post-infusion**						
Anti-human CD45	Pacific Blue	304029	HI30	Biolegend	1:200	N/A
Anti-human CD14	FITC	301804	M5E2	Biolegend	1:250	N/A
Anti-human CD33	FITC	381704	S21002C	Biolegend	1:250	N/A
Anti-human CD3	APC-Cy7	300426	UCHT1	Biolegend	1:250	N/A
Anti-human CD56	PE-Cy7	362510	5.1H11	Biolegend	1:500	N/A
Anti-mouse FMC63 scFv	Alexa Fluor 647	200102	R19M	BioSwan Lab.	1:500	N/A
Anti-human CD45	Pacific Blue	304029	HI30	Biolegend	1:200	N/A

Supplementary Table 4. Release criteria for the CAR-NK cell products

**Test item**	**Standard criterion**
Outlooking	No flocculent precipitate, no foreign body
Cell viability	≥ 90%
$CD56^+$ NK cells	≥ 80%;
CAR transduction	≥ 10%.
$CD3^+$ T cell	≤ 1.0%;
Feeder cells	≤ 1.0%
RCL Test	Negative
Bacterial	Sterile at least culture for 5 days
Fungal	Sterile at least culture for 5 days
Mycoplasma	Negative
Endotoxin	≤ 0.5%

Supplementary Table 5. Sequence of all the oligos in the manuscripts

**Name**	**Sequence**
FMC63-F:	5'-AGCTGCTGATCTATCACACCTC-3'
FMC63-R:	5'-TGCTGGCAAAAATAGGTGGC-3'
FMC63 Probe	FAM 5'-CGCTTTTCCGGCTCTGGCAGCGGCACA-3'BHQ1
h-β-actin-F:	5'-TCCGTGTGGATCGGCGGCTCCA-3'
h-β-actin-R:	5'-CTGCTTGCTGATCCACATCTG-3'
h-β-actin-Probe:	5'- HEX CCTGGCCTCGCTGTCCACCTTCCA-3 BHQ1.
h-CBLB-F:	5'-AATCCCCGAAAAGGTCGAATTT-3'
h-CBLB-R:	5'-CACAGTCTTACCACTTTGTCCAT-3'
h-GAPDH-F:	5'-GGAGCGAGATCCCTCCAAAAT-3'
h-GAPDH-R:	5'-GGCTGTTGTCATACTTCTCATGG-3'

Supplementary Table 6. List of 34 cytokines in the serum of patients detected by Luminex multiplex assay

Number	Protein name
1	Macrophage inflammatory protein-1-alpha (MIP-1alpha)
2	Stromal cell-derived factor-1α (SDF-1alpha)
3	IL-27
4	IL-1-beta
5	IL-2
6	IL-4
7	IL-5
8	IFN-gamma-inducible protein-10 (IP-10, CXCL10)
9	IL-6
10	IL-7
11	IL-8
12	IL-10
13	Eotaxin
14	IL-12p70
15	IL-13
16	IL-17A
17	IL-31
18	IL-1RA
19	RANTES
20	Interferon (IFN)-gamma
21	Granulocyte macrophage-colony stimulating factor (GM-CSF)
22	Tumor necrosis factor (TNF)-alpha
23	Macrophage inflammatory protein-1-beta (MIP-1beta)
24	Interferon (IFN)-alpha
25	Monocyte chemoattractant protein (MCP-1, CCL2)
26	IL-9
27	Tumor necrosis factor (TNF)-beta
28	GRO-alpha
29	IL-1alpha
30	IL-23
31	IL-15
32	IL-18
33	IL-21
34	IL-22

Supplementary Table 7. Information of all commercial reagents in this manuscript

Names	Company names	Catalog numbers
D-luciferin potassium salt	PerkinElmer	122799
Poly-l-lysine	Sigma-Aldrich	25988-63-0
Human IL-15 Quantikine ELISA Kit	R&D	D1500
CD3 MicroBeads	Miltenyi Biotec	130-050-101
GlutaMAX	Thermo Fisher	35050061
Human AB serum	Sigma-Aldrich	H4522
Human IL2	Quangang Pharmaceutical	125SER
FACS Lysing solution	BD	555899
Universal master mix	Maccura	GN7200068
ProcartaPlex multiplex immunoassay kit	Thermo Fisher	EPX340-12167-901
XbaI	NEB	R0145V
Mlu I	NEB	R3198V
HindIII-HF	NEB	R3104V
ProLong Gold Antifade Mountant	Thermo Fisher	P10144

Supplementary Table 8. Inclusion and Exclusion Criteria

**Inclusion Criteria**	1. All subjects in this study can understand and are willing to sign the written informed consent;
	2. Aged 18-75 years, male or female;
	3. Histologically confirmed diffuse large B-cell lymphoma (DLBCL), transformed follicular lymphoma (TFL), primary mediastinal B-cell lymphoma (PMBCL), mantle cell lymphoma (MCL), and other transformed indolent lymphomas:<br>(1) Refractory or relapsed DLBCL: patients did not achieve complete remission after 2-line therapy; progression of the disease during any lines of treatment or a period of disease stabilization equal to or less than 6 months; or disease progression/relapse within 12 months after autologous hematopoietic stem cell transplantation;<br>(2) Refractory or relapsed MCL patients who must be resistant to or inability to tolerate BTK inhibitors;<br>(3) Refractory or relapsed indolent B-cell NHL patients with third-line therapy failure or recurrence;<br>(4) Prior treatment must include CD20 monoclonal antibody therapy (unless subjects are CD20-negative) and anthracyclines;
	4. There was at least one measurable lesion with the longest diameter ≥ 1.5 cm;
	5. Expected survival ≥ 12 weeks;
	6. The expression of CD19 in tumor tissue biopsy was positive;
	7. ECOG score 0-2;
	8. Adequate organ function:<br>(1) Alanine aminotransferase, Aspartate aminotransferase ≤ 2.5 × UNL (upper limit of normal);<br>(2) Creatinine clearance (Cockcroft-Gault method) ≥ 60 mL/min;<br>(3) Serum total bilirubin and alkaline phosphatase ≤ 1.5 × UNL;<br>(4) Glomerular filtration rate >50 mL/min<br>(5) Cardiac ejection fraction (EF) ≥ 50%;<br>(6) Basic oxygen saturation > 92% in an indoor natural air environment
	9. One previous stem cell transplant is permitted
	10. Approved anti-B-cell lymphoma therapies, such as systemic chemotherapy, whole-body radiotherapy, and immunotherapy, must have been stopped at least 3 weeks before enrollment;
	11. Accept patients who have previously received CAR T cell therapy and failed to respond on 3 months evaluation or relapse;

	12. Female subjects with childbearing potential must have a negative pregnancy test and agree to use effective contraception during the study;
	13. Subjects should have negative tests for coronavirus covid-19.
**Exclusion Criteria**	1. Allergic to any of the components of cell products;
	2. History of other tumors;
	3. Acute GvHD or extensive chronic GvHD with grade II-IV (Glucksberg standard) in the past or are receiving anti-GVHD treatment;
	4. Had received gene therapy within the past 3 months;
	5. Active infections requiring treatment (except for simple urinary tract infections, bacterial pharyngitis); however, prophylactic antibiotics, antiviral and antifungal infection treatment are permitted;
	6. Patents infected with hepatitis B (HBsAg positive, but HBV-DNA < 10^3 is not excluded) or hepatitis C virus (including virus carriers), syphilis and other acquired and congenital immunodeficiency diseases, including but not limited to HIV-infected persons;
	7. Subjects with Grade III or IV cardiac dysfunction according to the New York Heart Association's cardiac function grading criteria;
	8. Patients who received antitumor therapy earlier but did not recover from the toxicity (CTCAE 5.0 toxicity did not recover to ≤ grade 1, except fatigue, anorexia, alopecia);
	9. Subjects with a history of epilepsy or other central nervous system disorders;
	10. Head-enhanced CT or MRI showing evidence of central nervous system lymphoma;
	11. Lactating women who are unwilling to stop breastfeeding;
	12. Any other factors that the investigator believes may increase the risk to the subject or interfere with the test results.

Clinical Trial Protocol

Clinical Study of Cord Blood-Derived CAR-NK Cells Targeting CD19 in the Treatment of Refractory/Relapsed B-Cell NHL

Protocol Number: CB CAR-NK019-001

Institution: Department of Hematology, The Second Affiliated Hospital, College of Medicine, Zhejiang University

Principal Investigator：Wenbin Qian M.D. & Ph.D.

Department of Hematology, the Second Affiliated Hospital,

College of Medicine, Zhejiang University, Hangzhou,

Zhejiang, 310009, China

Version: 1.0

Date: June 15, 2022

Study implementation: In accordance with the principles of the Declaration of Helsinki and ethical principles consistent with the International Coordination Conference Guidelines for the Quality Management of Pharmaceutical Clinical Trials and applicable regulatory requirements.

Confidentiality Statement: This document contains trade secrets and proprietary or confidential business information and shall not be disclosed unless required by applicable laws or regulations. In any case, the subject of the disclosure shall be informed that such information is proprietary or confidential and shall not be further disclosed, copied or distributed to persons not participating in the clinical study.

Protocol Summary

**Title**	Clinical Study of Cord Blood-derived CAR-NK Cells Targeting CD19 in the Treatment of Refractory/Relapsed B-cell NHL
**Study Objectives**	Primary Objective<br>1) To observe the safety of cord blood-derived CAR-NK cells (CB CAR-NK019) targeting CD19 in the treatment of B-cell non-Hodgkin lymphoma, determine the maximum tolerated dose (MTD) of CB CAR-NK019 for B-cell non-Hodgkin lymphoma and a recommended expansion phase dose;<br>2) To evaluate the overall response rate (CR and PR);<br>Secondary Objective<br>1) Duration of response,<br>2) Overall survival and progression-free survival;<br>Exploratory:<br>1) Proliferation and persistence of CAR^+ NK cells in blood;<br>2) Levels of cytokines in peripheral blood;<br>3) The factors related to the efficacy.
**Sponsor**	The Second Affiliated Hospital, College of Medicine, Zhejiang University
**Enrolled Patients**	Relapsed/refractory B-cell non-Hodgkin lymphoma
**Study Design**	This is a single-center, open, single-arm, dose-escalation and expansion study to observe the safety and efficacy of cord blood-derived CAR NK cells targeting CD19 in patients with B-cell non-Hodgkin lymphoma.<br>The study will include two parts: **Part I** is a dose escalation phase designed according to the "3+3" dose escalation principle: 2.0, 3.0, and 4.0 × 10^6 CAR^+ NK cells per kg body weight. Each dose was infused once a week for 3 weeks. Three to six subjects are intended to be enrolled in each dose group, and each subject will be observed for at least 28 days and has a follow-up to observe

	the duration of responses. **Part II** is the dose-expansion phase: The objective of cohort expansion phase (Part II) is to confirm the safety and efficacy of the dose chosen in Part I. The recommended expansion part dose will be determined by Safety Review Committee based on a review of the safety efficacy, and proliferation of CB CAR-NK019 generated in Part I of the study. In expansion phase, up to approximately 30 subjects will be enrolled.<br>FC conditioning chemotherapy regimen: Fludarabine 30mg/m^2/ day, 4 days (d-6 ~ -3); cyclophosphamide (CTX) 750mg/m^2 day, 3 days (d-5, -4, -3).
**Number of Patients**	Part I is scheduled to enroll 9 to 18 participants, and Part II is scheduled to enroll 30 participants.
**Study Procedure**	In this study, subjects will undergo the following procedures:<br>(1) Providing written informed consent, medical history (including prior treatment for lymphoma), and disease history. Assessment of physical examination (including vital signs and physical status), neurological evaluation, physical examination including routine blood test, biochemical examination, cytokine, C-reactive protein, lymphocyte subtype test, etc.<br>(2) Fertile women will undergo urine or serum pregnancy tests.<br>(3) Subjects will also undergo baseline electrocardiography (ECG), echocardiography (ECHO), magnetic resonance imaging (MRI), B-ultrasound, and positron emission tomography-computed tomography (PET-CT).<br>(4) After completing the patient screening and meeting the enrollment criteria, the clinical investigator informed the biotherapy center laboratory to prepare CB CAR-NK019 cells for 10-14 days.<br>(5) The FC regimen was given at -6 to -3 days of before CB CAR-NK019 infusion.

	(6) The first infusion of CB CAR-NK019 was accomplished, and the second infusion cells were prepared in the laboratory.<br>(7) CB CAR-NK019 cells were re-infused in the second week.<br>(8) CB CAR-NK019 cells were infused in the third week.<br>During the study, concomitant medications and adverse events will be recorded and reported.
**Follow-up Plan**	1. Screening period<br>Including informed consent, demographic data, medical history, vital signs, physical examination, combined medication, pregnancy test, blood routine, serum biochemistry, electrocardiogram, bone marrow smear or biopsy, immune function test, imaging examination, etc.<br>2. Follow-up after infusion: follow-up on day 1, 4, 7, 10, 14, 21, and 28 (treatment period)<br>Including general information of subjects, ECOG score, symptoms and signs, blood routine, serum biochemistry, electrocardiogram, immune function test, imaging examination, etc. A bone marrow smear or biopsy should be obtained if bone marrow is involved. Peripheral blood was collected to detect the amplification of CAR.<br>3. Long-term follow-up after infusion. Every 3 months from month 3 to 1 year, and every 6 months for 1 year to 2 years.<br>Including general information of the subjects, ECOG score, symptoms and signs, blood routine examination, peripheral blood smear examination, electrocardiogram, serum biochemistry, immune function detection, imaging examination, etc. If there is bone marrow involvement, bone marrow smear or biopsy should be performed. Peripheral blood was collected to detect CAR amplification and B cell expression.
**Study Duration**	3 years
**Treatment Requirement**	1. Conditioning chemotherapy regimen: Fludarabine and cyclophosphamide<br>2. CB CAR-NK019 cell infusion solution

	3. All other treatments are determined by the investigator according to the subject's condition; Drugs and other therapies that treat complications or relieve symptoms of complications, such as antihypertensive drugs, antibacterials, antihistamines, etc.
**Inclusion Criteria**	Subjects must meet all the following criteria:<br><br>1. All subjects in this study can understand and are willing to sign the written informed consent;<br>2. Aged 18-75 years, male or female;<br>3. Histologically confirmed diffuse large B-cell lymphoma (DLBCL), transformed follicular lymphoma (TFL), primary mediastinal B-cell lymphoma (PMBCL), mantle cell lymphoma (MCL), and other transformed indolent lymphomas:<br>(1) Refractory or relapsed DLBCL: patients did not achieve complete remission after 2-line therapy; progression of the disease during any lines of treatment or a period of disease stabilization equal to or less than 6 months; or disease progression/relapse within 12 months after autologous hematopoietic stem cell transplantation;<br>(2) Refractory or relapsed MCL patients who must be resistant to or inability to tolerate BTK inhibitors;<br>(3) Refractory or relapsed indolent B-cell NHL patients with third-line therapy failure or recurrence;<br>(4) Prior treatment must include CD20 monoclonal antibody therapy (unless subjects are CD20-negative) and anthracyclines;<br>4. There was at least one measurable lesion with the longest diameter ≥ 1.5 cm;<br>5. Expected survival ≥ 12 weeks;<br>6. The expression of CD19 in tumor tissue biopsy was positive;<br>7. ECOG score 0-2;<br>8. Adequate organ function:

	(1) Alanine aminotransferase, Aspartate aminotransferase ≤ 2.5 × UNL (upper limit of normal);<br>(2) Creatinine clearance (Cockcroft-Gault method) ≥ 60 mL/min;<br>(3) Serum total bilirubin and alkaline phosphatase ≤ 1.5 × UNL;<br>(4) Glomerular filtration rate >50 mL/min<br>(5) Cardiac ejection fraction (EF) ≥ 50%;<br>(6) Basic oxygen saturation > 92% in an indoor natural air environment<br>9. One previous stem cell transplant is permitted<br>10. Approved anti-B-cell lymphoma therapies, such as systemic chemotherapy, whole-body radiotherapy, and immunotherapy, must have been stopped at least 3 weeks before enrollment;<br>11. Accept patients who have previously received CAR T cell therapy and failed to respond on 3 months evaluation or relapse;<br>12. Female subjects with childbearing potential must have a negative pregnancy test and agree to use effective contraception during the study;<br>13. Subjects should have negative tests for coronavirus covid-19.
**Exclusion Criteria**	Subjects who meet any of the following criteria will not allowed in this study:<br>1. Allergic to any of the components of cell products;<br>2. History of other tumors;<br>3. Acute GvHD or extensive chronic GvHD with grade II-IV (Glucksberg standard) in the past or are receiving anti-GVHD treatment;<br>4. Had received gene therapy within the past 3 months;<br>5. Active infections requiring treatment (except for simple urinary tract infections, bacterial pharyngitis); however, prophylactic antibiotics, antiviral and antifungal infection treatment are permitted;<br>6. Patents infected with hepatitis B (HBsAg positive, but HBV-DNA < 10^3 is not excluded) or hepatitis C virus (including virus carriers), syphilis and other acquired and congenital immunodeficiency diseases, including but not limited to HIV-infected persons;

	7. Subjects with Grade III or IV cardiac dysfunction according to the New York Heart Association's cardiac function grading criteria;<br>8. Patients who received antitumor therapy earlier but did not recover from the toxicity (CTCAE 5.0 toxicity did not recover to ≤ grade 1, except fatigue, anorexia, alopecia);<br>9. Subjects with a history of epilepsy or other central nervous system disorders;<br>10. Head-enhanced CT or MRI showing evidence of central nervous system lymphoma;<br>11. Lactating women who are unwilling to stop breastfeeding;<br>12. Any other factors that the investigator believes may increase the risk to the subject or interfere with the test results.
**Study Withdrawal**	1. Withdrawal decided by the investigator<br>The enrolled subjects have the following conditions during the study that are not suitable for further continue in the study, and the investigator will decide to withdraw the case from the study:<br>1) Patients who are severely allergic to the components of the investigational drug in this study;<br>2) Adverse events occur, and the investigator determines that it is not appropriate to continue the study;<br>3) Poor compliance, unable to comply with the protocol;<br>4) Other situations in which the researcher considers it inappropriate to continue the study.<br>2. Subjects voluntarily withdrew from the study<br>1) Subjects or their family members refuse to continue participating in the clinical study and withdraw from it to their doctor.<br>2) Loss of follow-up.
**Criteria of Cord Blood**	The umbilical cord blood was provided by Zhejiang Province Blood Center Cord Blood Bank.<br>Meet the following criteria:<br>1. Signed written informed consent;<br>2. Pathogen detection: HIV, HBV (surface antigen and core antibody), HCV,

	syphilis, CMV, EBV, HTLV and HPV B19 test results should be negative;<br>3. Nose/throat swab nucleic acid test of coronavirus by RT-PCR was negative;
**Exclusion Criteria of Cord Blood**	The following virological tests were positive:<br>➢ HIV antibody;<br>➢ HCV antibody;<br>➢ Hepatitis B virus surface antigen (HbsAg);<br>➢ Hepatitis B virus core antibody;<br>➢ Treponema pallidum antibody;<br>➢ EB virus antibodies;<br>➢ Antibodies to cytomegalovirus<br>➢ Coronavirus covid-19 nucleic acid
**Study Procedure**	This trial was divided into screening period, conditioning chemotherapy period, treatment and DLT observation period, long-term follow-up period. After screening, eligible subjects will be screened at -20 days before receiving CAR-NK019 infusion, preparing CAR-NK019 at -14 days, and chemotherapy from -6 to -3 days. The conditioning chemotherapy is as follows:<br>➢ Fludarabine 30 mg/m^2/d, intravenously for 4 days;<br>➢ Cyclophosphamide 750 mg/m^2/d, intravenous infusion for 3 days;<br>After chemotherapy, the patient will receive a single dose of CAR-NK019 intravenous infusion once a week for 3 weeks. The DLT observation period was 28 days after infusion, and the adverse events were observed. Then, a long-term follow-up was conducted.
**Study Endpoint**	<u>Primary study endpoints:</u><br>➢ Incidence of dose-limiting toxicity (DLT) in subjects treated with CB CAR-NK019 injection<br>Secondary study endpoint<br>➢ Efficacy of the subjects treated with CB CAR-NK019, including overall survival (OS), progression-free survival (PFS), complete response (CR), partial response (PR), objective response rate (ORR), and duration of response (DOR);
**Statistical Analysis**	Statistical analysis was performed using SAS® version 9.2. Unless otherwise specified, the measurement data are generally described by the number of cases, mean, standard deviation, median, minimum and maximum values. Enumeration data are generally described by case numbers and percentage.

	The Kaplan-Meier method was used to estimate the follow up time for survival, PFS, and DOR. Its overall 95% confidence interval for time-event data.<br>Baseline evaluation<br>Demographic variables and baseline characteristics (e.g., sex, age, weight, disease category, drug concomitant, past treatment history, vital signs, etc.) were summarized by dose group.<br>Safety evaluation<br>Safety statistical analysis included descriptive statistics for adverse events, vital signs, laboratory test values, dose-limiting toxicity (DLT), and electrocardiogram (ECG).<br>Effectiveness evaluation<br>Statistical efficacy measures evaluated by the investigators included OS, PFS, CR, PR, SD and PD. ORR defined as CR + PR assessed using Lugano 2014 criteria. Kaplan-Meier curves were used to describe OS based on the full analysis set, and the median OS, the first and third quartiles were summarized and described. The number and percentage of other non-time event efficacy indicators were summarized and described.<br>Analysis set<br>Modified Intention-to-Treat Analysis Set (mITT): The mITT analysis set included all subjects enrolled and treated with a CD19 CAR-NK target dose. This analysis set will be used for the analysis of safety (exception of MTD) and efficacy;<br>The MTD Analysis Set: All subjects in Part I study who received CAR-NK cell infusion and either completed 28-days of follow-up, or who had a DLT;<br>Full Analysis Set (FAS): All participants who have used study drugs will be included in this analysis set. FAS sets are primarily used for baseline and validity analysis.

	Safety Analysis Set (SS) : Included all subjects who had received at least one study drug treatment;<br>Data Analysis<br>This study included two phases. Part I is dose-escalation phase, which plan to enroll 9 to 18 cases. End of this phase study, primary safety and efficacy will be analyzed and reported. Part II is expansion phase study that plan to enroll 30 cases. End of Part II study, the data from all subjects enrolled in two phase study will be analyzed for safety and efficacy.

*C*atalog

Investigator's statement......13

1 Background......14

1.1 CAR-NK cell therapy......14

1.2 Source of CAR-NK cells......15

1.3 Results of preclinical study of CAR-NK019 cells......15

2. Objectives......22

2.1 Primary objective:......22

2.2 Secondary objective:......22

2.3 Exploratory:......22

3. Study Overview......22

3.1 Study design......22

3.2 Overall research procedures......23

3.3 Dose of CAR-NK cells......24

3.3.1 Dose escalation......24

3.3.2 Dose of expansion study......24

3.3.3 Maximum Tolerated Dose......24

3.3.4 Dose Limited Toxicity......25

3.4 Study population......25

3.4.1 Inclusion criteria......25

3.4.2 Exclusion Criteria......27

3.4.3 Study Withdrawal......28

3.4.3.1 Withdrawal by investigator......28

3.4.3.2 Withdrawal by participant......28

3.4.4 Criteria for donor of cord blood......28

3.4.4.1 Inclusion criteria for donor of cord blood......28

3.4.4.2 Exclusion criteria for donor of cord blood......28

3.5 Investigational drugs......29

3.6 Study procedures and related examinations......30

3.6.1 Screening......30

3.6.2 Generation and quality control of CAR-NK cells......31

3.6.3 CAR NK cells Infusion......33

3.6.4 Shipment and handover of cord blood and CAR-NK cell product......35

3.6.5 Quality standard and quality control report......36

3.6.6 Conditioning Chemotherapy......37

3.6.7 CAR-NK019 Treatment 37
3.6.8 Post-treatment evaluation period 39
3.6.9 Long-term follow-up period 40

3.7 Endpoint Evaluation 40
3.7.1 Safety Endpoint Evaluation 40
3.7.2 Efficacy Endpoint Evaluation 40

3.8 Concomitant medication and treatment 41

3.9 Research procedure 42

FLOW CHART 42

4. Sample Size Considerations 45

4.1 Part I sample size calculation 45

4.2 Part II sample size calculation 45

5. Statistical Analysis 46

5.1 Analysis set 47

5.2 Data Analysis 47

6. Data management and confidentiality 47

6.1 Data collection and management 47

6.2 Data confidentiality 48

7. Informed consent 48

8. Observation of adverse events 49

8.1 Definition of Adverse Events 49
8.1.1 Definitions 49
8.1.2 Grades 49

8.2 Risk-prevention and treatment 49
8.2.1 Cytokine Release Syndrome 49
8.2.2 Hypotension and renal insufficiency 55
8.2.3 Cardiac toxicity 55
8.2.4 Hemophilic lymphohistiocytosis 55
8.2.5 Neurotoxicity 56
8.2.6 Fever and neutropenia 61
8.2.7 Infection prevention 61
8.2.8 B cell aplasia 61
8.2.9 Tumor lysis syndrome 61
8.2.10 Preventing Deep Vein Thrombosis 61

8.3 Recording and reporting of adverse events 62

Investigator's statement

1. I will personally participate in and lead this clinical study.

2. I have read and approved the clinical trial protocol, and the study will be conducted following the ethical and scientific principles stipulated by the Declaration of Helsinki and the GCP. I agree to carry out this clinical study under the design and provisions of this protocol.

3. According to the requirements of GCP, I will ensure that all patients sign written informed consent before entering the study.

4. I will be responsible for making medical decisions related to clinical trials, ensuring that patients receive timely and appropriate treatment when adverse events occur during the trials, and recording and reporting serious adverse events in accordance with relevant national regulations.

5. I guarantee that the data will be true, accurate, complete, and loaded into CRF in a timely manner.

6. I promise to keep patient information and related matters confidential.

Principal Investigator: Professor Wenbin Qian	Signature:
Research institution: The Second Affiliated Hospital, College of Medicine, Zhejiang University	Date：2022-6-16

1 Background

Relapsed/Refractory (R/R) B-cell lymphoma currently lacks effective, safe, and resistance-to-relapse therapeutic strategies. CD19-specific chimeric antigen receptor (CAR)-T cells have demonstrated remarkable efficacy in treating R/R B-cell malignancy. Recently, multiple clinical trials have reported their survival data over two years, indicating that the overall response rate (ORR) of patients with R/R large B-cell lymphoma treated with CD19 CAR-T is 44-91%, and the complete response rate (CRR) is 28-68%. However, these results also reveal that more than half of patients either did not respond or relapse after remission. Moreover, several inherent obstacles limit the broader application of CAR-T cells. These include the high cost of the treatment, insufficient number or poor-quality T cells from intensively treated patients resulting in defective CAR-T infusion products, and the extended time required to prepare the CAR-T products on schedule. Furthermore, substantial toxic effects, including cytokine release syndrome (CRS) and neurotoxicity, present additional hurdles.

Natural killer (NK) cells, known for the potent cytotoxicity against tumor cells, are emerging as promising alternatives to T cells in CAR-based cancer immunotherapy. These cells can be obtained from various sources. NK cells engineered to express a CAR (CAR-NK) can circumvent several limitations of CAR-T cell therapy. Notably, CAR-NK cells have advantages over CAR-T cells, such as reduced risks of CRS, neurotoxicity, and graft-versus-host disease (GvHD), which are common concerns when using allogenic CAR-T cells.

1.1 CAR-NK cell therapy

Research has demonstrated that NK cells only initiate a killing response when inhibitory signals of KIRs are absent, such as the reduction of HLA Class I molecules in tumor cells or the mismatch between allogeneic NK cells' KIRs and recipient cells' HLA Class I molecules. However, NK cells will not respond if the target cells fail to provide ligand stimulation to activate receptors like NKG2D. This feature explains why allogeneic NK cells are safe, but tumor cells can also exploit it to suppress the activation signal to evade the immune response. Nevertheless, CAR-NK cells can overcome this limitation by disrupting the inactive state of NK cells and allowing them to selectively identify and eliminate target cells selectively. Compared to CAR-T cells, CAR-NK cells offer several immunological advantages. Firstly, allogeneic NK cells do not induce GvHD, making them a preferred choice for allogeneic immune cell adoptive therapy. Secondly, NK cells directly eliminate cells by releasing granzyme, perforin, TNF-α, and other cytokines but do not secrete IL-1 or IL-6, which minimizes the risk of CRS. Thirdly, NK cells surpass T cells to persist in killing target cells, with a single activated NK cell that can continuously eliminate up to 10 tumor cells. Fourthly, apart from CAR-mediated specific killing, allogeneic NK cells can also engage in the multi-target cytotoxicity through mechanisms of KIRs mismatch to reduce antigen escape. Lastly, NK cells can bind to antibody Fc fragments via CD16, enabling specific clearance of target cells through

antibody-dependent cellular cytotoxicity.

1.2 Source of CAR-NK cells

NK cells used in CAR-NK research can be derived from various sources, such as NK92 cell line, peripheral blood mononuclear cells (PBMC), umbilical cord blood (UCB), $CD34^{+}$ hematopoietic progenitor cells, and induced pluripotent stem cells (iPSCs). The NK92 cell line, favored for its low cost of manufacturing and ease of use, has several inherent drawbacks, such as the risk of inducing a “second tumor”, absence of CD16 and NKp44 expression, and the incapacity to proliferate due to pretreatment with radiation. In contrast, PBMCs from healthy donors are a crucial source of primary NK cells and have been extensively used in clinical trials. A high quantity of NK cells can be isolated from PBMCs using the commercialized NK cell isolation kit and then stimulated and expanded in a cytokine manner. UCB is another appealing source, with a higher potential for proliferation than PBMC-derived NK cells due to its stem cell properties. Furthermore, establishing cord blood banks facilitates the selection of donor NK cells based on specific HLA subtypes. A notable study by Katayoun Rezvani's group, published in the New England Journal of Medicine in 2020, used UCB-derived CD19 CAR-NK cells to treat 11 patients with chronic lymphocytic leukemia or relapsed/refractory non-Hodgkin lymphoma, obtaining the overall response rate of 73% and a complete response rate of 63%. Most significantly, this trial had no grades of CRS, neurotoxicity, or other inflammatory events. This clinical trial offers a profound foundation for further research.

In this study, we developed a UCB-derived CD19 CAR-NK cell that secreted IL-15 (CAR-NK019), significantly improving the proliferation and maintenance of CAR-NK cells in vivo. We used an alternative baboon envelope pseudotyped lentiviral vectors (BaEV-LV) to increase the transduction capacity to generate CAR-NK cells. Preclinical research has demonstrated that CAR-NK019 can achieve up to 1000-fold amplification in vitro and exhibit robust CD19-positive cells killing capabilities in vitro and mouse models. Therefore, we plan to initiate a single-center, open-label, single-arm Phase I clinic trial to assess the safety and efficacy of CAR-NK019 in patients with refractory/relapsed B-cell non-Hodgkin lymphoma.

1.3 Results of preclinical study of CAR-NK019 cells

1.3.1 Baboon endogenous virus envelope pseudotyped lentivirus significantly improves the transduction effect on NK cells

The lentivirus system is a safe and efficient technology for manufacturing CAR-T cells in vitro. In this study, we initially employed the third-generation lentiviral package system, which includes the pLenti EF1a expression vector, two packaging plasmids (pLP1, pLP2), and an envelope plasmid (pVSV-G) for generating CAR-NK cells. However, both our research and that of others identified that the conventional lentiviral particles pseudotyped by VSV-G are inefficient in infecting NK cells. Thus, we constructed a baboon endogenous virus (BaEV) envelope pseudotyped lentivirus to

increase the transduction efficacy (Figure 1A). As depicted in Figures B and C, we successfully generated NK cells expressing fluorescent protein Venus in NK92 cell line and PBMCs (Figure 1D). Moreover, lentiviral particles produced with BaEV pseudo-enveloped packaging system achieved a high titer of 1-5 × 10^8 TU/mL.

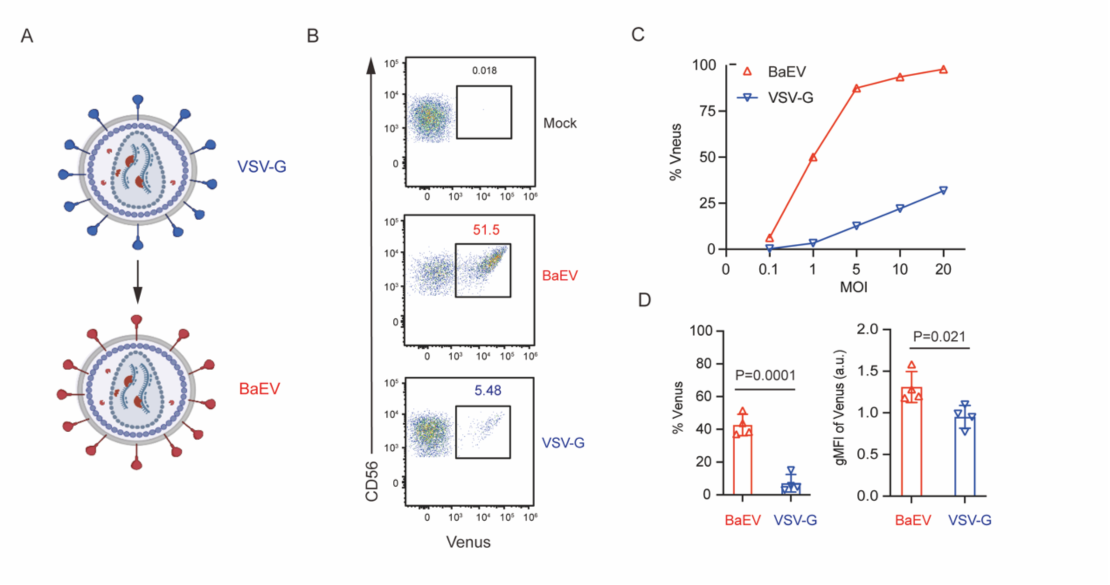

Figure 1. Baboon Endogenous Virus pseudotyped lentivirus for the transduction of NK cells

1.3.2 Production of CAR-NK cells

The CAR-expressing lentiviral vector, CAR-NK019, is shown in Figure 2A. The CAR structure composed an anti-CD19 single-chain antibody variable region (FMC63, Genebank: HM852952.1), CD8a hinge region, CD8a transmembrane region, 4-1BB costimulatory molecule, and CD3zeta intracellular region, and could tandemly express human IL-15 (UniProtKB: P40933-1) via a Furin-SGSG-T2A peptide. In this study, the NK cells were derived from UCB, necessitating the depletion of $CD3^+$ T cells to prevent GvHD. The residual of $CD3^+$T cells during the manufacture were monitored using flow cytometry (Figure 2B). The expansion of CARN-K cells were tracked, showing a 1000-fold amplification within 14 days (Figure 2C). High transduction efficiency of CAR-NK cells was achieved using the BaEV pseudo-enveloped lentivirus (Figure 2D, E). The generated CAR-NK cells extensively expressed NK cell-activated receptors such as NKG2D, 2B4, and metabolism-associated membrane transport proteins such as CD71 and CD98, as detected by CyTOF assay (Figure 2F, G).

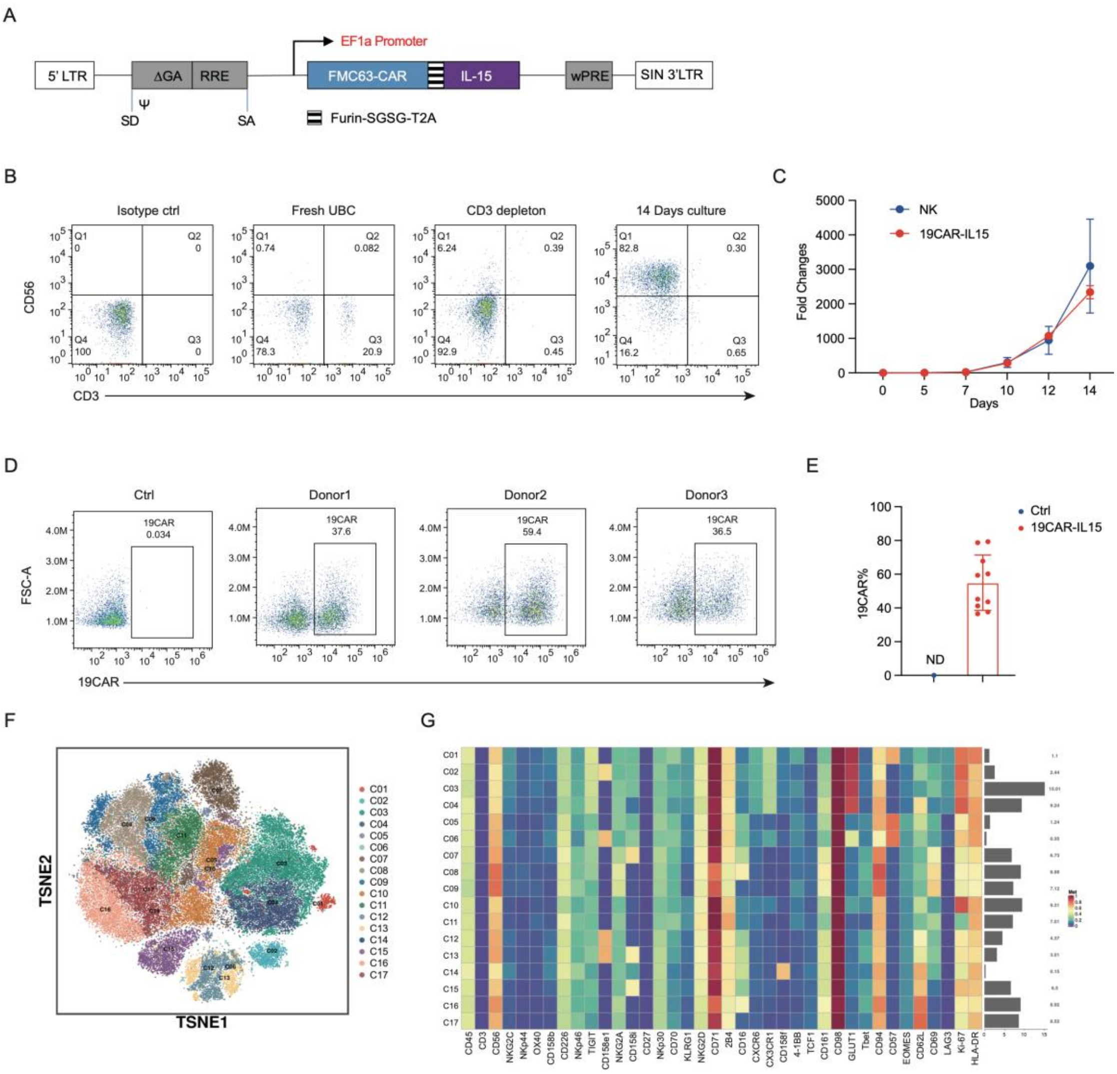

Figure 2. Generation and evaluation of CAR-NK019 cells

1.3.3 The cytotoxicity of CAR-NK in vitro

To assess the cytotoxicity of CAR-NK019 against the B cell lymphoma cells, we co-incubated CAR-NK cells with CD19-positive Raji and JeKo-1 cells at various Effect-to-Target ratios. The luciferase-based cytotoxicity assay revealed that CAR-NK cells were more effective in eradicating Raji and JeKo-1 cells compared to regular NK cells (Figure 3A, B). Further real-time cell analysis (RTCA) analysis confirmed that CAR-NK cells rapidly and dose-dependently destroyed CD19-positive tumor cells (Figure 3C). In addition, we observed that the specific tumor-killing activity of CAR-NK cells in response to CD19 antigen exposure was associated with enhanced CD107α degranulation activity (Figure 3D, E). These findings demonstrated that CAR-NK019 exhibited a specific antitumor activity in vitro.

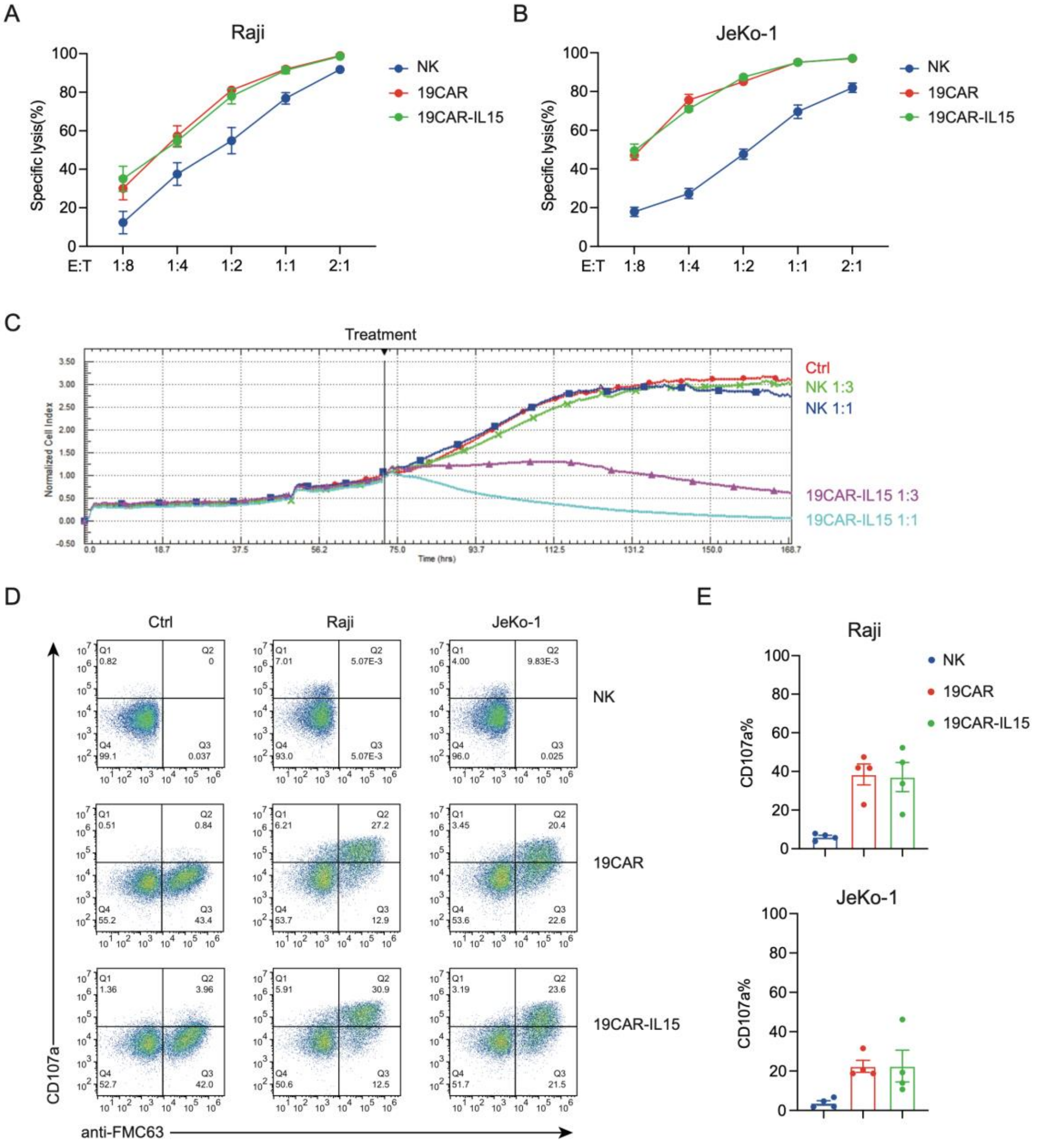

Figure 3. CAR-NK019 exhibits a CD19 antigen-specific cytotoxicity

1.3.4 The anti-lymphoma function of CAR-NK019 in vivo

Next, we investigated the anti-tumor efficacy of CAR-NK019 in vivo using a JeKo-1-Luc-bearing NSG mice model. As shown in Figure 4A-C, CAR-NK019 significantly reduced tumor burden and prolonged survival. To assess the persistence and infiltration of CAR-NK cells in vivo, we analyzed the CAR^{-}NK cells in blood, spleen, and bone marrow on day 68 post-infusion using flow cytometry. The results revealed a substantial presence of human NK cells in the mice's bone marrow, peripheral blood, and spleen, with the majority being CAR-positive (Figure 5A, B). This suggested that CAR-NK cells were proliferating specifically in vivo. Moreover, human IL-15 was detected in peripheral blood in mice by the ELISA assay (Figure 5C). These findings demonstrate that CAR-NK019 can effectively eradicate B-cell lymphomas in the xenograft mice model and maintain persistence in vivo.

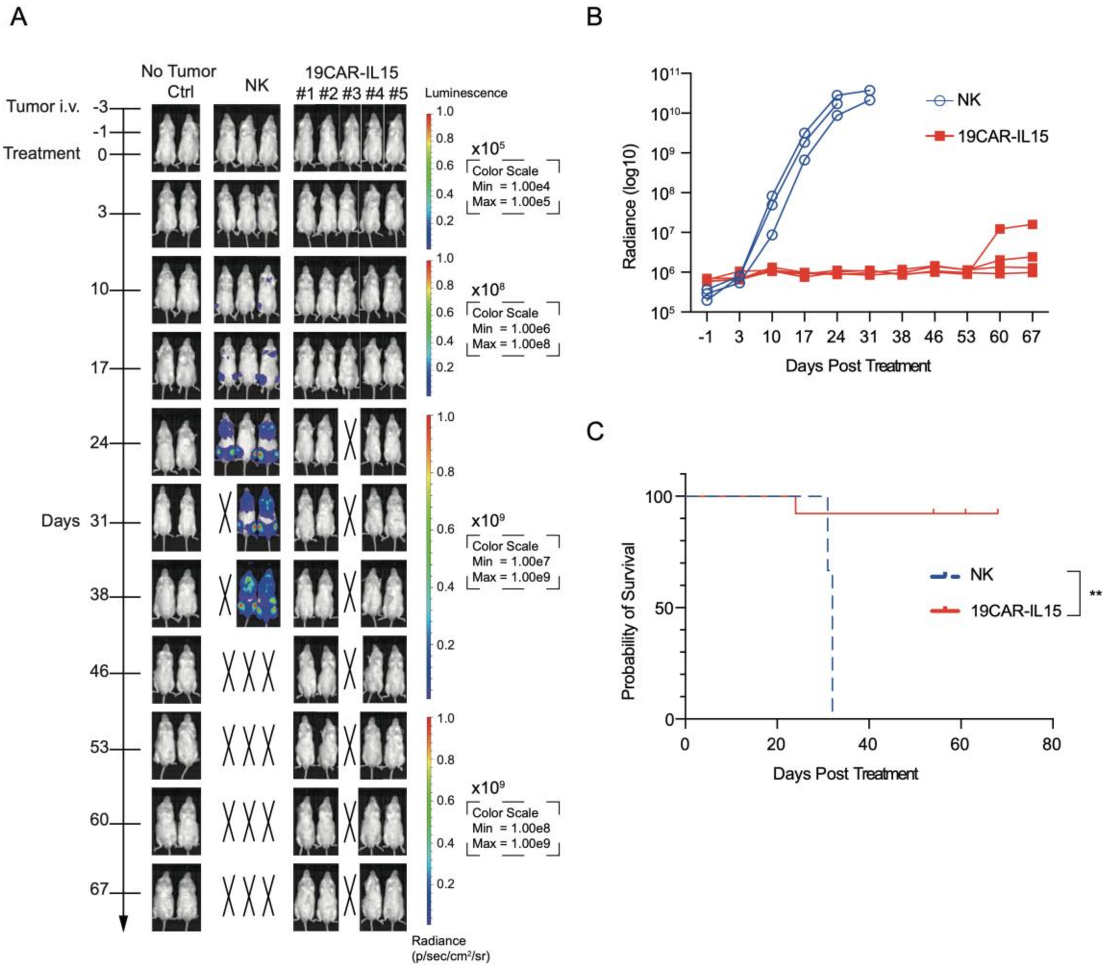

Figure 4. In vivo anti-B-cell lymphoma efficacy of CAR-NK019 cells

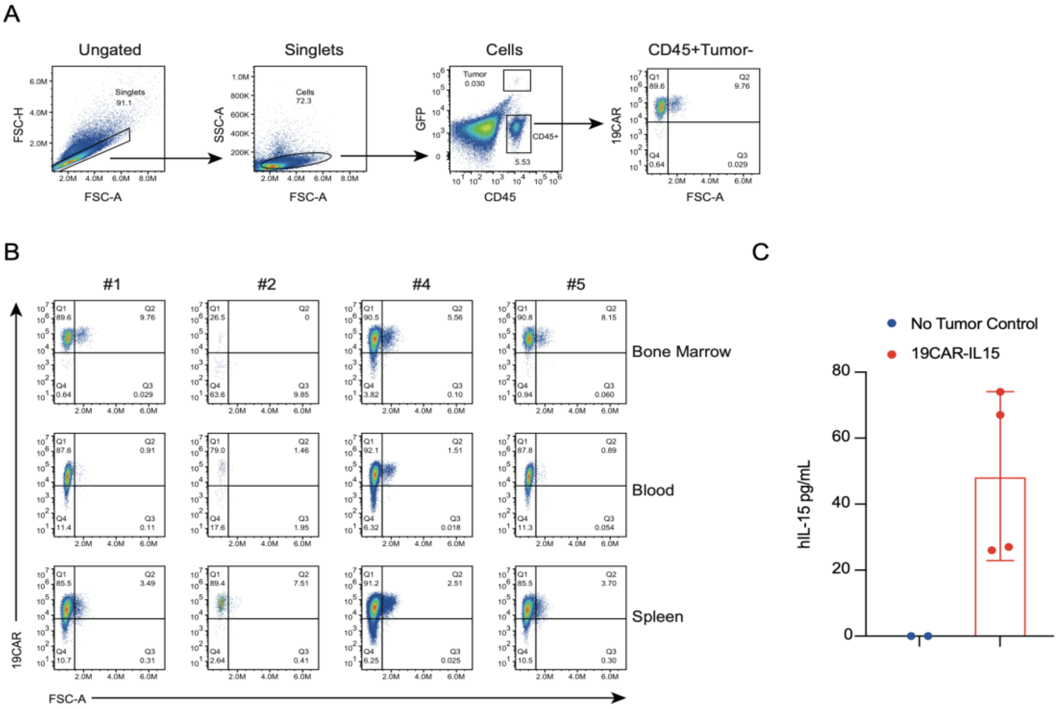

Figure 5. Long-term persistence of CAR-NK019 in mice

1.3.5 Assessment of the tumorigenicity of CAR-NK019

BALB/c mice (male and female) were used to assess the tumorigenicity of CAR-NK019 in vivo. Two mice models with 2×10^6 and 4×10^6 of CAR-NK019 models were established, respectively, and the murine colon cancer cell line CT-26 was injected subcutaneously as a positive control for tumorigenesis. The mice injected with CT-26 showed tumorigenesis on day 7, and the average tumor volume was up to 3500 mm^3. All the mice in the CT-26 group succumbed to the tumor burden on day 25. In contrast, there was no tumorigenicity sign in PBS and both CAR-NK dose-escalated groups (Figure 6A, B, C). We also monitored the body weight changes among CAR-NK, CT-26, and PBS groups. The results indicated that both male and female mice injected with CAR-NK019 maintained a body weight comparable to the PBS group, and no visible subcutaneous tumors were detected in any of the CAR-NK019 injected groups (Figure 6D, E).

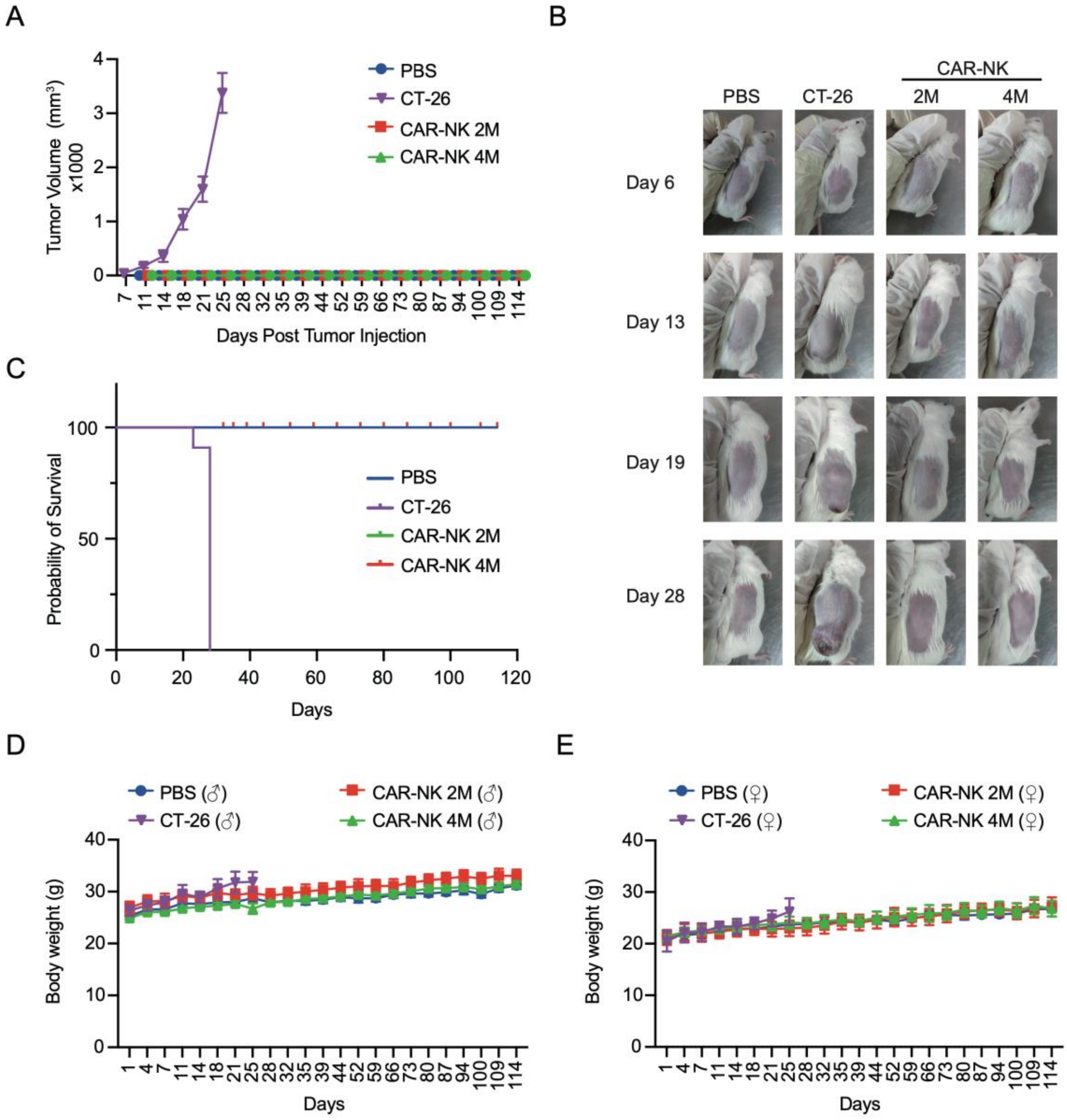

Figure 6. Evaluation of the tumorigenicity of CAR-NK019

2. Objectives

2.1 Primary objective:

1) To observe the safety of cord blood-derived CAR-NK cells (CB CAR-NK019) targeting CD19 in the treatment of B-cell non-Hodgkin lymphoma, determine the maximum tolerated dose (MTD) of CB CAR-NK019 for B-cell non-Hodgkin lymphoma and a recommended expansion phase dose;

2) To evaluate the overall response rate (CR and PR);

2.2 Secondary objective:

1) Duration of response,

2) Overall survival and progression-free survival;

2.3 Exploratory:

1) Proliferation and persistence of CAR+ NK cells in blood;

2) Levels of cytokines in peripheral blood;

3) The factors related to the efficacy.

3. Study Overview

3.1 Study design

This is dose-escalation and expansion phase of a single-center, open, single-arm clinical trial to evaluate the safety and efficacy of CB CAR-NK019, a CD19 target CAR-NK cells, in subjects with refractory/relapsed B-cell non-Hodgkin lymphoma.

This trial is divided into two phases. Phase I: dose escalation phase, with 9 to 18 participants planned to enroll; Phase II: dose expansion phase, with 30 participants planned to enroll. Every subject will undergo the following procedure: screening period, CAR-NK cell preparation, baseline period, conditioning chemotherapy, treatment with CB CAR-NK019 cells, post-treatment assessment, and long-term follow-up.

3.2 Overall research procedures

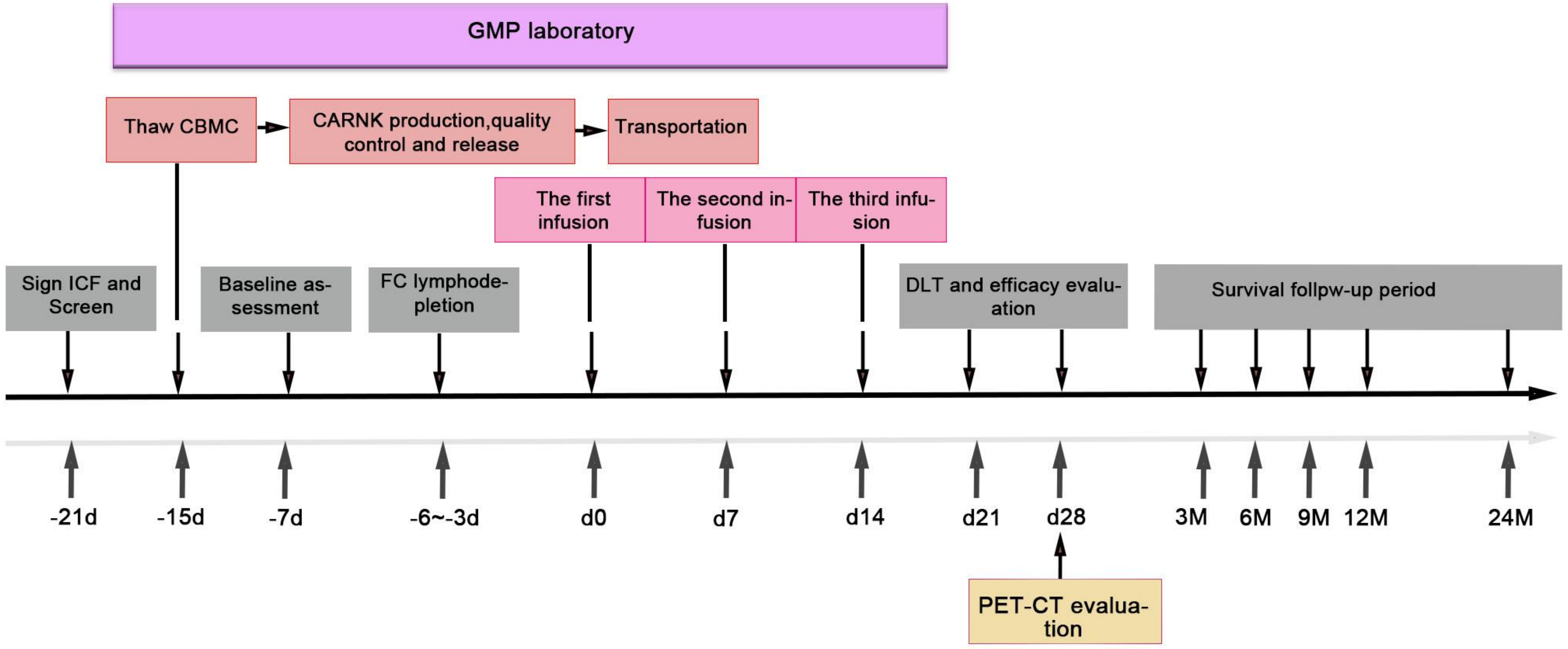

3.3 Dose of CAR-NK cells

3.3.1 Dose escalation

The **part I** study plans to enroll 9-18 patients in the dose escalation phase. Subjects will receive three infusions of CAR-NK019 cells (once weekly for three weeks) using the "3+3" dose escalation principle. Three dose levels were used: level 1 (2×10^6/kg), level 2 (3×10^6/kg), and level 3 (4×10^6/kg). Three to six subjects are planed to be enrolled for each dose level, and each subject will be observed for at least 28 days after receiving the last infusion of a single course, with a long-term follow-up period of 2 years after the first infusion.

"3+3" dose escalation profile: if more than 2 of 3 subjects in level 1 develop DLT, a new dose group 1×10^6/kg (level -1) will be added to the trial. If DLT occurs in more than 2 out of 3 subjects or 2 out of 6 subjects in level 1, the clinical study will be terminated. If none of the three subjects showed DLT in any dose levels, they were moved to the next dose level. If 1 out of 3 subjects in a dose level has DLT, 3 more subjects in the dose level are required to complete the tolerance observation of the dose. If the newly added 3 subjects no longer have DLT, they can enter the next dose level. If one or more of the three additional subjects develop DLT, the dose escalation experiment is discontinued, and the previous dose of this dose is defined as MTD. If more than 2 out of 3 subjects in a dose level develop DLT, the dose escalation is immediately terminated, and 3 additional subjects are added to the previous dose level, and if only 1 out of 6 patients in the additional 3 subjects in this dose level develops DLT, the dose level is defined as MTD.

To protect the subject's safety, each subject in the same dose level must wait at least 14 days before treatment the next subject. The last subject in each dose level must complete DLT observation at least 28 days before the following dose level subjects are enrolled and started to be treated.

3.3.2 Dose of expansion study

The recommended dose in expansion study (Part II) will be determined by Safety Review Committee based on a review of the safety efficacy, and proliferation of CB CAR-NK019 generated in Part I of the study. In the expansion phase, up to approximately 30 subjects will be enrolled.

3.3.3 Maximum Tolerated Dose

Maximum Tolerated Dose (MTD) was the maximum dose given to less than two out of six subjects. To determine the MTD dose level, at least 5 evaluable subjects with no DLT report or 1 in 6 evaluable subjects must have experienced DLT.

3.3.4 Dose Limited Toxicity

All adverse events are graded according to the latest CTCAE5.0 version, and any of the following adverse events that occur within 28 days after CB CAR-NK019 infusion that are "likely" and "definitely" related to treatment will be considered Dose Limited Toxicity (DLT):

Non-hematological toxicity: ≥ Grade 3 toxicity occurs, except for toxicity as defined below:

- Nausea, vomiting, and diarrhea that resolves to grade 1 or less or baseline within 7 days;
- Infection, fever (including febrile neutropenia), electrolyte disturbance, remission to grade 1 or less or baseline within 7 days;
- Grade 3 elevated aminotransferase, alkaline phosphatase, bilirubin, or other abnormal liver function, returning to grade 2 or below within 14 days;
- Grade 3 fever;
- Drug-related hypersensitivity occurs within 2 hours after receiving CAR-NK019 infusion and reverses to grade 2 or below within 24 hours with standard therapy;
- hypogammaglobulinemia;

Hematological toxicity: ≥ grade 4 toxicity, lasting more than 28 days

Any level of toxicity determined by the investigator will result in permanent discontinuation;

Other toxicities: Any level of other toxicities that the Data Safety Oversight Board (DSMC) determines cause the subject to withdraw from the study, and the evaluation determines DLT.

3.4 Study population

3.4.1 Inclusion criteria

Subjects must meet all of the following criteria:

1. All subjects in this study can understand and are willing to sign the written informed consent;

2. Aged 18-75 years, male or female;

3. Histologically confirmed diffuse large B-cell lymphoma (DLBCL), transformed follicular lymphoma (TFL), primary mediastinal B-cell lymphoma (PMBCL), mantle cell lymphoma (MCL), and other transformed indolent lymphomas:

(1) Refractory or relapsed DLBCL: patients did not achieve complete remission after 2-line therapy; progression of the disease during any lines of treatment or a period of disease stabilization equal to or less than 6 months; or disease progression/relapse within 12 months after autologous hematopoietic stem cell transplantation;

(2) Refractory or relapsed MCL patients who must be resistant to or inability to tolerate BTK inhibitors;

(3) Refractory or relapsed indolent B-cell NHL patients with third-line therapy failure or recurrence;

(4) Prior treatment must include CD20 monoclonal antibody therapy (unless subjects are CD20-negative) and anthracyclines;

4. There was at least one measurable lesion with the longest diameter ≥ 1.5 cm;

5. Expected survival ≥ 12 weeks;

6. The expression of CD19 in tumor tissue biopsy was positive;

7. ECOG score 0-2;

8. Adequate organ function:

(1) Alanine aminotransferase, Aspartate aminotransferase ≤ 2.5 × UNL (upper limit of normal);

(2) Creatinine clearance (Cockcroft-Gault method) ≥ 60 mL/min;

(3) Serum total bilirubin and alkaline phosphatase ≤ 1.5 × UNL;

(4) Glomerular filtration rate >50 mL/min

(5) Cardiac ejection fraction (EF) ≥ 50%;

(6) Basic oxygen saturation > 92% in an indoor natural air environment

9. One previous stem cell transplant is permitted

10. Approved anti-B-cell lymphoma therapies, such as systemic chemotherapy, whole-body radiotherapy, and immunotherapy, must have been stopped at least 3 weeks before enrollment;

11. Accept patients who have previously received CAR T cell therapy and failed to respond on 3 months evaluation or relapse;

12. Female subjects with childbearing potential must have a negative pregnancy test and agree to use effective contraception during the study;

13. Subjects should have negative tests for coronavirus covid-19.

3.4.2 Exclusion Criteria

1. Allergic to any of the components of cell products;

2. History of other tumors;

3. Acute GvHD or extensive chronic GvHD with grade II-IV (Glucksberg standard) in the past or are receiving anti-GVHD treatment;

4. Had received gene therapy within the past 3 months;

5. Active infections requiring treatment (except for simple urinary tract infections, bacterial pharyngitis); however, prophylactic antibiotics, antiviral and antifungal infection treatment are permitted;

6. Patents infected with hepatitis B (HBsAg positive, but HBV-DNA < 10^3 is not excluded) or hepatitis C virus (including virus carriers), syphilis and other acquired and congenital immunodeficiency diseases, including but not limited to HIV-infected;

7. Subjects with Grade III or IV cardiac dysfunction according to the New York Heart Association's cardiac function grading criteria;

8. Patients who received antitumor therapy earlier but did not recover from the toxicity (CTCAE 5.0 toxicity did not recover to ≤ grade 1, except fatigue, anorexia, alopecia);

9. Subjects with a history of epilepsy or other central nervous system disorders;

10. Head-enhanced CT or MRI showing evidence of central nervous system lymphoma;

11. Lactating women who are unwilling to stop breastfeeding;

12. Any other factors that the investigator believes may increase the risk to the subject or interfere with the test results.

3.4.3 Study Withdrawal

3.4.3.1 Withdrawal by investigator

The enrolled subjects have the following conditions during the study that are not suitable for further continue in the study, and the investigator will decide to withdraw the case from the study:
1) Patients who are severely allergic to the components of the investigational drug in this study;
2) Adverse events occur, and the investigator determines that it is not appropriate to continue the study;
3) Poor compliance, unable to comply with the study protocol;
4) Other situations in which the researcher considers it inappropriate to continue the study.

3.4.3.2 Withdrawal by participant

1) Subjects or their family members refuse to continue participating in the clinical study and withdraw from it to their doctor.

2) Loss of follow-up.

3.4.4 Criteria for donor of cord blood

3.4.4.1 Inclusion criteria for donor of cord blood

The umbilical cord blood was provided by Zhejiang Province Blood Center Cord Blood Bank.

Meet the following criteria:

1. Signed written informed consent;

2. Pathogen detection: HIV, HBV (surface antigen and core antibody), HCV, syphilis, CMV,

EBV, HTLV and HPV B19 test results should be negative;

3. Nose/throat swab nucleic acid test of coronavirus by RT-PCR was negative.

3.4.4.2 Exclusion criteria for donor of cord blood

The following virological tests were positive:

- HIV antibody;
- HCV antibody;
- Hepatitis B virus surface antigen (HbsAg);
- Hepatitis B virus core antibody;
- Treponema pallidum antibody;
- EB virus antibodies;
- Antibodies to cytomegalovirus

- Coronavirus covid-19 nucleic acid

3.5 Investigational drugs

(1) Investigational drug: CB CAR-NK019 injection
(2) Dosage form: injection
(3) Dose: CB CAR-NK019 cells were escalated from low dose to high dose according to the "3+3" dose-escalation principle in the escalation phase study; and the recommended expansion part dose will be determined by Safety Review Committee based on a review of the safety, efficacy, and proliferation of CB CAR-NK019 generated in Part I of the study.
(4) Administration: Intravenous infusion of CB CAR-NK019 cells: three infusions, once a week for three weeks.
(5) Preparation of CB CAR-NK019 cells: Human umbilical cord blood (CB） units were provided by Zhejiang Province Blood Center Cord Blood Bank. CAR-NK cell manufacture was performed in the GMP Laboratory of the Second Affiliated Hospital, College of Medicine, Zhejiang University. CB mononuclear cells (CBMC) were isolated by Ficoll density gradient centrifugation, and the T cell was depleted using CD3 microbeads (Militenyi Biotec), according to the manufacturer. The CD3 negative CBMCs were packaged and stored in liquid nitrogen to establish NK Seed Cell Bank (NK-SCB) of the Second Affiliated Hospital, College of Medicine, Zhejiang University. For CAR-NK cell manufacture, frozen CD3 negative CBMCs obtained from NK-SCB were thawed and stimulated with lethally irradiated K562 feeder cells expressing membrane-bound interleukin (IL)-21 and 41-BB ligand at a 2:1 ratio. Cells were maintained in OpTmizer ™CTS™ T-Cell Expansion SFM basic Medium (Gibco) with 2 mmol/L GlutaMAX (Thermo Fisher), 10% Human AB serum (Sigma,) and 1000 IU/mL human IL-2 (Quangang Pharmaceutical). NK cells were induced from CD3 negative CBMCs for 5 days, and $CD56^+$ NK and $CD3^+$ T cells in culture cells were assessed by flow cytometry using the fluorophore-conjugated antibodies against CD56 and CD3. Activated NK cells were transduced with CD19 CAR-pseudotyped LVs on day 5. Two days later, a second round of feeder cell stimulation was performed to amplify CAR-NK cells. On day 14, fresh CAR-NK cells were harvested and sent to clinical faculty for infusion.
(6) Release criteria for CAR-NK cell products include the following:

- Cell viability: ≥ 90%;
- $CD56^+$ NK cells ≥ 80%;
- $CD3^+$ cells: ≤ 1.0%;
- Feeder cells: ≤ 0.5%
- CAR^+ NK cells: ≥ 10%.
- Endotoxin: ≤ 0.5 EU/mL;
- Mycoplasma: negative;
- Bacterial culture: negative;

- Fungal culture: negative.

3.6 Study procedures and related examinations

3.6.1 Screening

Subject Screening: The screening period begins when the subject signs the IRB/IEC-approved ICF and continues until subject enrollment is confirmed. Informed consent must be obtained before completing any study-specific procedures for non-standard care.

After receiving written informed consent, subjects will be screened to confirm study eligibility and participation. Only participants who met the inclusion criteria could be enrolled in the study. The subject who does not meet the inclusion criteria before enrollment will be designated as a screening failure. The reason for the screening failure should be recorded.

Complete the following assessments/examinations during the screening period:

- Medical history and disease evaluation
- The physical examination includes height and weight
    - Subjects presenting with symptoms of central nervous system malignancy, such as severe headaches, stiff neck, or any local findings of nervous system on physical examination, will undergo a lumbar puncture for cerebrospinal fluid examination.
- Vital signs include blood pressure, heart rate, oxygen saturation, and body temperature.
- ECOG physical status
- Neurological assessment using MMSE
- Electrocardiogram
- Echocardiography for LVEF and pericardial effusion assessment
    - An echocardiogram may be performed within 28 days before the prior chemotherapy and signed informed consent for confirmation of eligibility.
- Diagnostic imaging
    - Brain MRI
    - Baseline PET CT
    1) PET-CT may be performed between patient’s last chemotherapy and signing informed consent to confirm enrollment eligibility.
    2) If a PET CT is performed > 28 days before initiation of conditioning chemotherapy or if the subject receives any anti-cancer treatment between screening and conditioning chemotherapy, the scan must be repeated to determine a new baseline. PET CT should be performed as close to enrollment as possible.
- Laboratory examination

- Chemical examination
- Blood counts and categorical counts
- Virological testing (HIV, HBV, and HCV, etc)
- Beta-HCG pregnancy test (serum or urine) for all fertile women

➢ Reports of serious adverse events (see section 6).

➢ Records for concomitant medication and previous cancer treatment history.

➢ Collection of paraffin specimen, fresh tumor samples, and cerebrospinal fluid samples (for subjects who signed the informed consent).

Screening of cord blood from donor: Cord blood is provided by Zhejiang Province Blood Center Cord Blood Bank and meets the following criteria:

1) Pathogen test: HIV, HBV (surface antigen and core antibody), HCV, syphilis, CMV, EBV, HTLV, and HPV B19 test results should be negative;
2) nose/throat swab nucleic acid test by RT-PCR are negative for COVID-19;
3) Negative for fungi, bacteria, mycoplasma and endotoxins

3.6.2 Generation and quality control of CAR-NK cells

3.6.2.1 CAR-NK cell generation location

The preparation of CAR-NK cells used in this study was completed in GMP laboratory of the Biologic Therapy Center, which is the Stem Cell Research Base approved by National Health Commission of the People's Republic of China.

3.6.2.2 Standard Procedure for CAR-NK cell culture

The umbilical cord blood for this study comes from the Zhejiang Province Blood Center Cord Blood Bank. 50~100 mL umbilical cord blood was obtained, and umbilical cord blood mononuclear cells (CBMCs) were isolated by Ficoll density gradient centrifugation. $CD3^+$ T cells were depleted from CBMCs with the $CD3^+$ sorting kit. The CD3 negative CBMCs were stimulated with lethally irradiated feeder cells expressing membrane-bound IL-21 and 41-BB ligand at a 2:1 ratio. Cells were maintained in OpTmizer ™CTS™ T-Cell Expansion SFM basic Medium (Gibco) with 2 mmol/L GlutaMAX (Thermo Fisher), 10% Human AB serum (Sigma), and 1000 IU/mL human IL-2 (Quangang, Shandong). On day 5 of culture, the phenotype of culture cells and feeder cells were detected by flow cytometry using a panel of antibodies, and then NK cells were transduced with a BaEV-LV encoding CD19-BBz CAR. Two days later, a second round of feeder cell stimulation was performed for CAR-NK cell expansion. On day 14, CAR-NK cells were harvested and tested for:

➢ Number of cells;

➢ Cell morphology under the microscope;

➢ CAR+ NK cell ratio: > 10%;

➢ CD3+ cell number: < 1.0%;

➢ NK cells (CD56+): > 80%;

- Mycoplasma, fungi, bacteria, endotoxin: negative;
- Cell viability: > 90%;
- CD137L Feeder cells < 0.5%.

CAR-NK cell infusion: intravenous infusion of 100-300 ml CAR-NK cell suspension (CAR-NK cells + normal saline + 2% human serum albumin).

Detailed Procedure:

(1) Isolation of NK cells

50~100mL umbilical cord blood was obtained from Zhejiang Province Blood Center and blood bag was sterilized with alcohol before being transferred to the biosafety cabinet. Used the tweezers to open the blood bag, extracted umbilical cord blood from the bag and transferred the blood into a 50 mL centrifuge tube. The umbilical cord blood was diluted with normal saline by 1:2. After mixing, The diluted cord blood was slowly added into the Ficoll (v/v 1:1) and centrifuged for 20 min (800 g, 4° C; speed regulation: acceleration=1, deceleration=0).

After centrifugation, there is a buffy coat formation. Discard the upper plasma layer on the buffy coat, and then absorb the buffy coat gently using a 3 mL pipet. The buffy coat cells were washed twice using normal saline (centrifugation conditions were 400 g, 5 min, and 300 g, 10 min, respectively). CBMCs were obtained, and $CD3^+$ T cells were depleted from CBMCs using the $CD3^+$ sorting kit (Miltenyi).

Cell numbers were determined, and the cell suspension was centrifuged at 300×g for 10 minutes. Pipette off supernatant completely and resuspend the cell pellet in 80 μL of buffer per 10^7 cells. 20 μL of CD3 MicroBeads per 10^7 total cells were added, mixed, and incubated for 15 minutes at 4−8 °C. Wash cells by adding 1-2 mL of buffer per 10^7 cells and centrifuge at 300 g for 10 minutes. Pipette off supernatant completely, resuspend up to 10^8 cells in 500 μL of buffer, and then proceed to magnetic separation.

For magnetic separation, place the column in the magnetic field of a suitable MACS Separator. Prepare the column by rinsing it with 3 mL of buffer. Apply cell suspension onto the column. Collect unlabeled cells that pass through and wash the column with the appropriate buffer. Perform washing steps by adding buffer three times once the column reservoir is empty. Collect total effluent, and this is the CD3 negative CBMCs. Count the cell number and detect the expression of CD45/CD3/CD56 by flow cytometry. Cryopreserved the cells in liquid nitrogen for subsequent CAR-NK cell preparation.

(2) CAR-NK cell activation, transduction, and proliferation

(a) Thaw frozen CBMCs after finishing the subject screen assessment.

(b) CAR-NK cell activation

After 6-24 hours of recovery of thawing cells, count the number of CBMCs and prepare them for activation.

(c) Prepare Feeder cells

Day 0: Add the Feeder cells into the CBMCs at the ratio of 1:2 and change the fresh medium every day.

Day 5: Determine the purity of NK cells by anti-CD3/CD56/CD137L antibody to avoid T cell and Feeder cell contamination.

(c) CAR lentiviral vector transduction

Day 5: Centrifuge (400 g, 5 minutes) to collect the NK cells and transduced CAR lentivirus vector as follows: Adjust the cell density as 5×10^6/mL, add the 5 MOI (multiplicity of infection) of the lentiviral vector and 8 μg/mL protamine into the NK cells. Mix gently, transfer the cell-virus mixture into the 96-well plate, and centrifuged at 32°C at 1200 g for 90 minutes. After an additional 4-hour culture, the residual virus was removed, replaced the fresh culture medium, and continued to culture the CAR-NK cell with a cell density of 0.5×10^6/ mL.

(d) Expansion and detection of CAR-NK cells

Day 7: Observe cell status, including colony size, cell density, cell morphology, cell debris, medium color, etc. For detection of transduction efficiency, 0.1-0.2×10^6 cells were stained with anti-CD3/CD56 and anti-FMC63 antibodies and then incubated on ice for 20 min. After washing with phosphate buffer containing 2% fetal bovine serum 1-2 times, percentage of CAR+ cells was detected by flow cytometry.

(e) Expansion of CAR-NK cells

CAR-NK cells were added with Feeder cells at 1:2 for secondary stimulation.

On days 9-12, CAR-NK cells were expansion. Pay close attention to the cell density and growth state and timely replenishment to maintain the cell density at 0.5×10^6/mL.

Cell purity (e.g., CAR%, CD3/CD56 expression, CD137L) and microorganisms (e.g., fungi, bacteria, mycoplasma, endotoxin, etc.) were tested 24 hours before cell harvest.

(3) CAR-NK cell harvest

On days 13-14, shake the cell culture bottle until no apparent cell clusters are visible. The cells were transferred into 50 mL and collected at 400 g for 5 min. Wash them twice and resuspend the cells in an infusion buffer containing 2% HSA. Determine the cell numbers and transfer them into a transfusion bag with 4-6×10^6 cells /mL density. Sealed up the transfusion bag, labeled with the patient's name, cell type and number, and date, and transferred to the clinical department within 30 minutes. At the same time, reserved a tube of the infusion samples for further safety testing.

3.6.3 CAR NK cells Infusion

Pre-infusion: Before CAR NK cell infusion, the nurse should check the information about the patient and CAR-NK product, and strictly check whether the cell suspension has flocculence and turbidity, gently reverse the product bag up and down three times, thoroughly mix, then the CAR-NK product was given to the patients by intravenous infusion according to the operating procedures of transfusion. The infusion comply with aseptic operation. To prevent transfusion reaction, 25mg of phenergan is allowed to inject intramuscularly at 30 minutes before CAR-NK cells infusion.

Infusion period: During the infusion of CAR NK cells, it is necessary to pay attention to cell blockage or adherence to the tube wall. The injection hose can be lightly flip during infusion to keep the injection smoothly. The infusion of CAR-NK cells should be avoid to mix with other liquids. It is necessary to inject the CAR NK cells separately and observe leakage of percutaneous puncture. The immune cells, biological products, should be infused using disposable sterile transfusion apparatus. Adjust 10-15 drops /min of slow infusion for 15 minutes during begin of infusion. If the patient had no infusion reaction, infusion speed was then adjusted to 40-60 drops/min. During the transfusion, if there is cell mass in the filter, it can be rinsed with normal saline and then infusion continues. It should observe the symptoms such as chills, fever, palpitation, chest tightness, shortness of breath, loss of appetite, muscle and joint pain, rash, and mental disorders, which are needed to be treated promptly.

CAR-NK cell production process:

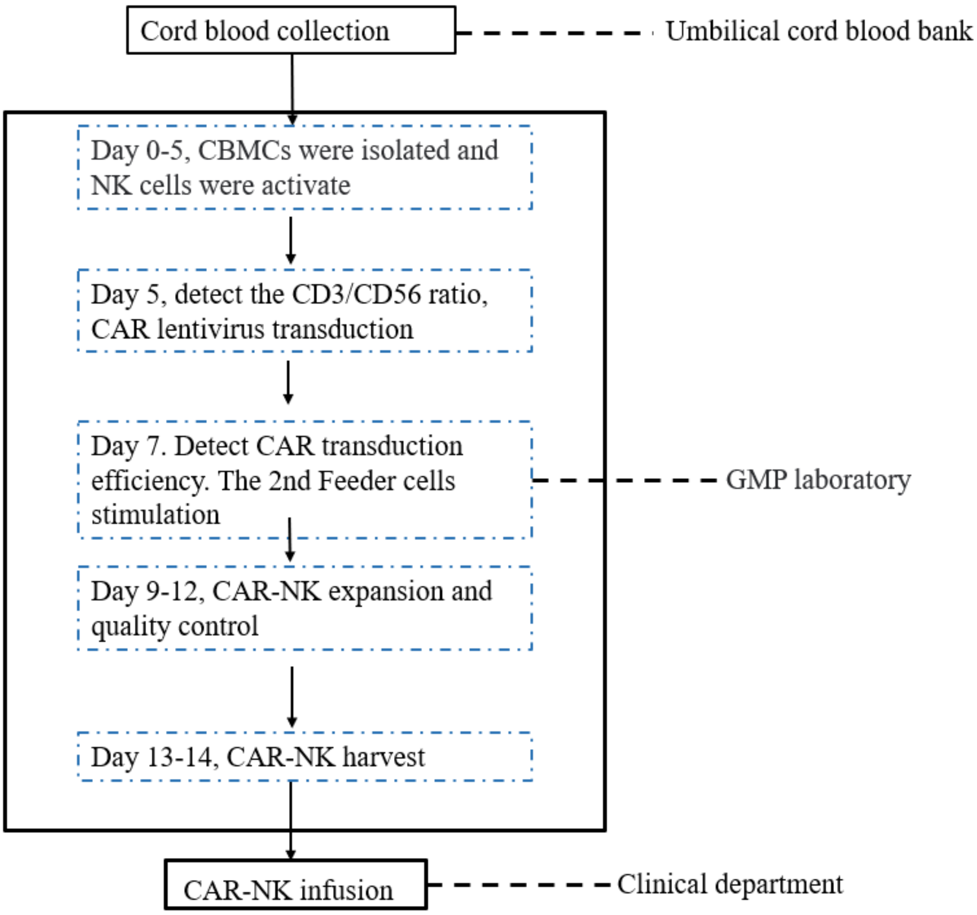

3.6.4 Shipment and handover of cord blood and CAR-NK cell product

3.6.4.1 Shipment of Cord Blood

(1) The cord blood was provided by Cord Blood Bank. The employee of Cord Blood Bank should inform the laboratory to get the cord blood and temporarily store the cord blood in the refrigerator (4°C~10°C).

(2) The handover of cord blood between transporter and employee of Cord Blood Bank should abide by the role: at the appointed time, signing the document of "Record of handover of cord blood". It should be provided by Zhejiang Province Blood Center Cord Blood Bank, which included "Report of Viruses Tests for Cord Blood" and "Written Informed Consent for Cord Blood Collection".

(3) During handover, the transporter should check whether the identification information is consistent with the record of cord blood. Check the blood bag for leakage. Check whether the sample is stored at the appropriate temperature. If it has any mistake, the handover should be stopped, and the reasons for stopping handover should be recorded.

(4) The transporter will store the cord blood in a refrigerator (4~10°C). The sample was transferred to the laboratory within 12 hours after collection.

3.6.4.2 Handover between transporter and staff of the laboratory

(1) The staff check whether the information of cord blood is consistent with the transfer list at the time of handover; Check the blood bag for leaks; Check whether the transport temperature is 4~10℃;
(2) After confirmation, sign the “Record of handover of cord blood between transporter and Lab”.
(3) If there is any mistake, this handover should be stopped, and the reasons for stopping handover should be recorded.

3.6.4.3 Release of CAR-NK product and monitoring after usage

(1) CAR-NK cell product should meet the Release criteria.
(2) Samples should be obtained from released product, which were frozen at -80℃ for future examination if it is necessary.
(3) Fill in the "Cell final Product Packaging and return monitoring Form" at release time. Notify the transporter to put the CAR-NK product into a refrigerator and deliver them to the clinical department within 4 hours, keeping the transfer temperature within 4~10℃.

3.6.5 Quality standard and quality control report

(1) Quality control standards and reference criteria

The quality control standard of CAR-NK products refers to the relevant procedures for biological products of the “Chinese Pharmacopoeia” and the “Technical Guidelines for Human Cell Therapy Research and Preparation Quality Control” (2003 version) issued by the Drug Evaluation Center as well as the “Principle of Study and Quality Control of Human Cell Therapy” (2017 version) issued by the Food and Drug Administration of China. The following table is specially formulated for the quality control of CAR-NK cell products, including endotoxin detection, fungal bacteria detection, mycoplasma detection, cell number, cell survival, CAR^+ cell proportion, and cell phenotype (see Table 1).

Table 1. Quality criteria of CAR-NK cell products

Number	Test index	Release
1	Cell count	According to clinical protocol

2	Cell viability	≥90%
3	$CD3^+$ T cells (%)	< 1.0%
4	CAR^+NK (%)	≥ 10%
5	Mycoplasma	Negative
6	Bacterial	Aseptic
7	Fungal	Aseptic
8	Bacterial endotoxin	＜0.08EU/mL
9	Appearance	No flocculent precipitation, no foreign matter
10	Residual Feeder cells	< 0.5%

Fungal culture, bacterial culture, mycoplasma, and endotoxin tests were carried out by the laboratory of the Second Affiliated Hospital, College of Medicine, Zhejiang University, and the results should be provided.

3.6.6 Conditioning Chemotherapy

Before conditioning chemotherapy, subjects must have no clinically significance infection, no clinically significance cardiac dysfunction, serum creatinine < 2 × upper limit of normal, and no > grade 1 acute neurotoxicity (except peripheral sensory neuropathy). If any of these events occurs before conditioning chemotherapy, conditioning chemotherapy must be postponed until the event has resolved.

From day -6 to day-1, the following examination and procedures will be done:

- Vital signs include blood pressure, heart rate, oxygen saturation, and body temperature
- Laboratory tests (blood sample drawn before chemotherapy)
- Chemical examination
- Complete blood counts and categorical counts
- Drugs check: Fludarabine, cyclophosphamide
- Adverse/serious adverse event reports
- Combined medication records

3.6.7 CAR-NK019 Treatment

Subjects will be hospitalized to receive CAR-NK019 cell therapy, followed by an observation period. Before CAR-NK019 infusion, subjects must have no clinically significance infection,

cardiac dysfunction, serum creatinine < the upper limit of normal 2×, and no acute grade 1 neurotoxicity (except peripheral sensory neuropathy). In addition, subjects must not receive systemic antimicrobials for active infections within 48 hours prior to CAR-NK019 administration (prophylactic use of antimicrobials is permitted). If these criteria are not met before receiving CAR-NK019 therapy, the infusion must be postponed until the event resolves. If CAR-NK019 infusion is delayed > 2 days, conditioning chemotherapy must be repeated.

Subjects will be hospitalized until day 7 after CAR-NK019 treatment. Subjects should not discharge until CAR-NK019-related nonhematologic toxicity returns to ≤ grade 1 or baseline. If the investigator deems it appropriate, the subject may discharge even if > Grade 1 is present in cases of non-critical and clinically stable or slowly improving toxicity (e.g., renal insufficiency). Subjects may remain hospitalized if they develop persistent CAR-NK019-associated fever, hypotension, hypoxia, or persistent CNS toxicity > grade 1, or if the investigator deems it necessary.

Given the potential for CRS or neurotoxicity after discharge, subjects and their family members or caregivers should be trained to know about potential symptoms, such as fever, dyspnea, confusion, aphasia, stunted growth, lethargy, encephalopathy, ataxia, or tremors. If a subject develops these symptoms, the subject should contact the principal investigator immediately or seek medical attention immediately.

During this period, the following tests and procedures must be completed:

- Mini-Mental State Examination (MMSE)
  - MMSE will be used to evaluate neurotoxicity before the start of CAR-NK019 treatment on day 0, on day 1, and every other day during hospitalization.
- Vital signs, including blood pressure, heart rate, oxygen saturation, and temperature, will be observed every 4-6 hours during the hospital stay.
- Laboratory tests (as described in the evaluation plan prior to CAR-NK019 infusion)
  - Chemical examination
  - Complete blood count and cell subtype count, lymphocyte subpopulation test, cytokine level
  - CD19 CAR-NK detection
- CAR-NK019 infusion
- Where applicable, subjects who have ≥2 new neurological symptoms after completion of CAR-NK019 infusion or who have signed the informed consent should have a lumbar puncture to examine cerebrospinal fluid.
- Tumor sample collection from subjects who signed the informed consent (any time between days 7 and 14)
- Adverse event/Serious adverse Event reports.
- Combined medication records

Monitoring C-reactive protein, ferritin, and LDH levels (only when LDH levels are elevated at baseline) may help diagnose and define CRS/neurotoxic-related clinical processes. Therefore, daily

monitoring of C-reactive protein, ferritin, and LDH (if elevated at baseline) is recommended, starting on day 0 and continuing during hospitalization. In addition, lactate should be monitored according to clinical indications.

3.6.8 Post-treatment evaluation period

After CAR-NK019 infusion and discharge, all subjects should be followed up during the post-treatment evaluation phase. The subjects will return to the clinic at the following intervals (day 0: CAR-NK019 infusion).

- ✧ Day 28
- ✧ 2nd month (±1 week)
- ✧ 3rd month (±1 week)

The subject will allow the investigator contact to continue to access medical records to obtain information about the subject's health status and initial treatment response. The following procedures will be completed for the subject in accordance with the assessment plan:

- ➢ MMSE
- ➢ PET-CT for disease assessment: If PET-CT does not have a high enough resolution, the scan must be repeated.
- ➢ Physical examination
- ➢ Vital signs include blood pressure, heart rate, oxygen saturation, and body temperature
- ➢ Laboratory examination
    - ◆ Chemical examination
    - ◆ Complete blood count and subtype count
    - ◆ Beta-HCG pregnancy test (serum or urine) for all fertile women
    - ◆ Cytokine levels
    - ◆ Lymphocyte subsets detection
    - ◆ CD19 CAR-NK cells
    - ◆ Adverse event/Serious adverse Event reports.
    - ◆ Combined medication records

If the subject is subsequently re-hospitalized with any CAR-NK019-related adverse events, the following procedures will be performed as outlined in the evaluation plan:

- ➢ Laboratory examination on the day of admission, then once a week, and on the day of discharge.
- ➢ Cytokine levels on the day of admission, once a week, and on the day of discharge.

At any time during the post-treatment evaluation period, if the subject does not respond to treatment or develops disease progression after response, the investigator decides the salvage treatment according to the willing of subject, and survival and disease outcomes of the subject will be follow-up.

3.6.9 Long-term follow-up period

After 3 months of post-treatment, all enrolled subjects will enter a long-term follow-up period for survival and disease status.

- Every 3 months (±2 weeks) until the 12th month
- Every 6 months between the 12th and 24th months (±1 month)

Participants and receiving CAR-NK019 therapy will be followed up at the time points outlined in the evaluation plan:

- Physical examination
- Disease evaluation up to 24 months or until disease progression.
- Living status
- Laboratory examination
  - Complete blood count and subtype count
  - Lymphocyte subsets detection
  - CD19 CAR-NK cells
- Reports of targeted adverse events/serious adverse events (up to 24 months or until disease progression)
  - including nerve, blood toxicity, infection, autoimmune diseases, and secondary tumors until disease progression.
- Records of concomitant medication, including gamma globulin, immunosuppressive drugs, anti-infective drugs, and vaccinations

Subjects may also be contacted by telephone to confirm survival status and report on concomitant medication. If a subject requires laboratory sample collection, it can be done in a clinic or outside facility to reduce the burden on the subject.

If the subject fails to return to the clinic for a planned visit, the investigator will make the attempts to contact the subject by phone and mail. The center must record three attempts to contact the subject. If the subject does not respond within 1 month of the third contact, the subject should be considered loss to follow up, and no additional contact is required.

3.7 Endpoint Evaluation

3.7.1 Safety Endpoint Evaluation

Incidence of "probable" and "certain" adverse events and abnormal laboratory test results associated with CAR-NK019 treatment, including dose-limiting toxicity (DLT), occurring at any time from the start of study treatment to the 28th day.

3.7.2 Efficacy Endpoint Evaluation

Objective response rate (ORR): the proportion of all treated subjects who achieve CR or PR.

Proportion of complete response (CR): CR cases/total cases.

Progression-free survival (PFS): The time between the first day of infusion and the onset of disease

progression or death.

Duration of response (DOR): The time from the first CR or PR to the onset of disease progression.

CT (or MRI) and ultrasonography: After one month, imaging examination will be used to disease status, until disease progression or intolerable toxicity or withdrawal of informed consent. PET-CT will be used if disease progression occurs.

Bone marrow examination: If no bone marrow invasion during the screening period, and subsequent bone marrow examination is not needed. When recurrence or progression of disease is suspected, investigator decides whether perform a bone marrow examination. For subjects with bone marrow involvement at baseline, if efficacy evaluation achieved CR, a bone marrow aspiration/biopsy is required to confirm efficacy (completed within 2 weeks of PET-CT evaluation).

3.8 Concomitant medication and treatment

Concomitant medication should be decided by the investigator (e.g., combination drugs used to treat disease-related symptoms and treatment-related adverse events), which is allowed.

Drugs and therapies to treat complications or relieve symptoms of complications, such as antihypertensive drugs, antibacterics, antihistamines, etc., are permitted, but adjustments in dose and administration mode should be minimized during the study.

3.9 Research procedure

FLOW CHART

**Research stage**	**Screen**	**CARNK preparation**	**Baseline**	**FC Chemotherapy**	**CAR-NK019 infusion**	**Safety and efficacy follow-up period**									**Survival follow-up period**				
Follow-up	Follow-up 1	Follow-up 2	Follow-up 3	Follow-up 4	Follow-up 5	Follow-up 6	Follow-up 7	Follow-up 8	Follow-up 9	Follow-up 10	Follow-up 11	Follow-up 12	Follow-up13	Follow-up14	Follow-up 15	Follow-up16	Follow-up17	Follow-up18	Follow-up18
Weeks/ Years	-3	-2	-2	-1	1	1	1	2	2	2	3	4	8	12	25	38	1Y	2Y	3Y
Days	-21	-14	-7~ -5	-6~ -3	0	1	4	7	10	14	21	28	60±3	90±3	180±7	270±7	360±7	720±7	1080±7
Sign informed consent	×																		
Inclusion/exclusion criteria	×																		
**Tumor and disease assessment**																			
B-NHL treatment history	×																		
Tumor evaluation	×																		
Immunophenotyping of BM[1]	×																		
CT examination of head, chest, abdomen and pelvis [2]	×																		
PET-CT examination [3]			×									×		×	×	×	×	×	×
**Research steps and examination**																			
Demographic characteristic	×																		
Medical history	×																		

Physical examination	×																		
ECOG Score	×																		
Electrocardiograph	×						×		×	×	×	×	×	×					
Lung function [4]	×																		
Electrocardiograph monitoring [5]					×														
Humoral immunoassay [6]	×	×				×		×	×	×	×	×	×	×	×				
Vital sign	×		×	×	×	×	×	×	×	×	×	×	×	×					
Adverse Event	×		×	×	×	×	×	×	×	×	×	×	×	×	×	×	×	×	×
Combination therapy	×		×	×	×	×	×	×	×	×	×	×	×	×	×	×	×	×	×
**Hospital routine examination**																			
Blood routine examination	×		×	×	×	×	×	×	×	×	×	×	×	×	×	×	×	×	×
Blood biochemistry	×		×	×	×	×	×	×	×	×	×	×	×	×	×	×	×	×	×
Urine routines			×				×		×	×									
Stool routine			×				×		×	×									
Coagulation function	×		×		×	×	×	×	×	×									
Serum cytokine [7]			×		×	×	×	×	×	×									
Peripheral blood lymphocyte subsets			×		×	×	×	×	×	×	×	×	×	×					
Ferroprotein			×		×	×	×	×	×	×									
Infectious disease and virus detection	×																		
Pregnancy tests	×																		
CAR gene copy number in peripheral blood					×	×	×	×	×	×	×	×	×	×	×	×	×	×	×
CAR-NK ratio in peripheral blood					×	×	×	×	×	×	×	×	×	×	×	×	×	×	×
Peripheral blood CAR-							×	×	×	×	×	×	×	×	×	×	×	×	×

NK antibody detection																			
Reproducible lentivirus RCL monitoring														×		×		×	
**CAR-NK infusion**																			
Conditioning chemotherapy				×															
CAR-NK infusion					×														

Footnote

1. For subjects without bone marrow invasion, bone marrow cell immunophenotyping was not performed in the follow-up period;
2. CT examination results within 4 weeks before signing the informed consent form can be used as screening data;
3. At visit 2, PET-CT was detected before conditioning chemotherapy; whether to perform PET-CT examination in the long-term follow-up period is determined according to clinical conditions;
4. To evaluate lung inflammation 2~3 days before the start of chemotherapy;
5. From 30 minutes before the CAR-NK infusion to 2 hours after the transfusion, routine ECG monitoring (30 minutes before the infusion to 1 hour after the transfusion) was recorded every 30 minutes; after 1 hour after infusion, once every 1 hour). for example, the vital signs are not stable after CAR-NK infusion, and the monitoring time is extended until the vital signs are stable;
6. If necessary, humoral immunoassay can detect the following items: IGG/A/M/E, κ-light chain, λ-light chain, β2 microglobulin;
7. Serum cytokine detection includes: CRP, IL-2/4/6/10, TNF-α, IFN-γ, IL-17A, MCP-1, ANG-1, and ANG-2, among which CRP, IL-2/4/6/10, TNF-α, and IFN-γ will be detected in the research center, and samples of other cytokines will be uniformly sent to the central laboratory for detection.

4. Sample Size Considerations

4.1 Part I sample size calculation

The primary endpoints of dose-escalation study (Part I) were MTD and DLT, approximately 9-18 patients were enrolled according to the "3+3" dose escalation principle. Therefore, 9 to 18 patients were needed in this phase.

4.2 Part II sample size calculation

(1) Hypothesis
The planned dose used in the expansion study (Part II) is based on the review of the preliminary safety and efficacy observed in dose escalation phase (Part I) and further differentiates between a treatment with a true response rate of 25% or less and a treatment with a true response rate of 55%. The hypothesis is that the objective response rate to CD19 CAR-NK cells in the R/R LBCL is significantly greater than 30%.
(2) Sample size consideration
Phase II uses a single-arm design to test for improved response rate in the R/R LBCL. For the test of efficacy, this study has ≥ 90% power to distinguish between an active therapy with a 55% ORR from treatment with an ORR of 25% or less with a 1-sided alpha of 0.025, considering a 15% dropout rate, a total of 30 samples is required.
(3) Statistical Assumptions
Treatment outcomes for R/R LBCL are provided in Table 2 below. To evaluate the validity of the assumption on the underlying response rate, retrospective studies (historical data and database reviews) of the response rate in the target population will be conducted. The patient response to conventional treatments (including radiotherapy and immunochemotherapy) varies between 14% and 23%. Meanwhile, the response to CAR-T or CAR-NK cell therapy is 52% to 73%. Based on these data, this study assumes that the underlying objective response rate (in the conventional treatments) among the relapsed or refractory population is estimated to be 25% and that an improvement in the response rate to 55% provides clinically meaningful benefit.

Table 2. Historical Responses in R/R LBCL

Setting	Outcome to Subsequent Therapy
Conventional	
Ardeshna et al 2005 (n=28)	ORR 18%
Seshadri et al 2008 (n=73)	ORR 14%
Telio et al 2012 (n=111)	ORR 23%
Phillip et al 1995 (n=28)	ORR 21%
Crump et al 2014 (n= 189)	ORR 26%
CAR-T	
Abramson et al 2020 (n=344)	ORR 73%
Schuster et al 2018 (n=93)	ORR 52%
Xuan Zhou et al 2020 (n=21)	ORR 67%
Ying et al 2019 (n=29)	ORR 58.6%
Ying Z et al 2021(n=58)	ORR 60.3%
CAR-NK	
Enli Liu et al 2020 (n=11)	ORR 73%

5. Statistical Analysis

Statistical analysis was performed using SAS® version 9.2. Unless otherwise specified, the measurement data are generally described by the number of cases, mean, standard deviation, median, minimum and maximum values. Enumeration data are generally described by case numbers and percentage. The Kaplan-Meier method was used to estimate the follow up time for survival, PFS, and DOR. Its overall 95% confidence interval for time-event data.

Baseline evaluation

Demographic variables and baseline characteristics (e.g., sex, age, weight, disease category, drug concomitant, past treatment history, vital signs, etc.) were summarized by dose group.

Safety evaluation

Safety statistical analysis included descriptive statistics for adverse events, vital signs, laboratory test values, dose-limiting toxicity (DLT), and electrocardiogram (ECG).

Effectiveness evaluation

Statistical efficacy measures evaluated by the investigators included OS, PFS, CR, PR, SD and PD. ORR defined as CR + PR assessed using Lugano 2014 criteria. Kaplan-Meier curves were used to describe OS based on the full analysis set, and the median OS, the first and third quartiles were summarized and described. The number and percentage of other non-time event efficacy indicators were summarized and described.

5.1 Analysis set

- Modified Intention-to-Treat Analysis Set (mITT): The mITT analysis set included all subjects enrolled and treated with a CD19 CAR-NK target dose. This analysis set will be used for the analysis of safety (exception of MTD) and efficacy;
- The MTD Analysis Set: All subjects in Part I study who received CAR-NK cell infusion and either completed 28-days of follow-up, or who had a DLT;
- Full Analysis Set (FAS) : All participants who have used study drugs will be included in this analysis set. FAS sets are primarily used for baseline and validity analysis.
- Safety Analysis Set (SS) : Included all subjects who had received at least one study drug treatment;

5.2 Data Analysis

This study included two phases. Part I is dose-escalation phase, which plan to enroll 9 to 18 cases. End of this phase study, primary safety and efficacy will be analyzed and reported. Part II is expansion phase study that plan to enroll 30 cases. End of Part II study, the data from all subjects enrolled in two phase study will be analyzed for safety and efficacy.

6. Data management and confidentiality

6.1 Data collection and management

The principle investigator will reserve a list of investigator entrusted with research responsibilities. The individuals authorized to perform such duties shall be outlined and included in the power of attorney.

Original documents are the documents of research data and records which are collected and verified. Examples of such original documents may include, but are not limited to, hospital and patient records, laboratory, pharmacy, radiological records, subject dates, microfilms, communications, and death registrations. If the original data collection source does not exist, the data in the case report form will be considered to be the original data. However, it is not recommended to use the CRF as a raw record in general operations.

The investigator and study staff are responsible for reserving a comprehensive and centralized archiving system of all subject records that is easily accessible for monitoring and/or auditing by primary sponsor contact personnel, regulatory authorities and IRB/IEC. The filing system will include at least the following information:

Subject consent includes ICF and Subject identification list

Protocol and revised protocol, Investigator's manual, copies of pre-study records, and all information communicating with IRB/IEC and Sponsor.

Acceptance certificate, experimental treatment procedure records, and communication about experimental products.

The information recorded in the CRF must be kept at the research centre and is available on request. No research document may be disposed of without prior written agreement between the sponsor and the investigator. If the stored information is no longer available for archiving the original document or must be transferred to another location, the researcher shall inform the primary sponsor contact before shipping the document.

6.2 Data confidentiality

The results of research conducted through this project may be published in medical journals, but we will keep patient information confidential as required by law, and patient personal information will not be disclosed unless required by relevant law. When necessary, the government management department and the hospital ethics committee and its relevant personnel may consult the patient's data according to regulations.

7. Informed consent

Before subjects participate in this study, the investigator is responsible for explaining the study design, expected benefits, and potential risks to the subjects and it is necessary to obtain written informed consent from the subjects. Subjects should sign the latest IRB/IEC approved ICF before any research-specific activities or procedures.

Informed consent must be in full compliance with ICH, GCP, and local regulatory and legal requirements and must be approved by the IRB/IEC for use in this study and for any changes made during the study.

The process of informed consent, agreement or refusal to participate of subjects should be recorded in the subject's medical records. If the subject agrees to participate in the study, the subject and the person evaluating the informed consent should sign and date the informed consent. The original signed ICF will be retained in accordance with institutional policy and IRB/IEC requirements, and a signed copy of the informed consent will be provided to the subject.

8. Observation of adverse events

8.1 Definition of Adverse Events

8.1.1 Definitions

Adverse Events: All adverse events that occur in this study, including AEs might be not related to the treatment.

Serious Adverse Events: AEs that require hospitalization, prolong hospitalization, disability, affect working ability, life threatening or death, and lead to malformations occurring during clinical trials.

8.1.2 Grades

Mild (Grade 1): the subject can tolerate, does not affect treatment, does not require special treatment, and has no impact on the subject's recovery.

Moderate (Grade 2): The subject is unbearable, requires treatment or rest, and directly impacts the subject's recovery.

Severe (Grade 3): The AE significantly affect the subject's ability to perform routine activity, requiring immediate emergency treatment.

Severe (Grade 4): Under immediate risk of death

Severe (Grade 5): Death

8.2 Risk-prevention and treatment

8.2.1 Cytokine Release Syndrome

Cytokine release syndrome (CRS) results from the activation of immune cells related to CAR-T cell expansion and marked elevations of serum inflammatory markers and cytokines. CRS is the most serious adverse event for CAR-T therapy. Clinical signs and symptoms associated with the CRS include cardiac, gastrointestinal, laboratory abnormalities (clotting, kidney, and liver), respiratory, skin, vascular (hypotension), and systemic (fever, chills, headache, discomfort, fatigue, joint pain, nausea, and vomiting) signs.

CAR-NK cell is another adoptive immune cell therapy. It may also have the risk of CRS. The grade and management of CRS in CAR-NK cell therapy refers to CAR-T cell therapy according to the American Society of Transplantation and Cellular Therapy Consensus grading system. The grading scale is outlined in Table 3, The clinical signs and symptoms of CRS are in Table 4, and the treatment guidelines outlined in Table 5.

Table 3. ASTCT CRS Consensus Grading

CRS Parameter	Grade1	Grade2	Grade3	Grade4
Fever*	Temperature ≥38 °C	Temperature ≥38 °C	Temperature ≥38 °C	Temperature ≥38 °C
		With		
Hypotension	None	Not requiring vasopressors	Requiring a vasopressor with or without vasopressin	Requiring multiple vasopressors (excluding vasopressin)
		And/Or#		
Hypoxia	None	Requiring low-flow nasal cannula$ or blow-by	Requiring high-flow nasal cannula, facemask, nonrebreather mask, or Venturi mask	Requiring positive pressure (eg.CPAP, BiPAP, intubation and mechanical ventilation)

Organ toxicities associated with CRS may be graded according to CTCAE v5.0 but they do not influence CRS grading.

* Fever is defined as temperature ≥38 °C not attributable to any other cause. In patients who have CRS then receive antipyretic or anticytokine therapy such as tocilizumab or steroids, fever is no longer required to grade subsequent CRS severity. In this case, CRS grading is driven by hypotension and/or hypoxia.

CRS grade is determined by the more severe event: hypotension or hypoxia not attributable to any other cause. For example, a patient with temperature of 39.5 °C, hypotension requiring 1 vasopressor, and hypoxia requiring low-flow nasal cannula is classified as grade 3 CRS.

$ Low-flow nasal cannula is defined as oxygen delivered at ≤6 L/minute. Low flow also includes blow-by oxygen delivery, sometimes used in pediatrics. High-flow nasal cannula is defined as oxygen delivered at >6 L/minute.

Table 4. Clinical Signs and Symptoms Associated with CRS

Organ System	Symptoms
Constitutional	Fever+/-rigors, malaise fatigue, anorexia, myalgias, arthralgias, nausea, vomiting, headache
Skin	Rash
Gastrointestinal	Nausea, vomiting, diarrhea
Respiratory	Tachypnea, hypoxemia
Cardiovascular	Tachycardia, widened pulase pressure, hypotension, increase cardiac output (early), potentially diminished cardiac output (late)
Coagulation	Elevated D-Dimer, hypofibrinogenemia+/-bleeding
Renal	Azotemia
Hepatic	Transaminitis, hyperbilirubinemia
Neurologic	Headache, mental status changes, confusion, delirium, word finding difficulty or frank aphasia, hallucinations, tremor, dysmetria, altered gait, seizures

Table 5. Treatment guidelines for CRS

Grading assessment of CRS	Widespread comorbidities or older age?	Treatment
Grade 1<br>Fever (defined as ≥ 38.3 °C)<br>Systemic symptoms	N/A	Vigilant supportive care<br>Assess infection<br>If fever and neutropenia develop, treat them, monitor fluid balance, and use antipyretics and analgesics<br>as needed
Grade 2<br>Hypotension: Responds to fluids or a low dose of vasopressin<br>Hypoxia: Response < 40% O^2<br>Organotoxicity: Grade 2	No	As described in Grade 1<br>Closely monitor cardiac and other organ function
Grade 3<br>Hypotension: Responds to fluids or a low dose of vasopressin<br>Hypoxia: Response < 40% O^2<br>Organotoxicity: Grade 2	Yes	As described in Grade 2<br>Consider Tocilizumab (8 mg/kg) ± corticosteroid (e.g. Methylprednisolone 1 mg/kg BID) or dexamethasone 10 mg q6hrs

Grade 3<br>Low blood pressure: Multiple vasopressin or high doses of vasopressin [a] are required<br>Oxygen deficiency: ≥40% O2 is required<br>Organotoxicity: Grade 3 or 4 elevated aminotransferase	N/A	
Grade 4<br>Mechanical ventilation<br>Organotoxicity: Grade 4 does not include elevated transaminase	N/A	As described in Grade 2/3<br>Corticosteroids (ex: methylprednisolone 1g/d × 3 followed by rapid decline consisting of 250 mg BID × 2 days, 125 mg, BID × 2 days, then 60 mg BID ×2 days)

Table 6. High Dose Vasopressors (all doses are required for ≥ 3 hs)

Pressor	Dose
Norepinephrine monotherapy	≥ 20 μg/min
Dopamine monotherapy	≥ 10 μg/kg/min
Phenylephrine monotherapy	≥ 200 μg/min
Epinephrine monotherapy	≥ 10 μg/min
If on vasopressin	Vasopressin + norepinephrine is equivalent to ≥ 10μg/min
If on combination vasopressors (not vasopressin）	Noradrenaline is equivalent to ≥ 20 μg/min*

*VASST Trail vasopressor equivalent equation: norepinephrine equivalent dose=[norepinephrine (μg/min)]+ [dopamine (μg/min)/2]+ [epinephrine (μg/min)]+ [phenylephrine (μg/min)/10

8.2.2 Hypotension and renal insufficiency

Hypotension and renal insufficiency should be treated according to medical judgment and medical guidelines. Prompt intravenous (IV) fluids may be required to manage blood vessel leakage during hypotension and CRS. Subjects should be closely monitored to prevent excess fluid, and in some cases continuous veno-venous hemodialysis may be required.

Invasive hemodynamic monitoring, such as the use of a pulmonary artery catheter, may help optimize fluid management during complications of severe capillary leakage, active IVF medication and fluid management are necessary for pulmonary edema. When blood pressure begins to drop below baseline, antihypertensive drugs should be suspended.

The mean of all values of blood pressure taken during the 24-hour period prior to CD19-CAR-NK infusion was defined as baseline blood pressure. The first line treatment for hypotension is intravenous saline infusion.

Subjects with systolic, diastolic, or mean arterial pressure ≤ 80% of baseline value or below the lower limit of normal should receive 1L saline infusion.

If hypotension does not respond adequately within 1 hour, a second infusion of the appropriate volume may be given at the judgement of the investigator.

If hypotension persists after 2 fluid injections, monitoring in the intensive care unit (ICU) with vasopressin support should be considered.

Note that these treatments may need to be modified based on medication guidelines and the individual subject's clinical characteristics, such as lung condition, heart function, and other factors.

8.2.3 Cardiac toxicity

Cardiac manifestations of CRS may include arrhythmias, decreased ejection fraction/heart failure, myocardial ischemia, and cardiac arrest. Tachycardia is common when CRS occurs, and drugs that slow sinus tachycardia should be avoided. Hypotension should be managed according to the above section.

Subjects with persistent hypotension who do not respond to fluid should be evaluated for decreased ejection fraction/heart failure by echocardiography. These toxicities should be managed in a timely manner based on medical judgment. Patients with grade 2 or higher cardiotoxicity should be monitored in the intensive care unit based on clinical indications. Tocilizumab and corticosteroids should be administered in accordance with section (8.2.1). Follow-up electrocardiograms and echocardiograms are recommended to monitor the progression from toxicity to potential remission.

8.2.4 Hemophilic lymphohistiocytosis

Hemophilic lymphohistiocytosis (HLH) is a clinical syndrome that includes extremely severe systemic inflammation, CRS, and multiple organ dysfunction (Jordan 2011, La Rosee 2015).

Symptoms include fever, hemocytopenia, liver dysfunction with hyperbilirubinemia, coagulopathy, tissue hemophagia, and significantly elevated ferritin, C-reactive protein, and soluble interleukin-2 receptor (sIL-2R) (Jordan 2011, La Rosee 2015, and Porter 2015). Abnormalities of the nervous system can be observed in about one-third of cases. Severe HLH may be triggered by infections, autoimmune disease and immunotherapy (Abe 2002, Ferreria 2006, Lackner 2008, La Rosee 2015). CRS and HLH may have similar clinical syndromes with overlapping clinical and pathophysiological features. The production of cytokines in activated NK cells may lead to macrophage overactivation and HLH. HLH should be considered in the presence of unexplained elevated liver function test values or cytopenia with or without other evidence of CRS. Monitoring of C-reactive protein, ferritin, and soluble IL-2R may help in diagnosis. Bone marrow biopsy should be considered to evaluate hemophagocytic. Given the overlap with CRS, patients should be managed according to CRS treatment guidelines (Table 4). Suspected cases of HLH should also be discussed with the medical monitor.

8.2.5 Neurotoxicity

Neurotoxicity has been observed in anti-CD19 CAR T cell therapy (e.g., encephalopathy, lethargy, aphasia). In CAR-NK cell therapy, treatment, and prevention are carried out according to CAR-T's guidelines for neurotoxicity. The grading and management for neurotoxicity were followed by the guidelines listed in Tables 7 and 9, respectively.

Table 7. ASTCT ICANS Consensus Grading

Neurotoxicity Domain	Grade 1	Grade 2	Grade 3	Grade 4
ICE score*	7-9	3-6	0-2	0 (patient is unarousable and unable to perform ICE)
Depressed level Of consciousness#	Awakens spontaneously	Awakens to voice	Awakens only to tactile stimulus	Patient is unarousable or requires vigorous or repetitive tactile stimuli to arouse. Stupor or coma
Seizure	N/A	N/A	Any clinical seizure focal or generalized that resolves rapidly or nonconvulsive seizures on EEG that resolve with intervention	Life-threatening prolonged seizure (>5 min); or Repetitive clinical or electrical seizures without return to baseline in between
Motor findings$	N/A	N/A	N/A	Deep focal motor weakness such as hemiparesis or paraparesis
Elevated ICP/ cerebral edema	N/A	N/A	Focal/local edema on neuroimagingx	Diffuse cerebral edema on neuroimaging; decerebrate or decorticate posturing; or cranial nerve VI palsy; or papilledema; or Cushing's triad

ICANS grade is determined by the most severe event (ICE score, level of consciousness, seizure, motor findings, raised ICP/cerebral edema) not attributable to any other cause; for example, a patient with an ICE score of 3 who has a generalized seizure is classified as grade 3 ICANS.ICE score see Table 6-1.

N/A indicates not applicable.

* A patient with an ICE score of 0 may be classified as grade 3 ICANS if awake with global aphasia, but a patient with an ICE score of 0 may be classified as grade 4

ICANS if unarousable.

Depressed level of consciousness should be attributable to no other cause (eg, no sedating medication).

$ Tremors and myoclonus associated with immune effector cell therapies may be graded according to CTCAE v5.0, but they do not influence ICANS grading.

Intracranial hemorrhage with or without associated edema is not considered a neurotoxicity feature and is excluded from ICANS grading. It may be graded according to CTCAE v5.0.

Table 8. ICE Score

Parameter	Score
Orientation: year, month, city, hospital	4
Naming: ability to name 3 objects (eg, point to clock, pen, button)	3
Following commands: ability to follow simple commands<br>(eg, “show me 2 fingers” or “close your eyes and stick out your tongue”)	1
Writing: ability to write a standard sentence (eg, “our national bird is the bald eagle”)	1
Attention: ability to count backwards from 100 by 10	1

Scoring:

10, no impairment

7-9, grade 1 ICANS

3-6, grade 2 ICANS

0-2, grade 3 ICANS

0 due to patient unarousable and unable to perform ICE assessment, grade 4 ICANS

Table 9. Treatment guidelines for ICANS

Neurotoxicity	Treatment	Evaluation
Grade 1: Examples include:<br>(1) lethargy - mild drowsiness or sleepiness;<br>(2) Blurred consciousness-mild disorientation;<br>(3) Encephalopathy - mildly restrictive ADL;<br>(4) dysplasia - does not affect communication ability;<br>(5) simple partial seizure; Unconscious loss	Vigilant supportive care	Neurological examination<br><br>Perform additional tests based on clinical indications
Grade 2: Examples include:<br>(1) Severe drowsiness and limited instrumental ADL;<br>(2) Confusion and moderate disorientation; Restricted instrument ADL;<br>(3) Encephalopathy - instrumental ADL limitation;<br>(4) Dysplasia-moderate influence on active communication ability; Transient generalized epilepsy.	(1) Vigilant supportive care;<br>(2) Consider prophylactic antiepileptic medication (e.g., levetiracetam 500 mg, BID);<br>(3) If comorbiditis exists (e.g., grade 2 or higher CRS), consider using 8 mg/kg tocilizumab within 1 h (no more than 800 mg).	Brain MRI and cerebrospinal fluid evaluation should be included in addition to neurological examination.<br><br>Ecg is considered as clinical indication.
Grade 3: Examples include:<br>(1) letharg-lethargy or stupor;<br>(2) Blurred consciousness-severe disorientation; Restricted self-care ADL;<br>(3) Limited ADL of encephalopathy-life autorationality;<br>(4) Dysplasia-severe receptivity or emotive traits, affecting the ability to read, write, or communicate clearly, but still having multiple seizures despite drug intervention;<br>(5) Fatigue, limited self-care ADL; Incapacitated;<br>Complete stool/bladder incontinence;	(1) Consider giving tolizumab 8 mg/kg IV (not more than 800 mg) within 1 h;<br>(2) If symptoms are unstable or do not improve, tolizumab should be repeated every 4-6 h;<br>(3) Consider corticosteroids (e.g. Dexamethasone 10 mg IV every 6 h, methylprednisolone 1 mg/kg, BID) if symptoms worsen despite	

	tocilizumab administration;<br>(4) Consider the use of prophylactic antiepileptic drugs.	
Grade 4: Examples include:<br>(1) The consequences are life-threatening;<br>(2) Emergency measures are required;<br>(3) mechanical ventilation;<br>(4) life-threatening, long-term repeated seizures;	Corticosteroids (ex: methylprednisolone 1 g/d × 3 days followed by a rapid decline consisting of 250 mg, BID × 2 days, 125 mg, BID × 2 days, then 60 mg, BID × 2 days).	

8.2.6 Fever and neutropenia

The source of infection should be evaluated according to medication guidelines. Acetaminophe and comfort care should be used to treat fever. Corticosteroids should be avoided.

Subjects with neutropenia and febrile Neutropenia should be treated with broad-spectrum antibiotics. Continuous intravenous fluid replacement (normal saline) should be initiated in most subjects with high fever, especially if oral fluid intake is low or the subject has tachycardia. Filgrastim should be used in accordance with published guidelines (e.g., Infectious Diseases Society of America).

8.2.7 Infection prevention

Subjects should receive prophylactic medications to prevent pneumocystiscarinii pneumonia, herpes virus, and fungal infections in accordance with NCCN guidelines.

8.2.8 B cell aplasia

B cell aplasia and hypogammaglobulinemia may occur due to the effect of CD19-CAR-NK on normal B cells. Gamma globulin will be treated according to the medication guidelines. IgG concentrations should be kept above 400mg/dL at least, especially in the case of infection.

8.2.9 Tumor lysis syndrome

It can occur in patients with rapid tumor cell proliferation and large number of tumor cells death after treatment, generally common in acute leukemia, highly malignant lymphoma, less common in solid tumor patients, such as small cell lung cancer, germ cell malignancies, primary liver cancer and so on. The tumor lysis syndrome has the following characteristics: hyperuricemia, hyperkalemia, hyperphosphatemia resulting in hypocalcemia and other metabolic abnormalities. In a few severe cases, acute renal failure, severe arrhythmias such as ventricular tachycardia and ventricular fibrillation, and DIC (disseminated intravascular coagulation) may also occur. Clinicians should identify patients at high risk of tumor lytic syndrome, strengthen prevention and detection, and immediately start treatment once found.

The tumor lysis syndrome can be prevented by hydration, sodium bicarbonate and drug treatment. The treatment of hyperkalemia is very important: severe patients were first given 10 ml of 10% calcium gluconate solution intravenously, and then increased the intravenous fluid input and the dose of furosemide plus 50% glucose 20 mL and 10 units of insulin. Sodium polystyrene sulfonate resin and sorbitol can also be taken orally, and should not be taken in patients with a history of congestive heart failure or left ventricular dysfunction. In mild cases, increase the amount of intravenous infusion, normal saline and intravenous furosemide (20mg) is sufficient, and sodium bicarbonate can also be used as an intravenous infusion.

8.2.10 Preventing Deep Vein Thrombosis

Preventing Deep Vein Thrombosis (DVT) prophylaxis should be used in all patients with reduced activity during hospitalization in accordance with guidelines. Based on benefit/risk, use of low molecular weight heparin (LMWH) is encouraged as long as there are no contraindications (e.g.,

recent surgery, bleeding quality, platelet count < 50,000/μL). In patients who cannot receive anticoagulants due to the risk of bleeding or other concerns, DVT prophylaxis should be performed with a noninvasive mechanical intermittent pneumatic compression device (Lyman 2015).

8.3 Recording and reporting of adverse events

Adverse events may occur during the treatment of subjects. Once adverse events (including serious adverse events) occur, the occurrence time, clinical manifestations, treatment process and duration, outcome and relationship with drugs should be recorded in detail on the case report form. If laboratory tests are abnormal, patients should be followed up until test results return to normal, or to pre-medication levels, or are determined to be unrelated to the test drug. In case of serious adverse events, the serious Adverse Event form should be filled in and reported to the sponsor, Ethics Committee, CFDA Safety Supervision Department and health administration department within 24 hours.

- Various adverse events: take timely measures to deal with them and record them in the case report form.
- Serious adverse events (SAEs): Timely action is taken, documented on the case report form, withdrawal or reduction of the drug is decided by the investigator, immediately reported to the ethics Committee, the clinical trial facility and the sponsor, and reported to the national and provincial food and drug Administration within 24 hours. SAE is required to log into the Hospital Network Non-Accountability Reporting System for Adverse Events and Near Errors. Specific process: See the Figure below

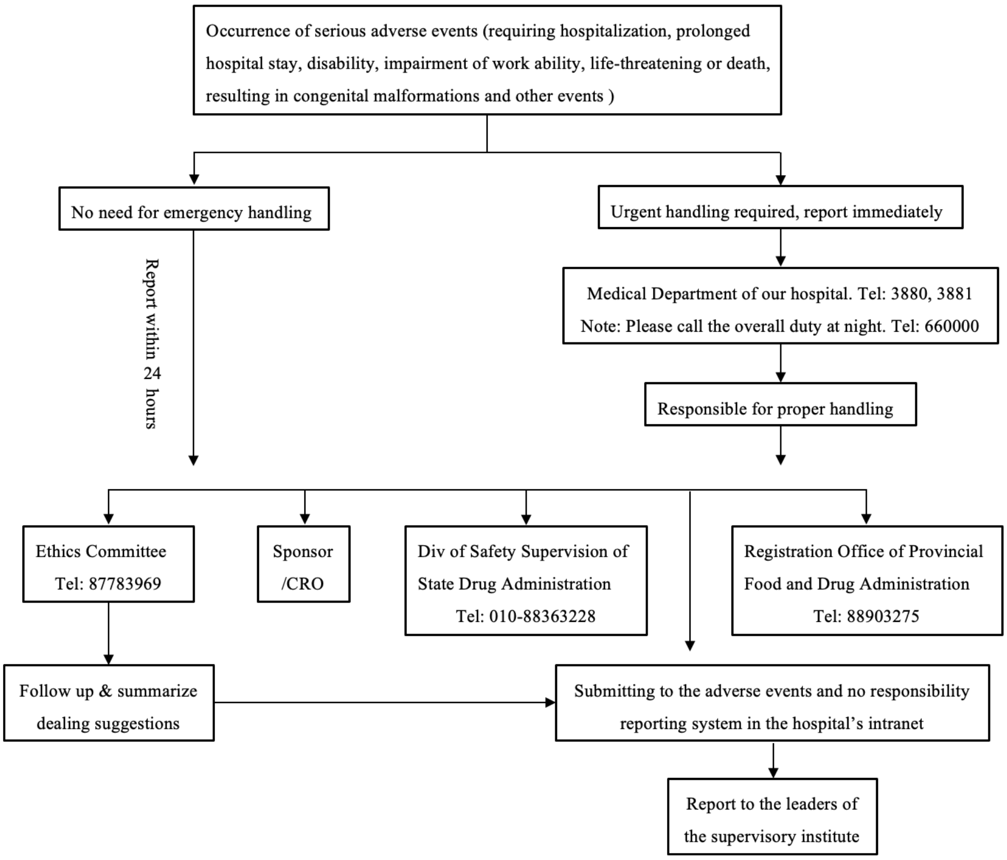

Figure. Flow chart of reporting adverse events in this study
