# Supplementary material for: Safety and feasibility of 4-1BB co-stimulated CD19-specific CAR-NK cell therapy in refractory/relapsed large B cell lymphoma: a phase 1 trial
Source: Nat Cancer. 2025 Apr 18;6(5):786–800. doi: 10.1038/s43018-025-00940-3 (PMC12122374; doi:10.1038/s43018-025-00940-3)
Supplement: Supplementary file 2 — Reporting Summary [file 43018_2025_940_MOESM2_ESM.pdf]

nature portfolio

Corresponding author(s): Wenbin Qian

Last updated by author(s): 26/1/2025

Reporting Summary

Nature Portfolio wishes to improve the reproducibility of the work that we publish. This form provides structure for consistency and transparency in reporting. For further information on Nature Portfolio policies, see our Editorial Policies and the Editorial Policy Checklist.

Statistics

For all statistical analyses, confirm that the following items are present in the figure legend, table legend, main text, or Methods section.

n/a	Confirmed	
☐	☒	The exact sample size (*n*) for each experimental group/condition, given as a discrete number and unit of measurement
☐	☒	A statement on whether measurements were taken from distinct samples or whether the same sample was measured repeatedly
☐	☒	The statistical test(s) used AND whether they are one- or two-sided<br>*Only common tests should be described solely by name; describe more complex techniques in the Methods section.*
☐	☒	A description of all covariates tested
☐	☒	A description of any assumptions or corrections, such as tests of normality and adjustment for multiple comparisons
☐	☒	A full description of the statistical parameters including central tendency (e.g. means) or other basic estimates (e.g. regression coefficient) AND variation (e.g. standard deviation) or associated estimates of uncertainty (e.g. confidence intervals)
☐	☒	For null hypothesis testing, the test statistic (e.g. *F*, *t*, *r*) with confidence intervals, effect sizes, degrees of freedom and *P* value noted<br>*Give P values as exact values whenever suitable.*
☒	☐	For Bayesian analysis, information on the choice of priors and Markov chain Monte Carlo settings
☒	☐	For hierarchical and complex designs, identification of the appropriate level for tests and full reporting of outcomes
☐	☒	Estimates of effect sizes (e.g. Cohen's *d*, Pearson's *r*), indicating how they were calculated

Our web collection on statistics for biologists contains articles on many of the points above.

Software and code

Policy information about availability of computer code

Data collection	Data was collected by ACEA NovoCyte (ACEA Biociences), D 600 Digital PCR System (Maccura), Zeiss LSM 880 confocal laser scanning microscope, 10 × Genomics Chromium controller.
Data analysis	Analyses were performed using GraphPad version 9 software and R version 4.0.3 software. P values <0.05 were considered as significant. For flow cytometry analysis, NovoExpress software(version 1.5.0) was used.

For manuscripts utilizing custom algorithms or software that are central to the research but not yet described in published literature, software must be made available to editors and reviewers. We strongly encourage code deposition in a community repository (e.g. GitHub). See the Nature Portfolio guidelines for submitting code & software for further information.

Data

Policy information about availability of data

All manuscripts must include a data availability statement. This statement should provide the following information, where applicable:

- Accession codes, unique identifiers, or web links for publicly available datasets
- A description of any restrictions on data availability
- For clinical datasets or third party data, please ensure that the statement adheres to our policy

All raw sequencing data generated in this study have been deposited in the National Genomics Data Center (NGDC) under the accession code HRA006106 [https://ngdc.cncb.ac.cn/gsa-human/browse/HRA006106] and HRA008206 [https://ngdc.cncb.ac.cn/search/specific?db=hra&q=HRA008206]. The raw sequencing data are

available under controlled access in accordance with GSA controlled management regulations. Access to the data can be requested by completing the application form via GSA-Human System and is granted by the corresponding Data Access Committee. Additional guidance can be found at the GSA-Human System website [https://ngdc.cncb.ac.cn/gsa-human/document/GSA-Human_Request_Guide_for_Users_us.pdf].
The clinical trial study protocol is available in the Supplementary Information file. Source data are provided with this paper. Individual clinical data cannot be made publicly available for patient privacy, but additional de-identified individual participant clinical data are available from the corresponding author upon request.
The remaining data are available within the Article, Supplementary Information, and Source Data file.

Research involving human participants, their data, or biological material

Policy information about studies with human participants or human data. See also policy information about sex, gender (identity/presentation), and sexual orientation and race, ethnicity and racism.

Reporting on sex and gender: Sex and genders were not considered in our study design. Sex and/or gender was not determined based on self-reporting or assigned and method use.

Reporting on race, ethnicity, or other socially relevant groupings: Race, ethnicity and other socially relevant groupings were not included in our study.

Population characteristics: 8 patients were involved in this study, the median age was 67 years (48 to 73 years).The patients had R/R lymphoma that including diffuse large B-cell lymphoma (DLBCL; 6 cases), transformed follicular lymphoma (tFL; 1 cases), and mantle cell lymphoma (MCL; 1 cases).All patients had undergone particularly heavy pretreatment, with a median of five prior lines (ranging from 3 to 8). Most patients (62.5%) presented with stage III or IV disease, 87.5% had extranodal involvement, 62.5% had elevated lactate dehydrogenase (LDH) levels, and 87.5% had an intermediate or high international prognostic index (IPI) score. The patient with MCL had previously experienced treatment failure with a Bruton's tyrosine kinase (BTK) inhibitor. Notably, three patients had received prior CD19-CAR-T cell therapy.

Recruitment: This phase I trial was a single-center, single-arm and open-label clinical trial. It was open to all refractory and relapse LBCL patients, who refractory or relapse from 2-line therapy, including female and male. The recruitment of patients strictly followed by the eligibility criteria. This clinical trail was registered on clinicaltrials.gov (NCT05472558). The majority of patients were referrals from primary lymphoma doctor at outside institutions or were self-referrals based on the publicly available information or word of mouth through other patients/patients advocates. All these patients minimize the possibility of self-selection bias. Other potential biases and their impact on outcomes may be that some patients had advanced disease and were heavily pre-treated which may influence the assessment of feasibility,safety and antitumor activity.

Ethics oversight: This study was approved by the Ethic Committee of the Second Affiliated Hospital of Zhejiang University School of Medicine and registered ClinicalTrial. gov (NCT05472558, July 21, 2022). This study was also authored by Health Commission of Zhejiang province. All participants provided written informed consent in accordance with the Declaration of Helsinki.

Note that full information on the approval of the study protocol must also be provided in the manuscript.

Field-specific reporting

Please select the one below that is the best fit for your research. If you are not sure, read the appropriate sections before making your selection.

☒ Life sciences ☐ Behavioural & social sciences ☐ Ecological, evolutionary & environmental sciences

For a reference copy of the document with all sections, see nature.com/documents/nr-reporting-summary-flat.pdf

Life sciences study design

All studies must disclose on these points even when the disclosure is negative.

Sample size: The details of the sample size were described in Clinical trail protocol (section 4:Sample Size Considerations)
The complete clinical study protocol included two parts: a dose-escalation period (Phase I) and the dose-expansion period (Phase II). The primary endpoints of Part I were MTD and DLT, approximately 9-18 patients were enrolled according to the "3+3" dose escalation principle. Therefore, 9 to 18 patients were needed in this phase.
The planned dose used in the Phase II is based on the review of the preliminary safety and efficacy observed in Phase I and further differentiates between a treatment with a true response rate of 25% or less and a treatment with a true response rate of 55%. The hypothesis is that the objective response rate to CD19 CAR-NK cells in the R/R LBCL is significantly greater than 30%. Phase II uses a single-arm design to test for improved response rate in the R/R LBCL. For the test of efficacy, this study has ≥ 90% power to distinguish between an active therapy with a 55% ORR from treatment with an ORR of 25% or less with a 1-sided alpha of 0.025, considering a 15% dropout rate, a total of 30 samples is required.

Data exclusions: No data were excluded from analysis

Replication: All pre-clinical experiments were performed at least 2 independent times. Experiments on cell lines were performed three independent times and further validated in two or more cell cell lines.
All in-vivo experiments were performed independently and successfully at least 3 times. All attempts at replication were consistent for all animal and cell culture experiments.

For the CARNK product quality control, such as the transduction efficiency, the immunophenotype, and microbiological detection were assessed at least two independent time points before product release. Every CARNK product before release was tested for cytolytic activity using two CD19 positive cell lines at 4 E:T ratio（3:1,1:1,1:3,1:9). We usually prepared two different derived cord blood for one patient's CARNK manufacturing to ensure the success of infusion and the reproducibility. Disease evaluation with PET-CT was done as part of patient care following standard clinical guidelines. The sc-RNA sequence data of CARNK products and PBMCs from peripheral blood post-infusion were analyzed by two independent bioinformatics groups. Multiplex analysis for serum cytokines was performed by KingMed Diagnostics LTD, which is responsible for the regular clinical diagnosis of patients in the Second Affiliated Hospital of Zhejiang University School of Medicine. ddPCR detecting CARNK in the peripheral blood was done as an independent experiment for each time point. Before starting this study, all SOPs and methods used for CAR-NK product manufacture and release testing were verified more than 10 times to ensure the reproducibility.

Randomization: Not applicable

Blinding: Not applicable

Reporting for specific materials, systems and methods

We require information from authors about some types of materials, experimental systems and methods used in many studies. Here, indicate whether each material, system or method listed is relevant to your study. If you are not sure if a list item applies to your research, read the appropriate section before selecting a response.

Materials & experimental systems

n/a	Involved in the study	
☐	☒	Antibodies
☐	☒	Eukaryotic cell lines
☒	☐	Palaeontology and archaeology
☐	☒	Animals and other organisms
☐	☒	Clinical data
☒	☐	Dual use research of concern
☒	☐	Plants

Methods

n/a	Involved in the study	
☒	☐	ChIP-seq
☐	☒	Flow cytometry
☒	☐	MRI-based neuroimaging

Antibodies

Antibodies used: For CARNK product transduction efficacy and immunophenotype detecting, PB anti-human CD45, clone HI30, Cat#304029, Biolegend, 1:200 dilution, 0.5µL per 100µL testing condition. FITC anti-human CD56, clone 5.1H11, Cat#362546, Biolegend,1:250 dilution, and 0.4µL per 100µL test. PerCP anti-human CD16, clone B73.1, Cat# 302030, Biolegend,1:500 dilution, and used 0.2µL per 100µL test sample. APC-Cy7 anti-human CD3, clone UCHT1, Cat# 300426, Biolegend,1:250 dilution, and used 0.4µL per 100µL test sample. PE-cy7 anti-human CD137L, clone 5F4, Cat# 311512, Biolegend,1:500 dilution, and 0.2µL per 100µL test. AF647 rabbit anti-mouse FMC63 scFv , clone R19M, Cat# 200102, BioSwan Lab,1:500 dilution, and used 0.2µL per 100µL test sample. For CARNK detection in mice tissue, FITC-anti mouse CD45 was used, clone 30-F11, Cat# 103130, Biolegend, 1:250 dilution, and 0.4µL per 100µL test sample. PE anti-human NKp46, clone 9E2, Cat# 331908, 1:500, 0.2µL per 100µL test. Brilliant Violet anti-human CD57, clone QA17A04, Cat# 393314, 1:200, 0.5 µL per 100µL test .For fluorescence staining of CARNK cells in patient's peripheral blood, CD33(Clone S21002C,Cat# 381704, 1:250) and CD14(Clone M5E2, Cat#301804,1:250) were used to excluded the myeloid cells. PerCP Anti-mouse CD45 (Clone 30-F11, CAT# 103130,1:250) was used to in mice experiment.

Validation: All CARNK product and phenotype detection was performed in the Biotherapy Research Center of the Second Affiliated Hospital of Zhejiang University School of Medicine (Certified cGMP facility by State Food and Drug Administration), followed by Practical Flow cytometry in hematology (Yanrong Liu, Version 2). All antibodies utilized have been validated for use in flow cytometry, as detailed on the manufacturer's website. Specifics of the manufacturers, catalog numbers, and dilutions utilized are noted in supplementary table 3.

Eukaryotic cell lines

Policy information about cell lines and Sex and Gender in Research

Cell line source(s): Raji (human Burkitt's lymphoma cell line, TCHu 44) and JeKo-1 (mantle cell lymphoma cell line, TCHu194) cells were obtained from the Cell Bank of the Chinese Academy of Sciences. HEK293T(CRL-3216), NIH/3T3 (CRL-1658) , and K562 (CCL-243-ATC) cells were purchased from the American Type Culture Collection. Cell lines were authenticated by short-tandem-repeat analysis, and regularly tested for mycoplasma contamination.

Authentication: The cell lines we use are maintained as frozen stocks, thawed and maintained for a limited time in culture (<2 months).They were routinely monitored for presence of surface target antigens. The cell lines were authenticated by the STR sequence.

Mycoplasma contamination: Cells were routinely tested for mycoplasma contamination (MycoAlert, Lonza). The cell lines tests negative for mycoplasma prior to use.

Commonly misidentified lines (See ICLAC register)	No cell lines are listed in the database of commonly misidentified cell lines.

Animals and other research organisms

Policy information about studies involving animals; ARRIVE guidelines recommended for reporting animal research, and Sex and Gender in Research

Laboratory animals	6-to 8-week-old female, 18-22g NOD.Cg-Prkdcscid lL2rgtm1Wjl/SzJ Strain (|RRID: IMSR ARC: NSG) mice which carry two mutations on the NOD ShiLtj genetic background, severe combined immune deficiency (scid) and a complete null allele of the IL2 receptor common gamma chain (lL2rg null) were purchased from Biocytogen. All mice were housed at 22 ±1 degree,30-70% relative humidity, in a temperature-controlled BSL-2 facility with 12-hour day-night cycles. Mice were cared for by the husbandry staff at the Second Affiliated Hospital of Zhejiang University animal center, and diet and water were provided on schedule.
Wild animals	The study did not involve wild animals.
Reporting on sex	Female mice were chosen in our study.
Field-collected samples	The study did not involve samples collected from the field
Ethics oversight	All animal studies were approved by the Ethics Committee for Animal Experimentation from the Second Affiliated Hospital of Zhejiang University School of Medicine (Number: AIRB-2021-853) in compliance with Chinese National Laboratory Animal Guideline for Ethical Review of Animal Welfare.

Note that full information on the approval of the study protocol must also be provided in the manuscript.

Clinical data

Policy information about clinical studies

All manuscripts should comply with the ICMJE guidelines for publication of clinical research and a completed CONSORT checklist must be included with all submissions.

Clinical trial registration	This study was registered on the ClinicalTrial. gov (NCT05472558)
Study protocol	Study protocol will be provided as part of this submission.
Data collection	This phase I study has a detailed recruitment and data collection plan in the Clinical trial protocol.<br>1. Screening period: Data collection including informed consent, demographic data, medical history, vital signs, physical examination, combined medication, pregnancy test, blood routine, serum biochemistry, electrocardiogram, bone marrow smear or biopsy, immune function test, imaging examination, etc.<br>2. Follow-up after infusion: follow-up on days 1, 4, 7, 10, 14, 21, and 28 (treatment period): data collection including general information of subjects, ECOG score, symptoms and signs, blood routine, serum biochemistry, electrocardiogram, immune function test, imaging examination, etc. A bone marrow smear or biopsy should be obtained if bone marrow is involved. Peripheral blood was collected to detect the amplification of CARNK.<br>3. Long-term follow-up after infusion. Every 3 months from month 3 to 1 year, and every 6 months for 1 year to 2 years.<br>Data collection includes general information on the subjects, ECOG score, symptoms and signs, routine blood examination, peripheral blood smear examination, electrocardiogram, serum biochemistry, immune function detection, imaging examination, etc.<br>A bone marrow smear or biopsy should be performed if there is bone marrow involvement. Peripheral blood was collected to detect CAR amplification and B cell expression.
Outcomes	The primary endpoint was safety of treatment with CD19-BBz CAR-NK cells, which was determined by the occurrence of DLTs within 30 days of CAR-NK cell infusion. CRS and ICANS were graded with the American Society of Transplantation and Cellular Therapy Consensus grading system, and other AEs were evaluated according to the Common Terminology Criteria for Adverse Events (CTCAE v. 4.03). Secondary endpoint was efficacy and exploration objectives that included the assessment of levels of CAR-NK in blood and cytokines in serum. Response assessment was performed using PET-CT at day 30 according to the Lugano 2014 classification.

Plants

Seed stocks	Not applicable
Novel plant genotypes	Not applicable
Authentication	Not applicable

Flow Cytometry

Plots

Confirm that:

☒ The axis labels state the marker and fluorochrome used (e.g. CD4-FITC).

☒ The axis scales are clearly visible. Include numbers along axes only for bottom left plot of group (a 'group' is an analysis of identical markers).

☒ All plots are contour plots with outliers or pseudocolor plots.

☒ A numerical value for number of cells or percentage (with statistics) is provided.

Methodology

Sample preparation	CAR-NK cells were collected and washed twice with PBS containing 2% FBS. Surface staining was performed for 30 min at 4° Cwith fluorescent conjugated antibodies.
Instrument	Flow analyses were performed with a ACEA NovoCyte flow cytometer (ACEA Biosciences, Inc, San Diego, California, USA).
Software	NovoExpress software(ACEA Biosciences,Inc, San Diego, California, USA, Version 1.5.0).
Cell population abundance	Flow cytometry was used for quantification purposes only.
Gating strategy	Gate strategies for detecting CARNK cells in mice bone marrow, blood, and spleen. Anti-mouse CD45 was used to identify the human derived cells from the mouse host cells. GFP+ cells represented the tumor cells. CD56+ CAR+ NK cells were gated in the human CD45+GFP- cells. Gate strategies for analyzing the transduction efficiency and the phenotype of CARNK products. First FSC-A/SSC-A discriminated viable cells and cell debris; then FSC-H and FSC-A removed the double adhesion cells; CD56+ CAR+-NK cells were gated in the CD45+ cells and CD56/CD16 were identified the phenotypes of CAR-NK cells; CD3+T-cell and CD137L+ feeder cells were also detected in the CD45+ cell to exclude the contamination of T-cell and feeder cells in the CAR-NK products. The expressions of NKp46 and CD57 were detected in CAR+-NK cells. The strategy was used for detecting of the CARNK cells persistence in peripheral blood from patients post-infusion. Density plots from human CD45/SSC were used to gate out the lymphocytes from other cell populations. Myeloid cells were excluded by gating on the CD33 and CD14 negative cells. CD56+CAR+ cells were gated in CD3-CD56+ NK cells from the CD45+ lymphocyte population.

☒ Tick this box to confirm that a figure exemplifying the gating strategy is provided in the Supplementary Information.
